# Miyabeacin: A new cyclodimer presents a potential role for willow in cancer therapy

**DOI:** 10.1038/s41598-020-63349-1

**Published:** 2020-04-15

**Authors:** Jane L. Ward, Yanqi Wu, Claudia Harflett, Hannah Onafuye, Delia Corol, Charlotte Lomax, William J. Macalpine, Jindrich Cinatl, Mark N. Wass, Martin Michaelis, Michael H. Beale

**Affiliations:** 1 Computational and Analytical Sciences Department, Rothamsted Research, West Common, Harpenden, Hertfordshire, AL5 2JQ UK; 2 Present Address: Zhejiang University of Technology, 18 Chaowang Road, Hangzhou, P. R. China; 3 Industrial Biotechnology Centre and School of Biosciences, University of Kent, Canterbury, Kent, CT2 7NJ UK; 4 Institute for Medical Virology, Goethe-University, Frankfurt am Main, Germany

**Keywords:** Secondary metabolism, Solution-state NMR

## Abstract

Willow *(Salix spp.)* is well known as a source of medicinal compounds, the most famous being salicin, the progenitor of aspirin. Here we describe the isolation, structure determination, and anti-cancer activity of a cyclodimeric salicinoid (miyabeacin) from *S. miyabeana* and *S. dasyclados*. We also show that the capability to produce such dimers is a heritable trait and how variation in structures of natural miyabeacin analogues is derived *via* cross-over Diels-Alder reactions from pools of *ortho*-quinol precursors. These transient *ortho*-quinols have a role in the, as yet uncharacterised, biosynthetic pathways around salicortin, the major salicinoid of many willow genotypes.

## Introduction

**Figure 1:**
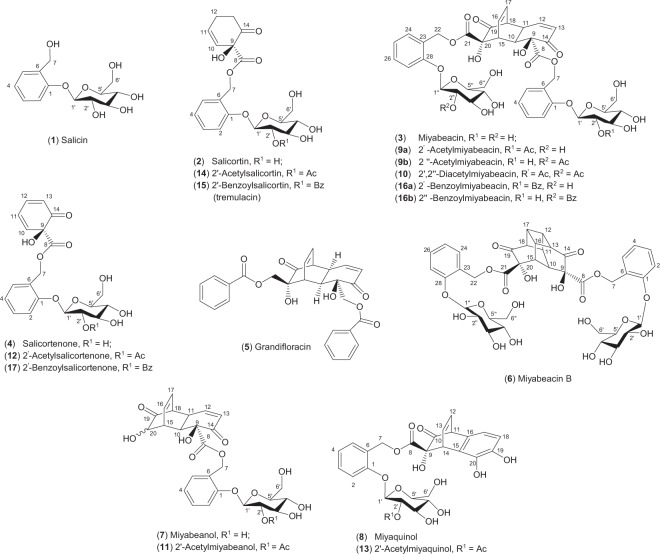
Structures. Carbon numbering system and stereochemical depiction for salicin and salicortin follows that of Feistel *et al*.^2^. For clarity, and to reflect biosynthetic provenance, the salicinoid numbering has been maintained in the numbering of miyabeacin and derivatives.

The utility of willow bark in medicine was recorded by ancient Greek, Assyrian and Egyptian civilisations, but the first scientifically reported investigation of willow (*Salix* spp.) as a remedy for fever was in 1763^1^. The isolation of an active principle, salicin **1** (Fig. 1)^2,3^, was followed by a period of prescription of willow components for pain relief until 1897 when the Bayer Company produced the synthetic analogue, aspirin (acetylsalicylate), one of the earliest and most successful nature-inspired drugs. The medicinal mode of action of aspirin is *via* irreversible acylation of cyclooxygenase isoforms (COX-1 and COX-2) which are key enzymes in prostaglandin biosynthesis^4^. However, both salicin and aspirin are metabolised in humans to salicylic acid, which also inhibits COX *via* a competitive mechanism^5^, which, in part, explains the efficacy of willow bark extracts. However, recent work indicates that salicin itself may have anti-inflammatory properties *via* interaction with other components of the cytokine signalling complex^6^. Besides the established uses of aspirin for pain relief and also its prescription for the mitigation of thrombosis, *via* the involvement of COX-1 in thromboxane A2 mediated platelet aggregation^7^, there is now a renewed interest in the possibility of additional pharmacologies of salicinoids, for example in cancer therapy^8–11^. It has also been suggested that the medicinal activity of herbal extracts of willow cannot be accounted for by the levels of salicin alone^12,13^, indicating a potential for new bio-active or synergistic metabolites. Furthermore, there is now increased clinical interest in utilising willow bark extractives, particularly in the long-term treatment of arthritic pain^14,15^.

The natural salicinoids comprise a sub-group of the wider family of phenolic glycosides that is abundant in the Salicaceae. The known salicinoid structures appear to be derived from variations in acyl group identity and substitution pattern amongst glucose and salicyl hydroxy groups of the salicin base structure^16^. Salicortin **2**, which contains an unusual 1-hydroxy-6-oxocyclohex-2-ene-1-carboxylic ester (HCC) function, usually co-occurs with salicin **1** and together they are found in many species. Surprisingly, the biosynthetic route to these compounds remains largely unknown^17^. Possibly because of the success of aspirin, medicinal assessment of other salicinoids in willow has been mostly neglected by modern science. The potential to discover novel medicinal entities, together with an opportunity to shed light on the biosynthetic pathway, via the identification of candidate intermediates, led us to take an integrated metabolomics approach to define the diversity and dynamics of salicinoid production in willow. In this endeavour, we have been screening members of the UK National Willow Collection, a living resource containing over 1500 diverse *Salix* lines, collected from all over the world. We have recently reported^18^ on salicin-7-sulfate, an analogue that requires further pharmacological investigation as it occurs in those species used in commercial herbal products. In this paper we report on new, structurally intriguing, dimeric salicinoids that have activity against a panel of cancer cell lines. We also demonstrate that the intermolecular Diels-Alder process that leads to this family of molecules is a genetically heritable trait and that the obligate monomeric transient precursor could be a key molecule in the elusive salicortin biosynthetic pathway.

## Results

### Identification, structural determination and bioactivity of miyabeacin 3

**Figure 2:**
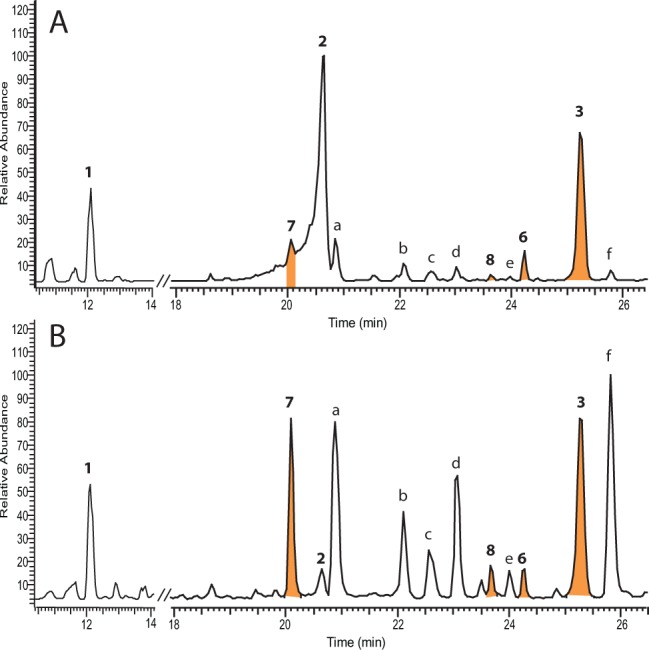
LC-MS data of NWC885 *S. miyabeana* in negative ion mode. (**A**) Total ion chromatogram of juvenile stem tissue; (**B**) Total ion chromatogram of juvenile leaf tissue. Numbered peaks are labelled according to the compound numbering in Fig. 1. Additional peaks labelled a-f are identified as follows: a: luteolin-7-glucoside; b: unknown; c: kaempferol acetyl glucoside isomer; d: kaempferol acetyl glucoside isomer; e: unknown; f: luteolin.

The LC-MS profiles of aqueous methanol extracts of young rapidly growing, stem and leaf tissue of *Salix miyabeana* Seemen “Shrubby”, harvested at growth stage 30–32, according to the published phenological scale^19^ are shown in Fig. 2. The negative ion LC-MS total ion chromatogram of stem tissue was dominated by the known compounds salicin, **1** and salicortin **2** which were also present in leaf samples alongside known flavonoid glycosides. An additional large peak with an [M-H]^−^ ion at *m/z* 843.2353 appeared at 25.26 min. This peak was present in both leaf and stem tissues (Fig. 2) from the same harvest. The accurate mass data (Supplementary Fig. 1) suggested a formula of C_40_H_43_O_20_ for the ion (calculated *m/z* 843.2348) and thus a molecular formula of C_40_H_44_O_20_ for the novel compound **3**. A smaller ion corresponding to the formate adduct (*m/z* 889.2396, C_41_H_45_O_22._) was also observed. Other small ions present in the mass spectrum appeared at *m/z* 557.1300 (C_27_H_25_O_13_), 421.1140 (C_20_H_21_O_10_), 331.1034 (C_14_H_19_O_9_) and 217.0507 (C_12_H_9_O_4_). MS-MS of the precursor ion *m/z* 843.2353 ion did not produce a daughter *m/z* 421 ion indicating that *m/z* 843.2353 was not a [2M-H]^−^ ion formed in the MS-source from a smaller molecule with molecular weight 422. Instead the MS-MS revealed a variety of low abundance fragments including *m/z* 123.0438 (C_7_H_7_O_2_), 183.0463 (C_12_H_7_O_2_), 201.0568 (C_12_H_9_O_3_), 219.0687 (C_12_H_11_O_4_), 227.0364 (C_13_H_7_O_4_), 245.0471 (C_13_H_9_O_5_), 263.0582 (C_13_H_11_O_6_), 289.0362 (C_14_H_9_O_7_) and 557.1374 (C_27_H_25_O_13_) (Supplementary Fig. 2). MS-MS of the small *m/z* 421.1140 ion in the original spectrum provided more insight into the potential structure with clear fragments at *m/z* 297.0626 (C_13_H_13_O_8_), 153.0201 (C_7_H_5_O_4_), 135.0094 (C_7_H_3_O_3_), 123.0447 (C_7_H_7_O_2_), 109.0277 (C_6_H_5_O_2_) and 81.0368 (C_5_H_5_O). This pattern of ions was very similar to that observed from MS-MS of the precursor ion (*m/z* 423) of salicortin **2** (C_20_H_24_O_10_) except for differences of 2 mass units in ions retaining the cyclohexenyl ring substructure (*m/z* 297 *vs* 299; 153 *vs* 155; 81 *vs* 83) (Supplementary Fig. 3). Ions arising from the salicyl portion of the salicortin molecule (*m/z* 123.0447) were retained in **2** and **3**. These MS data together suggested that the novel molecule (MW 844) may contain at least one dehydro-salicortin moiety where the HCC ring now possessed an additional double bond.

For full structure determination by NMR, **3** was isolated *via* a larger scale extraction of *S. miyabeana* tissue and collection of the appropriate peak from semi-preparative HPLC. The ^1^H NMR spectrum of **3** showed peaks relating to 34 coupled hydrogens (Table 1 and Supplementary Fig. 4). Four signals were observed between δ7.34–7.10 and matched those observed for the salicyl ring hydrogens in salicortin (Supplementary Fig. 4). However, in **3** the integration of the aromatic peaks corresponded to 8 hydrogens suggestive of two such salicyl rings. This was confirmed by the presence of two pairs of J = 12 Hz doublet signals relating to the distinctive salicyl hydroxymethylene group (pair 1: δ5.40 and δ5.19; pair 2: δ5.38 and δ5.16). Similarly, the molecule appeared to contain two separate β-glucoside moieties since characteristic doublet signals relating to the H-1′ anomeric hydrogen atoms were duplicated (δ5.09 and δ5.07) as were those corresponding to the glucosyl 6′-methylenes. Unlike the ^1^H-NMR spectrum of salicortin **2** which showed aliphatic signals corresponding to the four methylene hydrogens of the HCC ring between δ2.94 and 2.51, the spectrum of **3** showed no signals in this region indicating a significant change in this part of the molecule. Similarly, signals from the olefinic hydrogens in the HCC-ring that appear in the salicortin ^1^H-NMR spectrum at δ6.27 and δ5.76 each as a doublet of triplets, were absent. In **3** these signals were replaced by four separate olefin signals between δ6.60 and 5.85 each integrating for one hydrogen, two appearing as double doublets and the others as simple triplets. A dienone arrangement was ruled out after examination of the ^1^H-^1^H COSY spectrum (Supplementary Fig. 5) which clearly demonstrated that although two double bonds were present in the molecule, they were isolated from each other. Integration of the carbohydrate region (δ3.96–3.40) in the 1D spectrum suggested a total of sixteen coupled hydrogens. Of these, twelve could be accounted for in two glucose units leaving four unaccounted for. ^13^C NMR data (Table 2 and Supplementary Fig. 6) confirmed the presence of forty carbon atoms in the molecule including two ketone carbonyls at δ198.6 and δ210.0 and two ester carbonyls at δ173.6 and δ173.2 whilst ^13^C DEPT identified four non-aromatic methine signals, in addition to those of glucose (x2) and the two olefins indicated in the ^1^H spectrum (Supplementary Fig. 7). Given the molecular formula from accurate mass, the similarity in fragmentation pattern of the smaller *m/z* 421 fragment to that of salicortin, and the duplication of benzyl and glycosyl related NMR signals we postulated **3** was an unsymmetrical dimeric structure formed via condensation of two molecules of a salicortin analogue with dehydro-HCC rings. To satisfy the observed spectral data we concluded that the dimeric molecule had arisen via a [4 + 2] Diels–Alder cyclodimerisation reaction, between two “salicortenone”, **4** (Fig. 1) monomer units, one acting as the diene, and the second as the dienophile. The deduced structure of **3** has a diketo-1,4-ethenodecalin core, bearing pendant carboxy groups esterified with two salicin units and is shown in Fig. 1 and was named miyabeacin **3**.

**Table 1:** ^1^H-NMR data (600 MHz, D_2_O:CD_3_OD (80:20) referenced to d_4_-TSP (0.01%) at δ0.00) for compounds **2, 3, 6** and **7**. δin ppm; *J* in Hz.

Position	2	3	6	7
1	—	—	—	
2	7.22 (d, 8.1)	7.19 (d, 8.3)	7.20 (d, 8.2)	7.19 (d, 8.0)
3	7.43 (ddd, 8.0, 7.5, 1.9)	7.41 (ddd, 8.0, 7.5, 2.0)	7.43 (ddd, 8.5, 7.5, 1.5)	7.40 (m)
4	7.14 (dt, 7.3, 0.9)	7.12 (t, 7.5)/ 7.11 (t, 7.5)	7.12 (ddd, 7.5, 7.4, 0.9)	7.12 (td, 7.5, 0.9)
5	7.38 (dd, 7.5, 1.6)	7.32 (dd, 7.6, 1.5) / 7.34 (dd, 7.6.1.5)	7.35 (dd, 7.5, 1.5)	7.31 (dd, 7.6, 1.5)
6	—	—	—	—
7α	5.42 (d, 12.2)	5.40 (d, 11.9)	5.46 (d, 11.7)	5.39 (d, 11.9)
7β	5.27 (d, 12.2)	5.19 (d, 11.9)	5.13 (d, 11.7)	5.18 (d, 11.9)
8	—	—	—	—
9	—	—	—	—
10	5.76 (dt, 9.8, 1.6)	3.59–3.63 (m)	2.76 (dd, 4.4, 2.1)	3.57–3.61 (m)
11	6.27 (dt, 10.0, 3.7)	3.58–3.55 (m)	2.99 (m)	3.48–3.53 (m)
12	2.62–2.69 (m) & 2.51–2.57 (m)	6.59 (dd, 10.2, 4.1)	2.88 (m)	6.63 (dd, 10.2, 4.1)
13	2.88–2.94 (m) & 2.57–2.62 (m)	6.02 (dd, 10.2, 1.5)	3.12 (dd, 7.6, 4.0)	6.02 (dd, 10.1, 1.7)
14	—	—	—	—
15	—	3.50–3.53 (m)	2.76 (dd, 4.4, 2.1)	3.28–3.33 (m)
16	—	6.19 (t, 7.9)	2.99 (m)	6.27 (ddd, 7.9, 6.9, 1.0)
17	—	5.91 (ddd, 7.9, 6.5, 1.4)	2.88 (m)	5.94 (1 H, ddd, 7.9, 6.5, 1.4)
18	—	3.43 (m)	3.12 (dd, 7.6, 4.0)	3.36 (1 H, ddd, 6.0, 2.4, 1.4)
19	—	—	—	—
20	—	—	—	—
21	—	—	—	—
22β	—	5.38 (d, 12.1)	5.46 (d, 11.7)	—
22α	—	5.16 (d, 12.1)	5.13 (d, 11.7)	
23	—	—		
24	—	7.32 (dd, 7.6, 1.5)/ 7.34 (dd, 7.6.1.5)	7.35 (dd, 7.5, 1.5)	
25	—	7.12 (t, 7.5)/7.11 (t, 7.5)	7.12 (ddd, 7.5, 7.4, 0.9)	
26	—	7.41 (ddd, 8.0, 7.5, 2.0)	7.43 (ddd, 8.5, 7.5, 1.5)	
27	—	7.20 (d, 8.3)	7.20 (d, 8.2)	
28	—	—		
1′	5.10 (d, 7.6)	5.09 (d, 7.5)/5.07 (d, 7.8)	5.07 (d, 7.8)	5.06 (d, 7.3)
2′	3.56 (m)	3.55–3.63 (m)	3.51 (dd, 9.4, 7.8)	3.49–3.59 (m)
3′	3.57–3.62 (m)	3.56–3.62 (m)	3.58 (m)	3.54–3.61 (m)
4′	3.45–3.50 (m)	3.47–3.52 (m)	3.45 (t, 9.4)	3.45–3.53 (m)
5′	3.57–3.62 (m)	3.56–3.62 (m)	3.58 (m)	3.54–3.61 (m)
6′β	3.74 (dd, 12.6, 5.9)	3.77 (dd, 12.4, 6.0)/3.73 (dd, 12.4, 6.0)	3.72 (dd, 12.4, 6.0)	3.76 (dd, 12.5, 5.9)
6′α	3.92 (dd, 12.6, 2.4)	3.94 (dd, 12.4, 2.1)/3.92 (dd, 12.4, 2.1)	3.99 (dd, 12.5, 2.2)	3.92 (dd, 12.4, 2.2)
1″	—	5.09 (d, 7.5)/5.07 (d, 7.8)	5.07 (d, 7.8)	
2″	—	3.55–3.63 (m)	3.51 (dd, 9.4, 7.8)	
3″	—	3.56–3.62 (m)	3.58 (m)	
4″	—	3.47–3.52 (m)	3.45 (t, 9.4)	
5″	—	3.56–3.62 (m)	3.58 (m)	
6″β	—	3.77 (dd, 12.4, 6.0)/3.73 (dd, 12.4, 6.0)	3.72 (dd, 12.4, 6.0)	
6″α	—	3.94 (dd, 12.4, 2.1)/3.92 (dd, 12.4, 2.1)	3.99 (dd, 12.5, 2.2)

**Table 2:** 13C-NMR data for salicortin 2, miyabeacin 3, miyabeacin B 6 and miyabeanol 7. δin ppm.

Position	2 (D2O:CD_3_OD)	3 (D2O:CD_3_OD)	6 (D2O:CD_3_OD)	7 (D2O:CD_3_OD)	7 (D2O)
1	157.7	158.1	158.1	158.0	157.8
2	117.9	118.0	117.9	117.7	117.3
3	133.3	133.5/133.4	133.9	133.7	133.2
4	125.7	125.7/125.8	125.9	125.7	125.3
5	133.0	133.7/133.6	134.3	133.7	133.2
6	126.8	126.9	126.4	126.6	126.6
7	66.7	67.3	67.1	67.2	67.0
8	173.5	173.6	174.0	173.7	173.6
9	81.0	82.2	80.6	82.5	82.4
10	128.9	40.3	43.1	40.6	40.4
11	136.7	43.5	41.0	43.9	43.7
12	28.7	152.5	37.7	152.8	152.7
13	38.5	131.0	48.1	130.8	130.6
14	199.2	198.6	210.6	199.0	199.1
15	—	45.2	43.1	45.7	45.5
16	—	135.6	41.0	136.0	135.6
17	—	132.8	37.7	132.2	132.2
18	—	54.3	48.1	54.6	54.5
19	—	210.0	210.6	213.3	213.4
20	—	80.0	80.6	missing	81.4
21	—	173.3	174.0	—	—
22	—	66.7	67.1	—	—
23	—	126.7	126.4	—	—
24	—	133.7/133.6	134.3	—	—
25	—	125.7/125.8	125.9	—	—
26	—	133.5/133.4	133.9	—	—
27	—	117.8	117.9	—	—
28	—	157.8	158.1	—	—
1′	102.8	103.1/103.0	102.9	103.0	102.7
2′	75.9	76.1	76.0	76.0	75.8
3′	78.7	79.2	79.0	79.1	78.7
4′	72.2	72.5/72.4	72.8	72.4	72.2
5′	78.7	78.7/78.8	79.0	78.8	78.5
6′	63.4	63.7	63.9	63.6	63.5
1″	—	103.1/103.0	102.9	—	—
2″	—	76.1	76.0	—	—
3″	—	79.2	79.0	—	—
4″	—	72.5/72.4	72.8	—	—
5″	—	78.7/78.8	79.0	—	—
6″	—	63.7	63.9	—	—

Re-analysis of the smaller fragment ions in the mass spectrum of miyabeacin (Supplementary Fig. 1), showed that *m/z* 331.1034 corresponded to the formate adduct of salicin, **1**, *m/z* 421.1140 to the retro-Diels-Alder product “salicortenone” **4**, *m/z* 557.1300 to a mono-desalicylated dimeric moiety and the fragment ion *m/z* 217.0507, corresponding to the cyclo-dimer core structure (C_12_H_9_O_4_) resulting from the loss of both carboxyl side chains. Final confirmation of the structure came from analysis of HSQC and HMBC NMR data (Supplementary Figs. 8 and 9). Key correlations in the ^1^H-^13^C HMBC were observed around all of the positions of the dimeric core structure (Supplementary Table 1) and between H-10 (δ3.60) and C-8 (δ173.6) confirming the attachment of a carboxyl group to the core structure at C-9. The correlation between H-7 (δ5.19 and 5.40) and C-8 (δ173.6) confirmed the linkage *via* the ester carbonyl, to a salicyl moiety. Similar correlations were observed between H-15 (δ3.50) to C-21 (δ173.2) and also between H-22 (δ5.16 and δ5.38) and C-21 (δ173.2), suggesting a second carboxy-salicyl entity attached to the 1,4-ethenodecalin core at C-20. Additional correlations between C-1 (δ158.0) and H-7 (δ5.19 and δ5.40) and H-1′ (δ5.09 / 5.07) and also between C-28 (δ157.7) to H-22 (δ5.16 and δ5.38) and H-1′′ (δ5.07 /5.09) were consistent with placement of the *O*-glucosides at C-1 and C-28.

The structure represents a completely new and unusual salicinoid. However, the aglycone has structural similarity to grandifloracin **5** (Fig. 1), a natural product first isolated from *Uvaria grandiflora* [(−) enantiomer]^20^ and later from *Uvaria dac* [(+) enantiomer] by Awale *et al*., who demonstrated that the molecule had potent activity against human pancreatic cancer cells^21^. The structure of (+)-grandifloracin has been confirmed by both synthesis^22,23^ and by X-ray crystallography^21^. Inspection of the published NMR data^21,23^ relating to the olefin and bridgehead methine hydrogens from grandifloracin agreed well with our observed ^1^H-NMR data of **3** (Supplementary Fig. 10). An important structural difference between miyabeacin **3** and grandifloracin **5** is the oxidation level at C-8 and C-21 (COOH vs CH_2_OH) and hence the orientation of the ester linkage of the attached aromatic ring. The [4 + 2] cyclodimerisation of substituted cyclohexadienones is a well-known reaction in synthetic chemistry and, aside from grandifloracin, there are a number of other examples of this reaction occurring in the natural world^24^. A key feature of the reaction, whether proceeding *in vivo* or *in vitro* is the exquisite regio- and stereo-selectivity leading to entirely *endo*-stereochemistry in the product.

We tested miyabeacin against a range of cancer cell lines. Initial cell viability assays were carried out using the MYCN-amplified neuroblastoma cell line UKF-NB-3, established from a stage 4 neuroblastoma patient^25^ and the vincristine-resistant UKF-NB-3 sub-line UKF-NB-3^r^VCR^10^ (adapted to growth in the presence of vincristine 10 ng/mL). At a concentration of 20 µg/mL of miyabeacin, the cell viability, relative to non-treated cells, after 120 hours was 0% for UKF-NB-3 and 4.22 ± 2.89% for the vincristine resistant UKF-NB-3^r^VCR^10^ line. Initial IC_50_ values were obtained from an expanded range of cell lines including those in neuroblastoma (UKF-NB-3), breast (BT-474 and MCF-7), oesophageal (COLO-680N) and ovarian cancers (COLO-704 and EFO-21) (Table 3) and values ranged from 2.19–27.04 µg/mL. Three cell lines were selected and fully replicated IC_50_ data was obtained (Table 4). Mean values were calculated as 14.47 ± 5.69 µg/mL for the UKF-NB-3 neuroblastoma cell line, 23.87 ± 19.93 µg/mL for the oesophageal cancer cell line COLO-680N and 34.45 ± 12.58 µg/mL for the COLO-704 ovarian cancer cell line.

**Table 3:** IC_50_ assessment of miyabeacin **3** in six cancer cell lines.

Cancer Type	Cell Line	Miyabeacin IC_50_ (μg/mL)
Breast	BT-474	27.04
Oesophageal	COLO-680N	5.08
Ovarian	COLO-704	20.18
Ovarian	EFO-21	12.69
Breast	MCF-7	2.19
Neuroblastoma	UKF-NB-3	7.12

**Table 4:** Replicated IC_50_ determination of miyabeacin **3** in three cancer cell lines.

Cancer Type	Cell Line	Miyabeacin IC50 Concentration (µg/mL)					IC50 (μM)
		*expt 1*	*expt 2*	*expt 3*	*mean*	*S.D*.	*Mean* ± *S.D*.
Oesophageal cancer	COLO-680N	5.08	15.08	51.46	23.87	19.93	**28.28** ± **23.61**
Ovarian cancer	COLO-704	20.18	32.38	50.79	34.45	12.58	**40.18** ± **14.90**
Neuroblastoma	UKF-NB-3	7.12	20.97	15.33	14.47	5.69	**17.15** ± **6.74**

### **Further structurally related natural products in*S. miyabeana***

An isomer of miyabeacin **3**, peak 6 (Fig. 2) with *m/z* 843.2349 (C_40_H_43_O_20_) was present in the LC-MS profile of *S. miyabeana* stem tissue at 24.27 min. MS-MS analysis of this *m/z* 843 ion gave no fragment ions. Isolation of **6** *via* collection of HPLC peaks from repeated injections afforded a pure sample. ^1^H-NMR spectroscopy showed a total of 17 signals which related to 34 separate hydrogens in a highly symmetrical molecule (Table 1 and Supplementary Fig. 11). The presence of signals relating to benzyl and glucosyl moieties compared well with those observed in the ^1^H-NMR spectrum of **3**. Absence of the four olefin signals (δ5.91 to 6.59) previously observed in **3** was accompanied by a movement upfield of the four bridgehead hydrogens (δ3.43–3.63) to give a set of four signals at δ2.76, δ2.88, δ2.99 and δ3.12 each integrating for 2 hydrogens. The ^1^H-NMR data suggested a further [2 + 2] intramolecular cyclization of the olefin units in **3** to give a “caged” structure which we have named miyabeacin B, **6**. The cycloaddition of the double bonds in **3** to yield the cyclobutyl ‘cage’ in **6** now confers a 2-fold axis of symmetry resulting in a significant simplification of the ^1^H-NMR spectrum for **6** relative to that of miyabeacin **3**. This symmetry was also observed in the ^13^C data (Table 2) derived from HSQC and HMBC data (Supplementary Figs. 12, 13). [^1^H-^1^H] correlation spectroscopy confirmed the linkages around the tricyclic core of the molecule as indicated in Supplementary Table 2 and Fig. 14. The [2 + 2] annelation of **3** to give **6** mirrors that observed in laboratory photochemistry of a range of synthetic analogues of the [4 + 2] cyclodimerisation core structure^24^.

A third, structurally related novel compound, miyabeanol **7**, appeared, in the LC-MS, particularly of leaf tissue (Fig. 2), at 20.13 min. The MS spectrum contained three relevant ions at *m/z* 531.1551 (C_26_H_27_O_12_), 467.1159 (C_21_H_23_O_12_) and 421.1120 (C_20_H_21_O_10_). A small ion at *m/z* 217.0507 (C_12_H_9_O_4_) was also present (Supplementary Fig. 15A). The ^1^H NMR spectrum of the HPLC purified compound (Table 1 and Supplementary Fig. 16) suggested an analogous structure to the cyclodimer miyabeacin **3**, although certain regions of the spectrum, including those relating to the benzyl and glucosyl groups, were no longer duplicated suggesting that one of each of these units had been lost. ^1^H signals at δ6.63 and δ6.02 corresponded to those observed in **3** and related to the enone hydrogens, H-12 and H-13. Signals corresponding to the isolated olefin hydrogens at δ6.27 and δ5.94 were also present. These data and additional ^1^H-^1^H COSY correlations of these signals to 4 additional methine hydrogen atoms confirmed that the molecule retained the Diels-Alder “core” present in **3** (Supplementary Table 3 and S17). The NMR data were consistent with the MS data and suggested that compared to **3**, compound **7** (MW 532, C_26_H_28_O_12_) was missing a salicin side chain and a carboxy group. The remaining peaks in the MS spectrum (*m/z* 421.1120 and 467.1159), (Supplementary Fig. 15) were annotated as “salicortenone” **4** and its formate adduct and were believed to have arisen from a retro- Diels Alder fragmentation in the MS source. ^13^C NMR showed 26 separate carbon signals including two ketone signals at δ213.3 and δ199.0 (Table 2). The position of side-chain loss and decarboxylation was confirmed *via* extensive analysis of COSY, HSQC and HMBC correlation spectroscopy (Supplementary Table 3 and Figs. 17–19). Key ^1^H-^13^C correlations were between H-10 and C-8, H-10 to C-14 and H-13 to C-9. This allowed placement of the carboxy-salicylglycoside moiety at C-9. However, no signals or correlations were evident to H-20, suggesting that hydrogen-deuterium exchange, through an ene-diol canonical form, had occurred with the NMR solvent. However, use of 100% D_2_O as solvent (Supplementary Table 3), revealed the peak representing C-20 at 81.4 ppm and correlations from H-16, H-18 and H-10 to C-20 (δ81.4) were all present as well as those from H-15 and H-18 to the carbonyl at C-19.

A related minor compound **8** at 23.67 min in the LC-MS analysis (Fig. 2) displayed an [M-H]^−^ ion at *m/z* 529.1355 corresponding to a molecule with molecular formula C_26_H_26_O_12_, two mass units less than miyabeanol **7**. MS-MS of *m/z* 529 gave a series of daughter ions including some that were two mass units less than the corresponding ions in the MS-MS of **7** (Supplementary Fig. 20). Additionally, an extra fragment ion at *m/z* 159.0458 corresponded to a fragment with a formula of C_10_H_7_O_2._ **8** was isolated by fraction collection from HPLC and the ^1^H NMR spectrum showed a downfield shift of the bridge olefin hydrogens (compared to **7**) and also contained just two methine signals which were also well downfield of those seen in **7** (Supplementary Table 4 and Fig. 21). ^13^C NMR data, derived from HSQC and HMBC experiments (Supplementary Figs. 22, 23) indicated twelve aromatic carbons of which six were consistent with those observed for a salicyl glycoside (Supplementary Table 4). Of the remaining aromatic C-signals, two were methines bearing hydrogens that were *ortho*-coupled (*J* = 8 Hz) to each other only, whilst the other four were quaternary, two of which were observed at δ144.5 and δ147.3 indicating hydroxyl substitution. The deduced structure, miyaquinol **8** (Fig. 1) was confirmed by HMBC correlations to be an *ortho*-substituted di-phenolic ring fused to a bicyclo-octene moiety bearing a ketone at C-10 and the carboxysalicyl glucoside and hydroxyl groups at C-9.

### [4** +** 2] dimers are not confined to Salix miyabeana, and accumulate over the growth cycle

**Figure 3:**
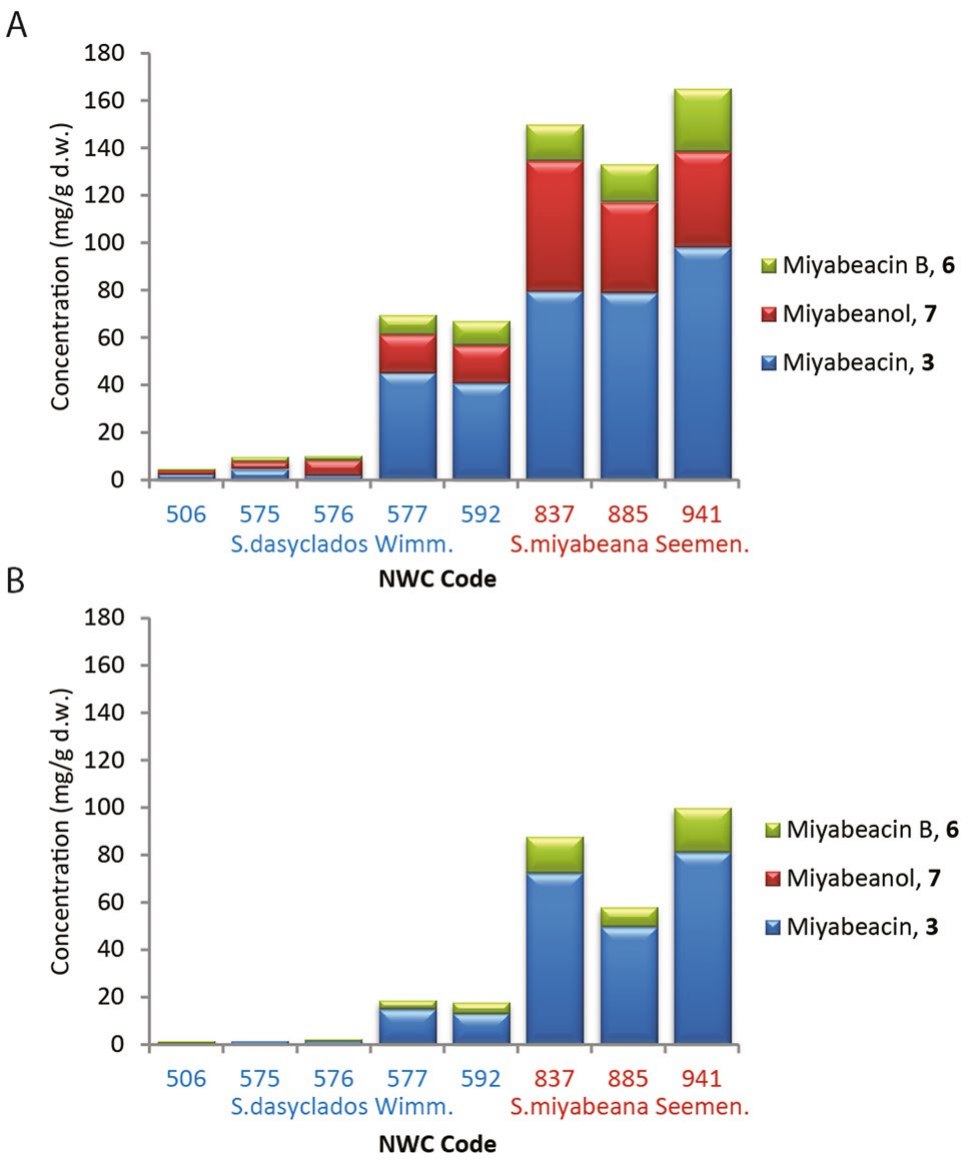
Concentration of miyabeacin, **3**, miyabeacin B, **6** and miyabeanol, **7** in juvenile tissue of *S. dasyclados* and *S. miyabeana* accessions. (**A**) Leaf data; (**B**) Stem data.

Juvenile leaf and stem tissue from 26 field-grown *Salix* species were profiled for the dimeric salicinoids (Supplementary Table 5). Significant levels (>40 mg/g d.w.) of dimeric analogues were found in 3 accessions (NWC941, NWC885 and NWC837) of *S. miyabeana* Seemen, and also in 2 accessions (NWC577 and NWC592) of *S. dasyclados* Wimm. Lower levels were found in 2 additional *S. dasyclados* Wimm. accessions (Fig. 3). Whilst ratios between miyabeacin **3**, miyabeacin B **6** and miyabeanol **7** varied, all compounds were found in leaf tissue. In stem tissue, only **3** and **6** were detected. Of lines that produced dimeric compounds, there was a strong correlation in the concentrations of miyabeacin **3** and miyabeacin B **6** (r^2^ > 0.95, p < 0.001) in both leaf and stem samples suggesting a direct biosynthetic relationship. A slightly lower correlation (r^2^ = 0.86, p < 0.001) between the concentration of miyabeacin **3** and miyabeanol **7** in leaf tissue (Supplementary Fig. 24), was also observed. Comparison of uHPLC-MS peak areas of miyabeanol **7** and **8** indicated a high degree of correlation (r^2^ = 0.982, p < 0.001) and a ratio of approximately 9:1 with **7** being the major of the two products (Supplementary Fig. 25).

**Figure 4:**
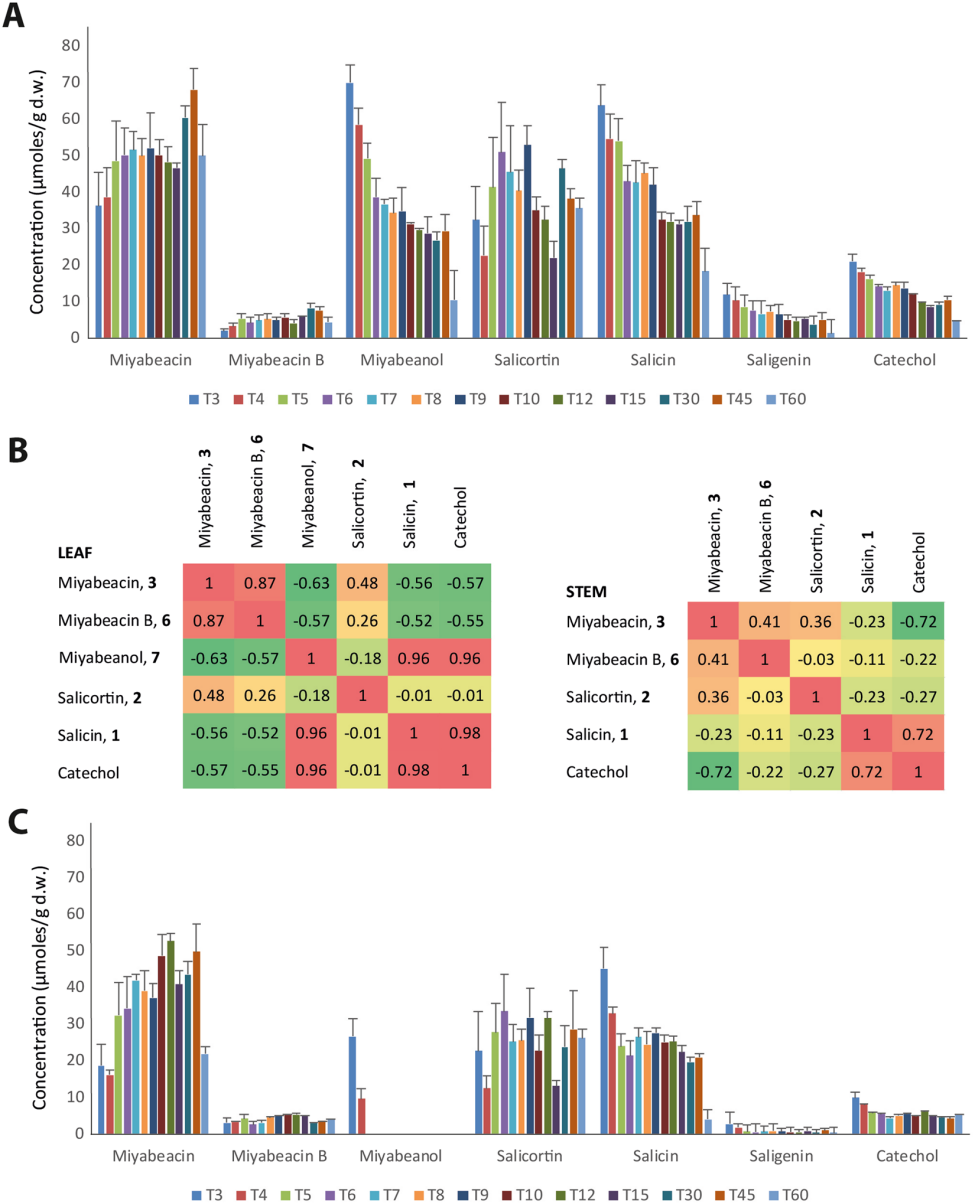
Quantified metabolite concentrations from NWC577 *S. dasyclados* grown in controlled conditions across a 60-day timecourse. Data is coloured according to harvest time with labelling representing the number of days post bud burst. (**A**) leaf data; (**B**) Pearson correlation coefficients for metabolite pairs; (**C**) stem data.

To investigate the onset of dimeric salicinoid production and determine the temporal relationship of the cyclodimerisation with the production of salicortin and salicin, *S. dasyclados* tissue was sampled from 3 to 60 days after budburst (phenological stage 10)^19^ from an array of pot-grown clones generated from cuttings and grown in controlled environment under long days. Destructive sampling was carried out daily at the same time from 3–10 days (T3-T10) and subsequently at 12, 15, 30, 45 and 60 days after bud burst (Supplementary Fig. 26). Compounds **3**, **6** and **7** were quantified from ^1^H-NMR data alongside levels of salicin **1** and salicortin **2**, saligenin (salicyl alcohol) and catechol by integration of compound specific NMR signals. In leaves, miyabeacin **3** and miyabeacin B **6** were present in very young T3 shoots at levels of 36 µmoles/g d.w and 1.9 µmoles/g d.w respectively (Fig. 4A). Levels of these compounds increased from T3 to T8 and then remained level until T15. Levels increased further at T30 and T45 until finally falling at T60. A correlation coefficient of r = 0.87 (p < 0.001) indicated that production of these two dimeric entities were highly correlated (Fig. 4B) and confirmed the hypothesis that **6** is derived from **3** from a [2 + 2] cycloaddition reaction. Levels of salicortin **2** were in the same range (20–50 µmoles/g d.w) but the trajectory was less correlated with **3** and **6** with a coefficient of r = 0.58. Levels of, miyabeanol, **7** were inversely correlated (r = −0.63) with **3**, being highest at T3 (69.9 µmoles/g d.w.) and falling to 10.4 µmoles/g d.w. by T60. Salicin **1** levels were also highest at T3 (63.8 9 µmoles/g d.w.), decreased gradually across the time course and were highly correlated (r = 0.96) with miyabeanol, **7** concentrations. Similarly, catechol and saligenin levels also decreased from T30 to T60 and although observed at lower concentrations were highly correlated with salicin concentrations across the experiment (both having a correlation coefficient of r = 0.98 to salicin). The data indicated a relationship of miyabeanol, **7** to salicin **1**, saligenin and catechol which was in contrast to the increasing levels of miyabeacin, **3** and salicortin **2**.

In stem tissue from the same plant samples the concentration profiles (Fig. 4C) of all metabolites mirrored that observed in leaf tissue with the exception of miyabeanol, **7** which could only be detected in the very juvenile stem tissue of young T3 and T4 shoots. It is possible that **7**, is only produced in leaves and that at the very early time points T3 and T4, the stem and leaf are not yet fully differentiated.

### The cyclodimerization process is heritable and substrate driven giving rise to “cross-over” Diels-Alder products

*S. miyabeana* and *S. dasyclados* have been used as breeding parents for the generation of willow lines with improved biomass traits. The commercial variety “Terra Nova”, a hybrid arising from *S. miyabeana, S viminalis* and *S. triandra* (Supplementary Table 6) was analysed by LC-MS (Supplementary Fig. 27) which confirmed the presence of cyclodimeric analogues **3, 6** and **7** in juvenile leaf tissue. In line with the parent, *S. miyabeana*, cyclodimers were also present in the stem tissue with the exception of **7**. A more striking example of the heritability of the cyclodimer production was observed for the biomass variety “Endurance”, a hybrid of *S. rehderiana* and *S. dasyclados* (Supplementary Table 6), the former a species of willow that produces acetylated salicinoids. The LC-MS profile (Supplementary Fig. 28) again contained compounds **3, 6** and **7** but also contained related compounds all bearing at least one acetyl moiety. Compound **9** gave a peak at 27.21 min and had an [M-H]^−^ ion at *m/z* 885.2456 corresponding to a neutral molecule with a molecular formula of C_42_H_46_O_21._ Smaller ions at *m/z* 421.1142 (C_20_H_21_O_10_) and 463.1244 (C_22_H_23_O_11_) were also present in the spectrum (Supplementary Fig. 29A). Isolation *via* HPLC peak collection and collection of ^1^H-NMR spectra indicated a 1:1 mixture of monoacetylated miyabeacin isomers (**9a** and **9b**), confirmed by the presence of two new 3 H singlets at δ2.143 and δ2.137. Appearance of 2 double doublets (*J* = 8.0, 9.6) at δ5.00 and δ4.97 placed the acetyl moiety in the glucose groups at 2′ in one isomer and at 2′′ in the alternate compound (Supplementary Table 7). Substitution on the glucosyl ring now produced separated signals across the ^1^H spectrum for H-1′/1′′, H-3′/3′′, H-4′/4′′ and H-5′/5′′. Additionally, benzyl hydrogens (H_2_−7) showed an upfield shift due to acetylation at the 2′-position and now appeared at δ5.09/ 5.03 in **9a** while corresponding H_2_−22 moved to δ5.10/ 5.03 in **9b** when acetylation occurred at the 2′′-position. A full ^1^H and ^13^C spectral assignment (Supplementary Table 7) was achieved *via* the use of 2D COSY, HSQC, HMBC and TOCSY experiments and comparison to ^1^H-NMR data from **3** and **7**. Key COSY correlations between the 1′/1′′ and 2′/2′′ peaks confirmed the position of acetylation and further confirmation of the 2′/2′′-acetyl miyabeacin structures **9a** and **9b** was obtained *via* comparison of the MS-MS spectra with that of **3** (Supplementary Fig. 29B,C). Ions at *m/z* 123.0458 (C_7_H_7_O_2_), 201.0571 (C_12_H_9_O_3_), 245.0461 (C_13_H_9_O_5_), 263.0576 (C_13_H_11_O_6_) and 289.0364 (C_14_H_9_O_7_) were identical to those obtained from MS-MS of *m/z* 843. A further ion appearing at *m/z* 599.1435 appears as well as the *m/z* 557.1324 ion observed from MS-MS of **3** and corresponded to an acetylated derivative of this fragment with a formula of C_29_H_27_O_14._ The presence of both of these ions, arising from the loss of a single salicin fragment (Supplementary Fig. 1), is further evidence of a mixture of monoacetylated isomers.

A further compound **10** in Endurance gave an LC-MS peak at 29.05 min and had an [M-H]^−^ ion at *m/z* 927.2537 corresponding to a neutral molecule with molecular formula C_44_H_48_O_22_, suggestive of the diacetyl form of miyabeacin. MS-MS generated a spectrum that was identical to that obtained from **9a/9b** (Supplementary Fig. 30) with the exception of the *m/z* 557 ion which was missing, indicating substitution on each salicin entity. ^1^H-NMR data of **10** (Supplementary Table 8) showed two acetyl singlets at δ2.162 and δ2.156. Separate signals corresponding to 2′-H and 2′′-H appeared at δ4.98 and δ5.01 confirming acetyl substitution at the 2-position of the glucose moiety in both halves of the dimeric molecule.

From Endurance leaf tissue, 2′-acetyl miyabeanol **11** could also be identified from a peak at 23.08 min (Supplementary Fig. 28) with an [M-H]^−^ ion at *m/z* 573.1603 corresponding to a molecular formula for the neutral molecule of C_28_H_30_O_13_ (Supplementary Fig. 31A). As previously observed with **7**, ions arising from an in-source retro Diels-Alder reaction were also observed at *m/z* 463.1243 (C_22_H_23_O_11_) and 509.1295 (C_23_H_25_O_13_) and corresponded to the [M-H]^−^ and formate adducts of 2′-*O*-acetyl “salicortenone”, **12**. MS-MS fragmentation of **11** (Supplementary Fig. 31B) showed an initial loss of an acetyl salicin fragment and then a pattern of ions that followed identical fragmentation to that observed from MS-MS of **7**. The presence of **13**, 2′-acetylmiyaquinol, was indicated in the LC-MS analysis, where 2′-*O*-acetyl salicortin, **14** dominates (Supplementary Fig. 28), but levels were too low for isolation and NMR characterisation.

Further structural diversity was evident in the analysis of a *S. miyabeana* hybrid breeding line (RR10347) generated from a cross of RR05326 (Resolution × NWC609 *S. rossica*) with NWC941 (*S. miyabeana* Purpurescens). Analysis of RR10347 by LC-MS (Supplementary Fig. 32) indicated the presence of tremulacin **15** (C_27_H_28_O_11_), a 2′-O-benzoylated derivative of salicortin which is well known in the Salicaceae^16^. A new peak also appeared at 30.95 min with a mass of 947.2561 that corresponded to an [M-H]^−^ ion from a neutral molecule of molecular formula C_47_H_48_O_21_. As in the case of the mass spectrum of **3**, a smaller ion (*m/z* 421.1125) corresponded to that observed for “salicortenone” **4** (C_20_H_21_O_10_). An additional small ion (*m/z* 525.1465) was also evident with a suggested formula of C_27_H_25_O_11_. MS-MS of *m/z* 947.2561 yielded two strong ions at *m/z* 121 (C_7_H_5_O_2_) and *m/z* 123 (C_7_H_7_O_2_), the former corresponding to a benzoate moiety and the latter to a salicyl moiety. The data were suggestive of **16a/16b**, novel mono-benzoylated derivative of miyabeacin **3**. The peak was isolated by repeated injection into an HPLC system and the structure characterised by ^1^H-NMR (Supplementary Fig. 33). This data, via comparison to the NMR spectrum of **3**, confirmed the presence of a dimeric compound. Additional peaks at δ8.06, δ7.70 and δ7.54 were characteristic of a benzoate group but a doubling of most peaks indicated a 1:1 mixture of isomers which could not be separated further. A set of multiplet peaks appearing at δ5.25 confirmed the benzoyl substitution at the 2′-position of the glucoside in the first isomer (**16a**) and the 2′′-position in the second isomer (**16b**). This was further confirmed in the ^1^H-^1^H -COSY spectrum (Supplementary Fig. 34).

A further example, extending the range of substituted dimeric compounds was seen in the LC-MS analysis of a willow breeding line (RR10147) developed as part of a biomass improvement programme at Rothamsted Research. This hybrid line included *S. dasyclados* (NWC577) in both parents [RR07187 (NWC944 *S. glaucophyloides* × NWC577 *S. dasyclados* “77056”) × RR07188 (NWC944 *S. glaucophyloides* × NWC577 *S. dasyclados* “77056”)] as well as *S. glaucophyloides* (NWC944). In the Total Ion Chromatogram of the negative ion mode LC-MS data (Supplementary Fig. 35) salicortin **2**, 2′-O-acetylsalicortin **14** and tremulacin **15** appeared as major peaks. Given that this cross has generated a hybrid capable of producing both acetylated and benzoylated salicinoids alongside salicortin it followed that associated dimeric analogues would also be expected to be formed *via* a matrix of cross-over reactions involving the three corresponding dienones **2**,**12** and **17**. This was indeed the case with miyabeacin **3** appearing at 25.03 min, 2′/2′′-O-acetylmiyabeacin **9a/9b** appearing at 26.90 min and 2′/2′′-O-benzoylmiyabeacin **16a/16b** appearing at 30.95 min. A further intriguing peak was observed at 32.48 min which showed an ion at *m/z* 989.2617, corresponding to a formula of C_49_H_49_O_22._ Although there was insufficient for isolation, the MS was suggestive of the predicted miyabeacin analogue bearing both an acetyl and benzoyl substitution.

## Discussion

**Figure 5:**
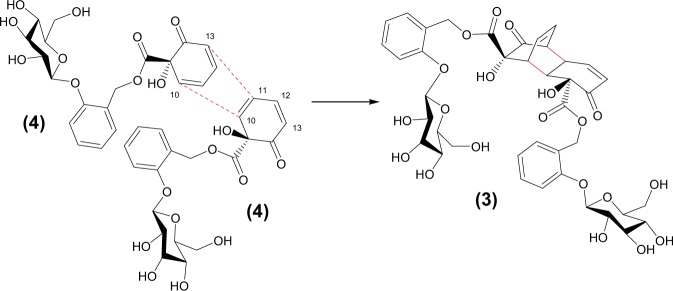
The formation of miyabeacin, **3** *via* cyclodimerization of salicortenone, **4**.

The identification of miyabeacin **3** and its analogues in *Salix spp*. adds further examples to the group of natural products that are formed from intermolecular Diels-Alder, cyclodimerisation reactions of *ortho*-quinonoids^24^. These precedents include dimers of terpenoid and polyketide intermediates, but also the phenolic-derived analogue, grandifloracin **5** which bears strong structural similarities to the aglycone of miyabeacin, although it is important to note that, relative to **5**, the side-chain ester functions of **3** are inversely orientated, and also that in the case of miyabeacin the reaction involves highly polar glycosylated reactants. The obligate substrate for the formation of miyabeacin by cyclodimerisation in willow is the substituted *ortho*-quinol, salicortenone **4** which has a dienone unit that behaves as both a diene and a dienophile, the latter reacting through the distal double bond of the dienone (C-10 and 11) as shown in Fig. 5. This reaction, for these types of orthoquinol reactants, is known to be exquisitely regio- and stereo-specific to give only *endo*- products with no known natural compounds resulting from either *exo*-processes or reaction at the alternate double bond of the dienophile (in this case C12 and 13, Fig. 5.)^24,26^. In this work we have also identified a further dimeric natural product – the ‘cage’ compound, miyabeacin B **6** that arises from an additional [2 + 2] photo-annelation reaction of the [4 + 2] Diels Alder product. There are also precedents, in synthetic examples, for this conversion^24^ which again confirms the *endo* selectivity of the preceding [4 + 2] reaction as the double bonds in the corresponding *exo* products are not aligned for [2 + 2] cage formation. The natural [4 + 2] reaction occurs spontaneously at ambient temperature and, as yet, there has been no evidence provided, for any enyzme directly catalysing intermolecular Diels-Alder reactions^24^. However, we have now presented strong evidence from analysis of breeding parents and progeny that the ability to produce the cyclodimers is a heritable trait that is genetically encoded in *S. miyabeana* and *S. dasyclados* and their progeny. Furthermore, the observation of cross-over [4 + 2] cycloaddition reactions when *Salix* genotypes for example, *S. rehderiana* that produces 2′-acetylsalicortin **14** and *S. rossica* that produces 2′-benzoylsalicortin (tremulacin) **15** are crossed, respectively, with *S. dasyclados* and *S. miyabeana*, provides further compelling evidence for the inheritance of the Diels-Alder capability. An exquisite example of the combinatorial nature of the reactions was observed in the complex hybrids that contain both 2′-acetyl and benzoyl substrates as well as the [4 + 2] capability from *S. dasyclados*, where all possibilities of cross-over dimerisations were observed. These results also indicate that the glucose and substituted glucose moieties in miyabeacin and its analogues must be already present in the dienone substrates, indicating that glycosylation, and 2′-acylation of that glucoside, are earlier steps in the salicinoid pathway.

**Figure 6:**
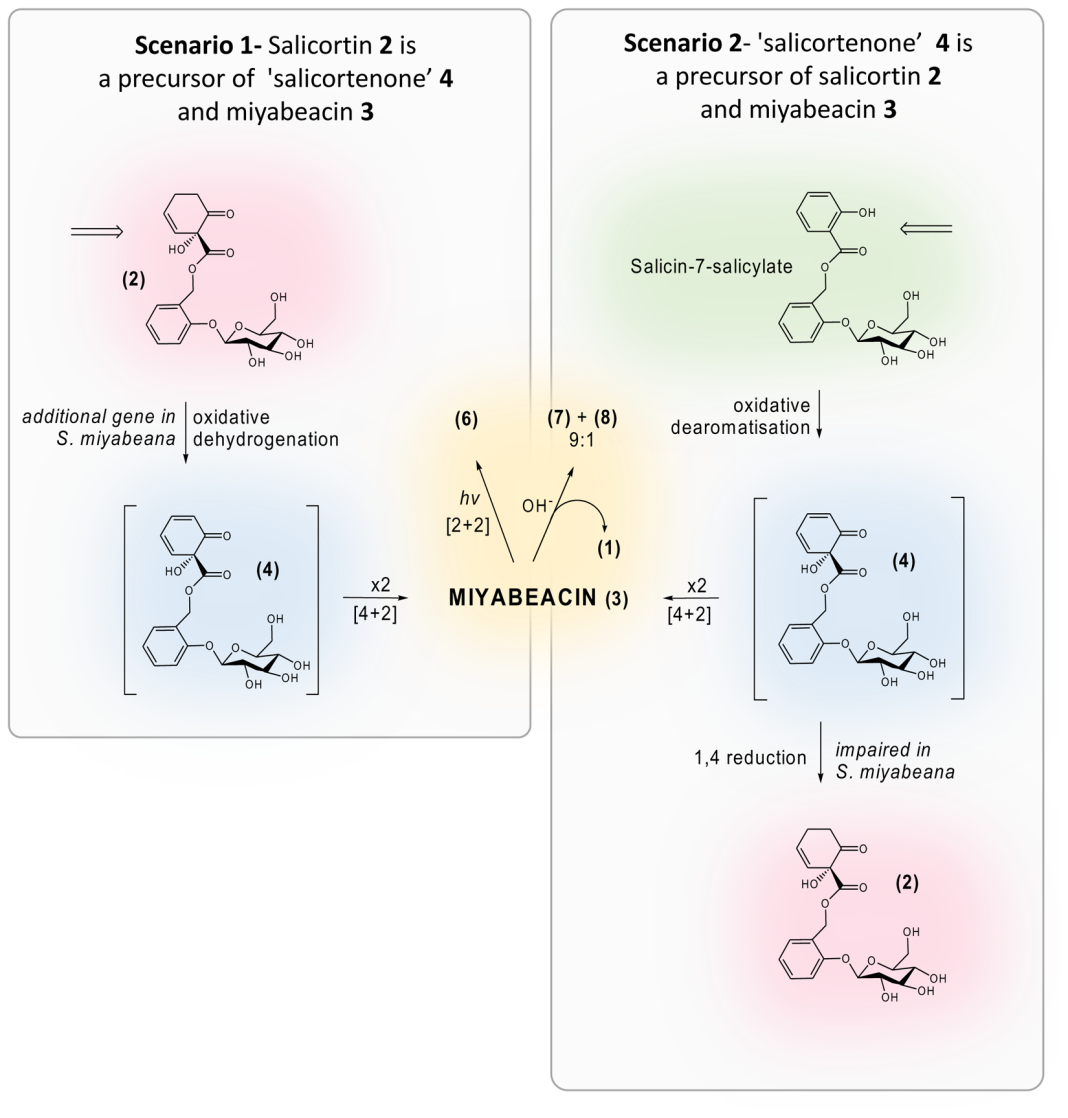
Proposed biosynthetic scenarios leading to miyabeacin, **3** and downstream products.

Apart from the unlikely possibility of the direct involvement of a gene-encoded protein that binds 2 molecules of the monomer salicortenone **4** (and/or its acylated analogues) and facilitates the cyclodimerisation, the most likely explanation of the heritability of the production of miyabeacin and its relatives is that in *S. miyabeana* and *S. dasyclados* there is genetic control of the production or accumulation of the dienones such as **4**. This novel trait/gene is not present or not functional in willow lines that only express the common pathway to salicortin and analogues such as tremulacin. The production of the cross-over [4 + 2] products bearing acyl groups when two analogous salicortenones are co-biosynthesised supports the argument that the pool size of salicortenones is genetically controlled. Given that the spontaneous [4 + 2] dimerisation is under kinetic control, concentrations of precursor *in planta* are a major determinant. Many willow species make salicortin and given the structural similarity of salicortenone to salicortin it is likely that they are closely related biosynthetically. Two biosynthetic scenarios are possible as shown in Fig. 6. The first and more straightforward possibility is that salicortenone **4**, and hence miyabeacin **3**, are metabolites of salicortin **2**, formed from an oxidative dehydrogenation process (e.g. via a P450 reaction) inheritable from *S. miyabeana* or *S. dasyclados*. In this scenario an extra gene, present in *S. miyabeana* or *dasyclados*, is carried into hybrid progeny where, depending on the species, a variety of salicortin derivatives are presented to the new enzyme for conversion to the dienone and hence the dimers. Endurance inherits the [4 + 2] process from *S. dasyclados* and the 2′-acetylation trait from *S. rehderiana*. The observation that mono-acetylmiyabeacins **9a** and **9b** are produced in a 1:1 ratio indicates that pools of “salicortenone” **4** and “acetylsalicortenone” **12** are both available for “cross-over” dimerization reactions, and that, *in planta*, the acetylation of the glucose moieties occurs before, rather than after, the Diels-Alder reaction. The dienones **4** and **12** could not be identified in the samples, and thus remain obligate but undetected biosynthetic intermediates. The biosynthetic precursor or catabolite relationship of salicortin **2** to the structurally close dienone **4** is also unclear, but relative levels of salicortin **2** and 2′-acetylsalicortin **14** in the hybrid may be expected to predict the ratios **3**, **9(9a** + **9b)** and **10** produced in the cross-over reaction. Salicortin **2** and 2′-acetylsalicortin **14** were quantified directly from ^1^H-NMR data of the plant extracts and occurred in a ratio of 6:1 in stems and 1:2 in leaves. Visual inspection of LC-MS analysis of Endurance (Supplementary Fig. 28) shows that the population of cyclo-dimers containing 0,1 or 2 acetyl groups on the glucose moieties do reflect the relative levels of salicortin **2** and 2′-acetylsalicortin **14** in the two tissues. However, the ratios of **3,  9** and **10** are not totally consistent with the notion of simultaneous formation and free mixing of pools of salicortenone **4** and 2′-acetylsalicortenone **12**, which would predict that more **10** than **3** would be produced in leaves where acetylsalicortin **14** predominates over salicortin **2**. The observed ratios of cross-over dimers suggest that the affinity of the oxidative enzyme proposed in scenario 1 (Fig. 6.) may be slightly different for salicortin **2** and the acetyl analogue **14** (and thus also **15**), which can explain why the observed ratio of **3**, **9a** + **9b** and **10** differs from that expected by the relative concentrations of **2** and **14**, particularly in leaf tissue.

The alternate, but more complex, biosynthetic scenario 2 (Fig. 6), begins with oxidative dearomatisation^27^ of salicin-7-salicylate to produce salicortenone **4** as a transient intermediate in the biosynthesis of salicortin **2**. Here we suggest that a “normal” intimate coupling (channelling) between the enzyme(s) that produces **4** and a reductive enzyme, that potentially converts **4** to salicortin *via* a 1,4-reduction process, is impaired in *S. miyabeana* and *S. dasyclados*, resulting in pooling and hence spontaneous dimerisation of a significant proportion of **4**. However, both *S. miyabeana* and *S. dasyclados* still produce salicortin **2**, and thus the reduction process cannot be completely impaired. In this biosynthetic scenario it is this partial loss of reductive function is passed on to the hybrids, a situation that is genetically more complex, but against a background of polypoid genomes, may have more to do with titre of fully coupled and functional reductases. The time-course data (Fig. 4) suggests that salicortin and miyabeacin production *in planta* are linked, but this would be so in both scenarios 1 and 2 (Fig. 6).

The origins of miyabeanol **7** and the minor related miyaquinol **8** which occur only in leaves, and very young green shoots at T3 and T4 (Fig. 4) is also not completely clear. Chemically, **7** and **8** could arise, in the plant or on sample processing, from hydrolytic removal of a salicin **1** moiety from miyabeacin **3** with concomitant decarboxylation (for **7**) or aromatisation via loss of HCOOH (for **8**). We cannot rule out non-enzymic hydrolysis completely but the lack of **7** in mature stem tissue that accumulates **3** indicates that the hydrolysis is not artifactual due to processing, but would need to be specific to photosynthetic tissue, suggesting involvement of a leaf carboxyesterase. *In vitro*, miyabeacin **3** was found to be stable, in neutral aqueous solution for several months, but underwent hydrolysis in mild alkaline solution to give salicin, miyabeanol **7**, miyaquinol **8** and catechol. Thus, another explanation for spatial differences in the occurrence of **7** *in planta*, could be a pH difference in the two tissues. The strong correlation of miyabeanol, salicin and catechol accumulation in the leaf time-course (Fig. 4) indicates that a significant portion of salicin production in *S. miyabeana* and *S. dasyclados* in this tissue occurs through the turnover of miyabeacin *via* miyabeanol. However, in mature stem tissue, where miyabeanol is absent, this route is perhaps less important, and the salicin may arise from the ‘normal’ route shared with other *Salix* genotypes. This ‘normal’ route to salicin is not clear but may involve direct synthesis from salicylaldehyde^28^ or *via* cleavage of esters such as salicin-7-benzoate and/or downstream products such as salicortin^17^. The degree of relocation of molecules from leaf to stem is an unknown factor and it should also be noted that salicortin is also susceptible to hydrolysis *in vitro*, the major products being salicin and catechol^29,30^. Thus, in summary, a network of potential routes to salicin exists, which in *S. miyabeana* and *S. dasyclados* appears to be supplemented by a considerable contribution from miyabeacin catabolism.

The pharmaceutical activity of salicin **1** is well known, but the potential pharmacology of miyabeacin **3** is perhaps much wider. Structurally, it contains two salicin groups that give it potential ‘double dose’ anti-inflammatory and anti-thrombolytic activities associated with salicin and aspirin. However, our results reporting the activity of miyabeacin **3** against a number of cancer cell lines including cell lines with acquired drug resistance, adds further evidence for the multi-faceted pharmacology of willow. Of particular note is the activity against neuroblastoma cell lines. Overall survival rates are below 50% and it represents the most frequent extracranial solid childhood tumour^31^. With resistance acquisition being a significant issue in neuroblastoma^32^, new drugs with novel modes of action are required and miyabeacin perhaps offers a new opportunity in this respect.

## Methods

### Plant tissues

#### Field material

For the initial analyses, multiple juvenile shoots were harvested in May 2014 from the new growth of *S. miyabeana* Seemen “Shrubby” (plot 885 of the National Willow Collection (NWC), Rothamsted Research, UK) that had been coppiced at the end of the previous growing season. Plant tissue from 10 separate plants was combined to generate a homogeneous sample. Tissue was stored at −80 °C prior to lyophilisation. Leaves and stems were separated prior to milling to a fine powder (Retsch Ultra Centrifugal Mill ZM200, Retsch, UK). Milled tissue was maintained at −80 °C until analysis. A voucher specimen has been retained and is available on request. Additional sampling for comparative miyabeacin assessment in other species took place from the same collection within 2 weeks. Tissues were harvested at the same time of day, and were harvested and processed using identical protocols

#### Generation of plant tissue for detailed timecourse

15 cm cutting stems of *S. dasyclados* (NWC577), harvested from the National Willow Collection, Rothamsted Research, UK during dormancy (December 2014), were removed from cold storage (−4 °C), fully defrosted for 6 hours, then soaked overnight in tap water. 50 Cutting stems were planted at half-depth in individual pots (15 cm Ø, 15 cm tall, volume 1.55 L) consisting of a 50:50 v/v of perlite and Rothamsted Standard Compost Mix (75% Medium grade (L&P) peat, 12% Screened sterilised loam, 3% Medium grade vermiculite, 10% Grit (5 mm screened, lime free) plus 3.5 kg Osmocote Exact 3/4 month per m^3^). Plants were grown under controlled environment conditions, set to achieve an average of 600 μmol·m^−2^·s^−1^ measured at plant growth height (2.5 m from light source). Plants were grown under 14/10 hr day/nights at 18 °C/10 °C and 60%/90% relative humidity. Plants were observed daily for signs of budburst and tagged (T0) when they achieved this (normally 7 to 10 days from planting). “Budburst” corresponded with the Weih definition of bud burst (stage 3)^33^ and stage 10 as suggested by Saka and Kuzovkina^19^ 10 whereby “green tip elongates but leaves till remain in a tight cluster (>5 mm)”. Tissue from multiple shoots was cut with scissors and immediately snap-frozen in liquid nitrogen at the following number of days after budburst (T): 3, 4, 5, 6, 7, 8, 9, 10, 12, 15, 30, 45 and 60. For T3-T5 stages tissue from 2 plants (multiple stems) were pooled for each biological replicate. For the remaining timepoints, a single plant’s shoots were harvested. Three biological replicates per timepoint were harvested. Tissue was kept at −80 °C prior to lyophilisation. Stem and leaf tissues were separated prior to milling to a fine powder using a pestle and mortar. Milled tissue was maintained at −80 °C until analysis.

### Metabolite extraction

For ^1^H-NMR and LC-MS metabolomics analysis freeze-dried plant tissues were extracted by established protocols^34–36^. Briefly, ground, freeze-dried tissue aliquots (15 mg) were extracted in H_2_O:MeOH (4:1)(1 mL) for LC-MS and D_2_O:CD_3_OD (4:1 containing 0.01% w/v d_4_−3-(trimethylsilyl)propionic-2,2,3,3 acid)(1 mL) for ^1^H-NMR. Extractions were carried out at 50 °C for 10 min. After cooling and centrifugation, samples were heated to 90 °C for 2 min. Resultant supernatants were transferred to appropriate vials for LC-MS analysis or 5 mm NMR tubes for ^1^H-NMR profiling. For the isolation of pure compounds for structural characterisation, identical procedures were followed. Ratios of plant tissue to extraction solvent are provided in Supplementary Table 9.

### NMR and Mass Spectroscopy

^1^H-1D and ^1^H-^1^H & ^1^H-^13^C 2D-NMR spectra were acquired on a Bruker Avance 600 MHz NMR spectrometer (Bruker Biospin, Germany), operating at 600.05 MHz for ^1^H and 150.9 MHz for ^13^C NMR spectra, using a 5 mm selective inverse probe. Full details (typical scan numbers, parameter sets, pulses, spectral information) are provided in Supplementary Table 10. NMR data was processed using TOPSPIN v. 2.1 (Bruker Biospin, Germany), and ACD NMR Processor (ACD Labs, Toronto, Canada). Metabolite quantitations were carried out using a known concentration of internal standard (d_4_−3-(trimethylsilyl)propionic-2,2,3,3 acid) and characteristic chemical shift regions for each metabolite.

UHPLC–MS spectra were recorded with an Dionex UltiMate 3000 RS UHPLC system, equipped with a DAD-3000 photodiode array detector, coupled to an LTQ-Orbitrap Elite mass spectrometer (Thermo Fisher Scientific, Germany). Chromatographic separation was performed at 35 °C using a reversed-phase Hypersil GOLD™ column (1.9 μm, 30 × 2.1 mm i.d. Thermo Fisher Scientific, Germany). The solvent system consisted of water/0.1% formic acid (A) and acetonitrile/0.1% formic acid (B), both Optima™ grade (Thermo Fisher Scientific, Germany). Separation was carried out for 40 min under the following conditions: 0–5 min, 0% B; 5–27 min, 31.6% B; 27–34 min, 45% B; 34–37.5 min, 75% B. The flow rate was 0.3 mL/min, and the injection volume was 10 μL. Mass spectra were collected in negative mode using an LTQ-Orbitrap Elite with a heated ESI source (Thermo Fisher Scientific, Germany). Full parameters used are provided in Supplementary Table 10. Data was collected and inspected using Xcalibur v. 2.2 (Thermo Fisher Scientific, Germany).

#### Compound isolation

Purification of target compounds was carried out using an HPLC system (Agilent 1100 or Dionex Ultimate 3000) equipped with a quaternary pump, diode array detector, column oven and autosampler. Peaks were separated using an Ascentis C-18 column (5 μm, 5 × 250 mm i.d., Supelco, UK) maintained at 25 °C. Peaks were detected using wavelengths of 254 nm and the peaks corresponding to target compounds were collected, in automation, by time into glass tubes. Solvents were evaporated using a Speedvac concentrator (Genevac, Suffolk, UK). For the generation of bulk extracts, plant tissues were extracted in water:methanol (4:1) using an extraction temperature of 50 °C as described above. Specific details regarding starting tissue weights, extraction volumes, HPLC gradients and amounts of purified compounds generated are given in Supplementary Table 9.

#### Optical rotation

Optical rotation was measured in water on an Anton Paar MCP-100 polarimeter using a 100 mm sample cell. The specific rotation of miyabeacin **3** was determined as [α]_25_^D^ − 59.4 (c 0.155, H_2_O).

### Cell lines

The MYCN-amplified neuroblastoma cell line UKF-NB-3 was established from a stage 4 neuroblastoma patient^25^. The vincristine-resistant UKF-NB-3 sub-line UKF-NB-3^r^VCR^10^ (adapted to growth in the presence of vincristine 10 ng/mL) was established by previously described methods^25^ and derived from the Resistant Cancer Cell Line (RCCL) collection (https://research.kent.ac.uk/industrial-biotechnology-centre/the-resistant-cancer-cell-line-rccl-collection/)^37^. The oesophageal cancer cell line COLO680N was obtained from ATCC (Manassas, VA, USA) and the ovarian cancer (COLO-704 and EFO-21) and breast cancer (BT-474 and MCF-7) cell lines from DSMZ (Braunschweig, Germany). All cell lines were propagated in Iscove’s modified Dulbecco’s medium (IMDM) supplemented with 10% FCS, 100 IU/ml penicillin and 100 mg/ml streptomycin at 37 °C. Cells were routinely tested for mycoplasma contamination and authenticated by short tandem repeat profiling.

### Cell viability assays

Cell viability was determined by the 3-(4,5-dimethylthiazol-2-yl)−2,5-diphenyltetrazolium bromide (MTT) dye reduction assay after 120 h incubation as described previously^38^. Briefly, 5000 cells (suspended in 100 µL IMDM supplemented with 10% FCS, 100 IU/ml penicillin and 100 mg/ml streptomycin) were incubated in 96-well plates at 37 °C and 5% CO_2_ with the investigated extracts for 120 h. MTT solution (25 µL of a 2 µg/mL solution dissolved in PBS) was added for 4 h. This was followed by the addition of sodium dodecyl sulphate solution (100 µL of 20% 50:50 purified water/ DMF) adjusted to pH 4.7 for an additional 4 h in order to lyse cells and dissolve formazan precipitates. Plates were read at 600 nm. The relative viability was determined as the relative reduction of the optical density relative to an untreated cell control (=100%). IC_50_ values were determined using CalcuSyn software (Biosoft, Cambridge, UK).

## Supplementary information

Supplementary Information.
