# Supplementary material for: Miyabeacin: A new cyclodimer presents a potential role for willow in cancer therapy
Source: Sci Rep. 2020 Apr 15;10:6477. doi: 10.1038/s41598-020-63349-1 (PMC7160102; doi:10.1038/s41598-020-63349-1)
Supplement: Supplementary file 1 — Supplementary Information. [file 41598_2020_63349_MOESM1_ESM.docx]

**Supplementary Information**

Jane L. Ward,^,^ Yanqi Wu, Claudia Harflett, Hannah Onafuye, Delia Corol, Charlotte Lomax, William J. Macalpine, Jindrich Cinatl Jr. Mark N. Wass, Martin Michaelis, Michael H. Beale

**Miyabeacin: A new cyclodimer presents a potential role for willow in cancer therapy.**

**Table of Contents**

**Supplementary Table 1.** 1 & 2D-NMR data for Miyabeacin **3** (D2O:CD_3_OD, 80:20 containing d_4_-TSP (0.01% w/v))

**Supplementary Table 2.** 1 & 2D-NMR data for miyabeacin B **6** (D2O:CD_3_OD, 80:20 containing d_4_-TSP (0.01% w/v))

**Supplementary Table 3.** 1 & 2D-NMR data for miyabeanol **7**

**Supplementary Table 4.** 1 & 2D-NMR data for miyaquinol **8** (D2O:CD_3_OD, 80:20 containing d_4_-TSP (0.01% w/v))

**Supplementary Table 5.** Concentrations of dimeric compounds and key salicinoids in juvenile leaf and stem tissue from 26 *Salix* species of the RRes NWC.

**Supplementary Table 6.** Varietal provenance of Terra Nova and Endurance biomass willows.

**Supplementary Table 7.** 1 & 2D-NMR data for acetylmiyabeacin **9a/9b** (D2O:CD_3_OD, 80:20 containing d_4_-TSP (0.01% w/v))

**Supplementary Table 8.** ^1^H-NMR data for diacetylmiyabeacin **10** (D2O:CD_3_OD, 80:20 containing d_4_-TSP (0.01% w/v))

**Supplementary Table 9.** Extraction and HPLC gradient conditions for the isolation of dimeric metabolites.

**Supplementary Table 10.** General Conditions and typical parameters for NMR and Mass Spectral data collection.

**Supplementary Figure 1.** Mass spectrum of miyabeacin **3** at m/z 843.2353 with retention time 25.26 min.

**Supplementary Figure 2.** MSMS data (negative ion mode) of m/z 843 ion of miyabeacin **3**

**Supplementary Figure 3.** MSMS comparison of A: salicortin **2** m/z 423 and B: miyabeacin **3** fragment (m/z 421).

**Supplementary Figure 4.** Comparison of 600 MHz ^1^H-NMR spectra of A: miyabeacin **3** and B: salicortin **2** collected in 80:20 D_2_O:CD_3_OD containing 0.01 % w/v d_4_-TSP.

**Supplementary Figure 5.** COSY45 spectrum of miyabeacin **3**, collected at 600MHz in D_2_O:CD_3_OD (80:20)

**Supplementary Figure 6.** ^13^C spectrum of miyabeacin **3** collected at 400 MHz in D_2_O:CD_3_OD (80:20) containing 0.01% w/v d_4_TSP.

**Supplementary Figure 7.** DEPT135 spectrum of miyabeacin **3**, collected at 100.6128 MHz in D_2_O:CD_3_OD (80:20)

**Supplementary Figure 8.** HSQC spectrum of miyabeacin **3** collected in D_2_O:CD_3_OD (80:20)

**Supplementary Figure 9.** HMBC spectrum of miyabeacin **3** collected in D_2_O:CD_3_OD (80:20)

**Supplementary Figure 10.** Chenomx simulated ^1^H NMR spectrum of grandifloracin in CDCl3 and ^1^H NMR spectrum (δ 6.66 – 5.70) of miyabeacin **3** in D_2_O:CD_3_OD (8:2).

**Supplementary Figure 11.** 600 MHz ^1^H-NMR spectra of A: miyabeacin B **6** collected in D_2_O:CD_3_OD containing 0.01 % w/v d_4_-TSP as reference standard.

**Supplementary Figure 12.** HSQC spectrum of miyabeacin B **6** collected in D_2_O:CD_3_OD containing 0.01 % w/v d_4_-TSP as reference standard.

**Supplementary Figure 13.** HMBC spectrum of miyabeacin B **6** collected in D_2_O:CD_3_OD containing 0.01 % w/v d_4_-TSP as reference standard.

**Supplementary Figure 14.** COSY spectrum of miyabeacin B **6** collected in D_2_O:CD_3_OD containing 0.01 % w/v d_4_-TSP as reference standard.

**Supplementary Figure 15.** MS and MSMS data of miyabeanol **7**.

**Supplementary Figure 16.**  600 MHz ^1^H-NMR spectra of A: miyabeanol **7** collected in D_2_O:CD_3_OD containing 0.01 % w/v d_4_-TSP as reference standard.

**Supplementary Figure 17.**  COSY45 spectrum of A: miyabeanol **7** collected in D_2_O:CD_3_OD containing 0.01 % w/v d_4_-TSP as reference standard.

**Supplementary Figure 18.**  HSQC spectra of miyabeanol **7.** A: D_2_O:CD_3_OD and B: D_2_O

**Supplementary Figure 19.**  HMBC spectra of miyabeanol **7.** A: D_2_O:CD_3_OD and B: D_2_O

**Supplementary Figure 20.** MSMS data of miyaquinol **8**.

**Supplementary Figure 21.**  600 MHz ^1^H-NMR spectra of miyaquinol **8** collected in D_2_O:CD_3_OD containing 0.01 % w/v d_4_-TSP as reference standard.

**Supplementary Figure 22.**  HSQC spectra of miyaquinol **8** collected in D_2_O:CD_3_OD (80:20)

**Supplementary Figure 23.**  HMBC spectra of miyaquinol **8** collected in D_2_O:CD_3_OD (80:20)

**Supplementary Figure 24.**  Pearson correlations of compound concentrations **3**, **6** and **7.**

**Supplementary Figure 25.**  Correlation of uHPLC-MS peak areas for **7** and **8** in *S.* *miyabeana* and *S.* *dasyclados* accessions.

**Supplementary Figure 26.**  Photographs of *S.* *dasyclados* (NWC577) grown in controlled environment conditions.

**Supplementary Figure 27.**  Total ion chromatograms (RT 19.6 – 26.0 min) from uHPLC-MS analyses of polar solvent extracts of Terra Nova (NWC1110).

**Supplementary Figure 28.** Total ion chromatograms (RT 19.6 – 26.0 min) from uHPLC-MS analyses of polar solvent extracts of juvenile willow tissues. A: Endurance stem; B: Endurance leaf; C: *S. dasyclados* (NWC577) stem; D: *S. dasyclados* (NWC 577) leaf; E: *S. rehderiana* (NWC607) stem; F: *S. rehderiana* (NWC607) leaf.

**Supplementary Figure 29.** Mass spectra of acetyl miyabeacin, **9a/9b**, at m/z 885 with retention time 27.21 min.

**Supplementary Figure 30.** MSMS comparison of A: acetyl miyabeacin (m/z 885) **9** and B: diacetyl miyabeacin (m/z 927) **10**.

**Supplementary Figure 31.** MS and MSMS data (negative ion mode) for 2′acetyl miyabeanol **11**.

**Supplementary Figure 32**. LC-MS data (negative ion mode) of RR10347 (RR05326 (Resolution × *S. rossica*) × NWC941 (*S. miyabeana* Purpurescens)).

**Supplementary Figure 33**. ^1^H-NMR data of 2′-Benzoylmiyabeacin/2′′-O-Benzoylmiyabeacin **16a/16b** collected at 600 MHz in D_2_O:CD_3_OD (4:1).

**Supplementary Figure 34.** ^1^H-^1^H COSY NMR data of 2′-Benzoylmiyabeacin/2′′-O-Benzoylmiyabeacin **16a/16b** collected at 600 MHz in D_2_O:CD_3_OD (4:1).

**Supplementary Figure 35**. LC-MS data of RR10147 (RR07187 (944 *S. glaucophyloides* × 577 “77056”) × RR07188 (944 *S. glaucophyloides* × 577 “77056”)).

**Supplementary Table 1.** 1 & 2D-NMR data for Miyabeacin **3** (D2O:CD_3_OD, 80:20 containing d_4_-TSP (0.01% w/v)) δ in ppm relative to d_4_-TSP at 0.00.

| Position | ^1^H (ppm) | *J*_H-H_ (Hz) | ^13^C (ppm) | ^1^H-^1^H correlation to: | ^1^H-^13^C HMBC correlation to: |
| --- | --- | --- | --- | --- | --- |
| 1 | - |  | 158.0 | - |  |
| 2 | 7.19 (1H, d) | 8.3 | 117.9 | H-3, H-4 & H-5 | 126.9 (C-6) & 158.0 (C-1) |
| 3, 26 | 7.41 (2H, ddd) | 8.0, 7.5, 2.0 | 133.5/133.4 | H-2, H-4, H-25 & H-27 | 158.0 (C-1)/157.7 (C-28) & 133.7 (C-C-5)/133.6 (C-24) |
| 4, 25 | 7.12 (1H, t)/7.11 (1H, t) | 7.5 | 125.6/125.7 | H-3, H-5, H-24 & H-26 | 117.9 (C-2)/117.7 (C-27) & 126.9 (C-6)/126.6 (C-23) |
| 5,24 | 7.32 (1H, dd)/ 7.34 (1H, dd) | 7.6, 1.5 | 133.7/133.6 | H-3, H-4, H-25 & H-26 | 158.0 (C-1)/157.7 (C-28), 133.5 (C-3)/133.4 (C-26) & 67.3 (C-7)/66.7 (C-22) |
| 6 | - |  | 126.9 | - |  |
| 7α | 5.40 (1H, d) | 11.9 | 67.3 | H-7β | 126.9 (C-6), 133.7 (C-5), 158.0 (C-1), 173.6 (C-8) |
| 7β | 5.19 (1H, d) | 11.9 | 67.3 | H-7α | 126.9 (C-6), 133.7 (C-5), 158.0 (C-1), 173.6 (C-8) |
| 8 | - |  | 173.6 | - |  |
| 9 | - |  | 82.2 | - |  |
| 10 | 3.59-3.63 (1H, m) |  | 40.3 | H-11 & H-15 | 43.5 (C-11), 45.1 (C-15), 135.5 (C-16), 152.5 (C-12), 173.6 (C-8), 198.6 (C-14) |
| 11 | 3.58-3.55 (1H, m) |  | 43.5 | H-10, H-12 & H-18 | -  NB – also observed in literature data |
| 12 | 6.59 (1H, dd) | 10.2, 4.1 | 152.5 | H-11 & H-13 | 40.3 (C-10), 43.5 (C-11), 54.2 (C-18), 198.6 (C-14) |
| 13 | 6.02 (1H, dd) | 10.2, 1.5 | 130.9 | H-12 | 43.5 (C-11), 82.2 (C-9) |
| 14 |  |  | 198.6 | - |  |
| 15 | 3.50-3.53 (1H, m) |  | 45.1 | H-16 | 43.5 (C-11), 80.0 (C-20), 82.2 (C-9), 132.8 (C-17), 135.5 (C-16), 173.2 (C-21), 210.0 (C-19) |
| 16 | 6.19 (1H, t) | 7.9 | 135.5 | H-15 & H-17 | 45.1 (C-15), 54.2 (C-18), 80.0 (C-20) |
| 17 | 5.91 (1H, ddd) | 7.9, 6.5, 1.4 | 132.8 | H-16 & H-18 | 45.1 (C-15), 54.2 (C-18), 210.0 (C-19) |
| 18 | 3.43 (1H, m) |  | 54.2 | H-11, H-16 & H-17 | 40.3 (C-10), 80.0 (C-20), 132.8 (C-17), 135.5 (C-16), 210.0 (C-19) |
| 19 | - |  | 210.0 | - |  |
| 20 | - |  | 80.0 | - |  |
| 21 | - |  | 173.2 | - |  |
| 22β | 5.38 (1H, d) | 12.1 | 66.7 | H-22α | 126.6 (C-23), 133.7/133.6 (C-24), 157.7 (C-28), 173.2 (C-21) |
| 22α | 5.16 (1H, d) | 12.1 | 66.7 | H-22β | 126.6 (C-23), 133.7/133.6 (C-24), 157.7 (C-28), 173.2 (C-21) |
| 23 | - |  | 126.6 |  |  |
| 27 | 7.20 (1H, d) | 8.3 | 117.7 | H-24, H-25 & H-26 | 126.6 (C-23), 157.7 (C-28) |
| 28 | - |  | 157.7 | - |  |
| 1’, 1’’ | 5.09 (1H, d)/5.07 (1H, d) | 7.5/7.8 | 103.0/102.9 | H-2’/H-2’’ | 158.0/157.7 (C-1)/(C-28) |
| 2’, 2’’ | 3.55-3.63 (2H, m) |  | 76.0 | H-1’ & H-3’/H-1’’ & H-3’’ | 103.0/102.9 (C-1’)/(C-1’’), 78.7/78.8 (C-5’)/(C-5’’) |
| 3’, 3’’ | 3.56-3.62 (2H, m) |  | 79.1 | H-2’ & H-4’/H-2’’ & H-4’’ | 72.5/72.4 (C-4’)/(C-4’’) |
| 4’, 4’’ | 3.47-3.52 (2H, m) |  | 72.5/72.4 | H-3’ & H-5’/H-3’’ & H-5’’ | 63.7 (C-6’)/(C-6’’), 78.8/78.8 8 (C-5’)/(C-5’’) |
| 5’, 5’’ | 3.56-3.62 (2H, m) |  | 78.7/78.8 | H-4’ & H-6’/H-4’’ & H-6’’ | 63.7 (C-6’)/(C-6’’), 73.0 (C-4’)/(C-4’’) |
| 6’β, 6’’β | 3.77 (1H, dd)/3.73 (1H, dd) | 12.4, 6.0 | 63.7 | H-6’α/H-6’’α | 78.9 (C-5’)/(C-5’’) |
| 6’α, 6’’α | 3.94 (1H, dd)/3.92 (1H, dd) | 12.4, 2.1 | 63.7 | H-6’β/H-6’’β | 72.5/72.4 (C-4’)/(C-4’’), |

**Supplementary Table 2.** 1 & 2D-NMR data for miyabeacin B **6** (D2O:CD_3_OD, 80:20 containing d_4_-TSP (0.01% w/v)) δ in ppm relative to d_4_-TSP at 0.00.

| Position | ^1^HNMR(ppm) | *J*_H-H_ (Hz) | ^1^H-^1^H correlation to: |
| --- | --- | --- | --- |
| 1 & 28 | - | - | - |
| 2 & 27 | 7.20 (2H, d) | 8.2 | H-3 & H-26 |
| 3 & 26 | 7.43 (2H, ddd) | 8.5, 7.5, 1.5 | H-2, H-4, H-27 & H-25 |
| 4 & 25 | 7.12 (2H, ddd) | 7.5, 7.4, 0.9 | H-3, H-5, H-26 & H-24 |
| 5 & 24 | 7.35 (2H, dd) | 7.5, 1.5 | H-4 & H-5 |
| 6 & 23 |  | - | - |
| 7β & 22β | 5.13 (2H, d) | 11.7 | H-7α & H-22α |
| 7α & 22α | 5.46 (2H, d) | 11.6 | H-7β & H-22β |
| 9 & 20 |  | - | - |
| 10 & 15 | 2.76 (2H, dd) | 4.4, 2.1 | H-11 & H-16 |
| 11 & 16 | 2.99 (2H, m) |  | H-10, H-12, H-15 & H-17 |
| 12 & 17 | 2.88 (2H, m) |  | H-11, H-13, H-16 & H-18 |
| 13 & 18 | 3.12 (2H, dd) | 7.6, 4.0 | H-11, H-12, H-16 & H-17 |
| 1’ & 1’’ | 5.07 (2H,d) | 7.8 | H-2’/H-2’’ |
| 2’& 2’’ | 3.51 (2H, dd) | 9.4, 7.8 | H-1’ & H-3’/H-1’’ & H-3’’ |
| 3’ & 3’’ | 3.45 (2H, t) | 9.4 | H-2’ & H-4’/H-2’’ & H-4’’ |
| 4’,4’’, 5’ & 5’’ | 3.58 (4H, m) |  | H-3’ & H-3’’ & H-6’ & H-6’’ |
| 6’β & 6’’β | 3.72 (2H, dd) | 12.4, 6.0 | H-5’ & H-5’’  H-6’α/H-6’’α |
| 6’α & 6’’α | 3.99 (2H, dd) | 12.5, 2.2 | H-5’ & H-5’’  H-6’β/H-6’’β |

**Supplementary Table 3.** 1 & 2D-NMR data for miyabeanol **7** δ in ppm relative to d_4_-TSP at 0.00.

|  | D2O:CD_3_OD | | | | D_2_O | | | |
| --- | --- | --- | --- | --- | --- | --- | --- | --- |
| Position | δ_H_ (ppm) | δ_C_ (ppm) | ^1^H-^1^H correlation to: | ^1^H-^13^C HMBC correlation to: | δ_H_ (ppm) | δ_C_ (ppm) | ^1^H-^1^H correlation to: | ^1^H-^13^C HMBC correlation to: |
| 1 | - | 158.0 | - |  | - | 157.8 | - | - |
| 2 | 7.19 (1H, d, 8.0) | 117.7 | H-3 | 126.6 (C-6),158.0 (C-1) | 7.19 (1H, d, 7.9) | 117.3 | H-3 | 126.6 (C-6),157.8 (C-1) |
| 3 | 7.40 (1H, m) | 133.7 | H-2 & H-4 | 158.0 (C-1), 133.7 (C-5) | 7.42 (1H, m) | 133.2 | H-2 & H-4 | 157.8 (C-1), 133.2 (C-5) |
| 4 | 7.12 (1H, td, 7.5, 0.9) | 125.7 | H-3 & H-5 | 117.7 (C-2), 126.6 (C-6), 133.7 (C-3), 158.0 (C-1) | 7.12 (1H, td, 7.5, 0.9) | 125.3 | H-3 & H-5 | 117.3 (C-2), 126.6 (C-6), 133.2 (C-3), 157.8 (C-1) |
| 5 | 7.31 (1H, dd, 7.6, 1.5) | 133.7 | H-4 | 158.0 (C-1), 133.7 (C-3) , 117.7 (C-2), 67.2 (C-7) | 7.34 (1H, dd, 7.6, 1.5) | 133.2 | H-4 | 157.8 (C-1), 133.2 (C-3) , 117.3 (C-2), 67.0 (C-7) |
| 6 |  | 126.6 |  |  | - | 126.6 | - | - |
| 7α | 5.39 (1H, d, 11.8) | 67.2 | H-7β | 126.6 (C-6), 133.7 (C-5), 158.0 (C-1), 173.7 (C-8) | 5.41 (1H, d, 11.9) | 67.0 | H-7β | 126.6 (C-6), 133.2 (C-5), 157.8 (C-1), 173.6 (C-8) |
| 7β | 5.18 (1H, d, 11.8) | 67.2 | H-7α | 126.6 (C-6), 133.7 (C-5), 158.0 (C-1), 173.7 (C-8) | 5.18 (1H, d, 11.9) | 67.0 | H-7α | 126.6 (C-6), 133.2 (C-5), 157.8 (C-1), 173.6 (C-8) |
| 8 | - | 173.7 | - |  | - | 173.6 | - | - |
| 9 | - | 82.5 | - |  | - | 82.4 | - | - |
| 10 | 3.57-3.61 (1H, m) | 40.6 | H-11 & H- 15 | 43.9 (C-11), 45.7 (C-15), 136.0 (C-16), 152.8 (C-12), 173.7 (C-8), 199.0 (C-14) | 3.63 (1H, m) | 40.4 | H-11 & H- 15 | 43.7 (C-11), 45.5 (C-15), 81.4 (C-20), 135.6 (C-16), 152.7 (C-12), 173.6 (C-8), 199.1 (C-14) |
| 11 | 3.48-3.53 (1H, m) | 43.9 | H-10, H-12 & H-18 | 152.8 (C-12) | 3.55 (m) | 43.7 | H-10, H-12 & H-18 | 152.7 (C-12) |
| 12 | 6.63 (1H, dd, 10.2, 4.1) | 152.8 | H-13 & H-11 | 40.6 (C-10), 43.9 (C-11), 54.6 (C-18), 199.0 (C-14) | 6.64 (1H, dd, 10.2, 4.2) | 152.7 | H-13 & H-11 | 40.4 (C-10), 43.7 (C-11), 54.5 (C-18), 199.1 (C-14) |
| 13 | 6.02 (1H, dd, 10.1, 1.7) | 130.8 | H-12 & H-10 | 43.9 (C-11), 82.5 (C-9) | 6.05 (1H, dd, 10.2, 1.8) | 130.6 | H-12 & H-10 | 43.7 (C-11), 82.4 (C-9) |
| 14 | - | 199.0 |  |  | - | 199.1 | - | - |
| 15 | 3.28-3.33 (1H, m) | 45.7 | H-16 & H-10 | 40.6 (C-10), 43.9 (C-11), 82.5 (C-9), 132.2 (C-17), 136.0 (C-16), 213.3 (C-19) | 3.38 (1H, dt, 6.5, 1.7) | 45.5 | H-16 & H-10 | 40.4 (C-10), 43.7 (C-11), 81.4 (C-20), 82.4 (C-9), 132.2 (C-17), 135.6 (C-16), 213.4 (C-19) |
| 16 | 6.27 (1H, ddd, 7.9, 6.9, 1.0) | 136.0 | H-17 & H-15 | 45.7 (C-15), 54.6 (C-18), 82.5 (C-9), 213.3 (C-19) | 6.29 (1H, ddd, 7.8, 6.4, 1.0) | 135.6 | H-17 & H-15 | 45.5 (C-15), 54.5 (C-18), 81.4 (C-20), 82.4 (C-9), 213.4 (C-19) |
| 17 | 5.94 (1H, ddd, 7.9, 6.5, 1.4) | 132.2 | H-16 & H-18 | 45.7 (C-15), 54.6 (C-18), 213.3 (C-19) | 5.99 (1H, ddd, 8.0, 6.3, 1.5) | 132.2 | H-16 & H-18 | 45.5 (C-15), 54.5 (C-18), 81.4 (C-20), 213.4 (C-19) |
| 18 | 3.36 (1H, ddd, 6.0, 2.4, 1.4) | 54.6 | H-17 & H-11 | 40.6 (C-1), 82.5 (C-9), 132.2 (C-17), 136.0 (C-16), 213.3 (C-19) | 3.41 (1H, ddd, 6.1, 2.3, 1.3) | 54.5 | H-17 & H-11 | 40.4 (C-1), 81.4 (C-20), 82.4 (C-9), 132.2 (C-17), 135.6 (C-16), 213.4 (C-19) |
| 19 | - | 213.3 | - |  | - | 213.4 | - | - |
| 20 | missing | missing | - |  | Absent (d-exchange) | 81.4 | - | - |
| 1’ | 5.06 (1H, d, 7.3) | 103.0 | H-2’ | 158.0 (C-1) | 5.10 (1H, d, 7.7) | 102.7 | H-2’ | 157.8 (C-1) |
| 2’ | 3.49-3.59 (1H, m) | 76.0 | H-1’ & H-3’ | 103.0 (C-1’), 78.8 (C-5’) | 3.49-3.59 (1H, m) | 75.8 | H-1’ & H-3’ | 102.7 (C-1’), 78.5 (C-5’) |
| 3’ | 3.54-3.61 (1H, m) | 79.1 | H-2’ & H-,4’ | 72.4 (C-4’) | 3.54-3.61 (1H, m) | 78.7 | H-2’ & H-,4’ | 72.2 (C-4’) |
| 4’ | 3.45-3.53 (1H, m) | 72.4 | H-3’ & H-5’ | 63.6 (C-6’), 78.8 (C-5’), 103.0 (C-1’) | 3.45-3.53 (1H, m) | 72.2 | H-3’ & H-5’ | 63.5 (C-6’), 78.5 (C-5’), 102.7 (C-1’) |
| 5’ | 3.54-3.61 (1H, m) | 78.8 | H-4’ & H-6’ | 63.6 (C-6’), 76.0 (C-2’) | 3.54-3.61 (1H, m) | 78.5 | H-4’ & H-6’ | 63.5 (C-6’), 75.8 (C-2’) |
| 6’β | 3.76 (1H, dd, 12.5, 5.9) | 63.6 | H-6’α | 79.1 (C-3’) | 3.77 (1H, dd, 12.5, 5.9) | 63.5 | H-6’α | 78.7 (C-3’) |
| 6’α | 3.92 (1H, dd, 12.4, 2.2) | 63.6 | H-6’β | 72.4 (C-4’) | 3.94 (1H, dd, 12.4, 2.2) | 63.5 | H-6’β | 72.2 (C-4’) |

**Supplementary Table 4.** 1 & 2D-NMR data for miyaquinol **8** (D2O:CD_3_OD, 80:20 containing d_4_-TSP (0.01% w/v)) δ in ppm relative to d_4_-TSP at 0.00.

|  | D2O:CD_3_OD | | | |
| --- | --- | --- | --- | --- |
| Position | δ_H_ (ppm) | δ_C_ (ppm) | ^1^H-^1^H correlation to: | ^1^H-^13^C HMBC correlation to: |
| 1 | - | 157.6 | - |  |
| 2 | 7.25 (1H, d, 8.1) | 117.7 | H-3 | 125.8 (C-6),157.6 (C-1) |
| 3 | 7.43 (1H, m) | 133.5 | H-2 & H-4 | 157.6 (C-1), 133.5 (C-5) |
| 4 | 7.17 (1H, t, 7.5) | 125.9 | H-3 & H-5 | 117.7 (C-2), 125.8 (C-6) |
| 5 | 7.43 (1H, m) | 133.5 | H-4 | 157.6 (C-1), 133.5 (C-3) |
| 6 | - | 125.8 |  |  |
| 7α | 5.45 (1H, d, 12.2) | 66.7 | H-7β | 125.8 (C-6), 133.5 (C-5), 157.6 (C-1), 173.1 (C-8) |
| 7β | 5.26 (1H, d, 12.2) | 66.7 | H-7α | 125.8 (C-6), 133.5 (C-5), 157.6 (C-1), 173.1 (C-8) |
| 8 | - | 173.1 | - |  |
| 9 | - | 76.9 | - |  |
| 10 | - | 203.9 | - |  |
| 11 | 4.45 (1H, dd, 5.9, 1.4) | 59.2 | H-12 | 76.9 (C-9), 118.0 (C-17), 128.5 (C-15), 131.5 (C-16), 134.8 (C-12), 137.1 (C-13) |
| 12 | 6.66 (1H, ddd, 7.6, 6.0, 1.8) | 134.8 | H-13 & H-11 | 46.8 (C-14), 59.2 (C-11) |
| 13 | 6.55 (1H, ddd, 7.5, 6.3, 1.4) | 137.1 | H-12 & H-14 | 46.8 (C-14), 59.2 (C-11) |
| 14 | 4.79 (obscured by H_2_O) | 46.8 | H-13 | 76.9 (C-9), 128.5 (C-15), 131.5 (C-16), 134.8 (C-12), 137.1 (C-13) |
| 15 | - | 128.5 | - | - |
| 16 | - | 131.5 | - | - |
| 17 | 6.81 (1H, d, 8.0) | 118.0 | H-18 | 128.5 (C-15), 147.4 (C-19), 59.2 (C-11) |
| 18 | 6.79 (1H, d, 8.0) | 117.9 | H-17 | 131.5 (C-16), 144.5 (C-20) |
| 19 | - | 147.4 | - |  |
| 20 | - | 144.5 | - |  |
| 1’ | 5.14 (1H, d, 7.5) | 102.8 | H-2’ | 157.7 (C-1) |
| 2’ | 3.64 (1H, m) | 76.0 | H-1’ & H-3’ | n.d. |
| 3’ | 3.67-3.58 (1H, m) | 79.1 | H-2’ & H-,4’ | n.d. |
| 4’ | 3.52 (1H, m) | 72.6 | H-3’ & H-5’ | n.d. |
| 5’ | 3.62 (1H, m) | 79.1 | H-4’ & H-6’ | n.d. |
| 6’β | 3.73 (1H, dd, 12.4, 5.7) | 63.8 | H-6’α | n.d. |
| 6’α | 3.92 (1H, dd, 12.4, 2.2) | 63.8 | H-6’β | n.d. |

**Supplementary Table 5.** Concentrations of dimeric compounds and key salicinoids in juvenile leaf and stem tissue from 26 *Salix* species of the National Willow Collection held at Rothamsted Research. Concentrations are given in mg/g d.w.

| **Rres NWC Code** | **Tissue** | **Species** | **Clonal / Hybrid Name** | **Miyabeacin** | **Miyabeanol** | **Miyabeacin B** | **Salicortin** | **Salicin** |
| --- | --- | --- | --- | --- | --- | --- | --- | --- |
| 2 | Leaf | *S. nigra* Marsh. | SN3 Primrose Hill | n.d. | n.d. | n.d. | 56.26 | 45.41 |
| 15 | Leaf | *S. pentandra* L. | patent Lumley | n.d. | n.d. | n.d. | 5.88 | 5.07 |
| 207 | Leaf | *S. alba* L. | "Portogruaro (Ve)" | n.d. | n.d. | n.d. | 1.16 | 1.00 |
| 390 | Leaf | *S. fragilis* | R838 | n.d. | n.d. | n.d. | 1.33 | 0.66 |
| 415 | Leaf | *S. magnifica* Hemsl. | WB 50 0 578 | n.d. | n.d. | n.d. | 5.38 | 13.00 |
| 432 | Leaf | *S. daphnoides* Vill. | fastigiate | n.d. | n.d. | n.d. | 7.35 | n.d. |
| 506 | Leaf | *S. dasyclados* Wimm. | Grandis | 2.67 | 1.46 | 0.72 | n.d. | n.d. |
| 575 | Leaf | *S. dasyclados* Wimm. | (aquatica) Jyvaskyla V768 | 4.57 | 2.93 | 2.26 | 1.38 | 3.86 |
| 576 | Leaf | *S. dasyclados* Wimm. | (aquatica) Yesipaju Lieto V769 | 1.85 | 6.70 | 1.82 | 2.41 | 5.73 |
| 577 | Leaf | *S. dasyclados* Wimm. | 77056 IEA Trial | 45.25 | 16.20 | 8.41 | 19.10 | 12.20 |
| 592 | Leaf | *S. dasyclados* Wimm. | CE78-2 as x dasyclados Siren | 40.73 | 15.94 | 10.32 | 12.48 | 9.34 |
| 607 | Leaf | *S. rehderiana* Schneid. |  | n.d. | n.d. | n.d. | 57.58 | 2.98 |
| 608 | Leaf | *S. rehderiana* Schneid. |  | n.d. | n.d. | n.d. | 26.45 | 8.43 |
| 615 | Leaf | *S. schwerinii* Wolf | K3 Hilliers (WB 50 0 354 | n.d. | n.d. | n.d. | 0.70 | n.d. |
| 663 | Leaf | *S. viminalis* L. | Pulchra Ruberrima | n.d. | n.d. | n.d. | 1.15 | n.d. |
| 837 | Leaf | *S.miyabeana* Seemen | III | 79.86 | 55.01 | 15.45 | 1.24 | 37.29 |
| 838 | Leaf | *S. purpurea* L. | Richartii | n.d. | n.d. | n.d. | 44.91 | 68.22 |
| 844 | Leaf | *S. purpurea* L. | Uralensis | n.d. | n.d. | n.d. | 26.85 | 51.26 |
| 885 | Leaf | *S.miyabeana* Seemen | Shrubby | 79.11 | 37.96 | 16.09 | 5.39 | 41.82 |
| 901 | Leaf | *S.* × *alberti* L. (*S. integra* Thunb. × *S. suchowensis* W.C. Cheng ex G.Zhu) | 42/17 | n.d. | n.d. | n.d. | 6.39 | n.d. |
| 941 | Leaf | *S.miyabeana* Seemen | Purpurescens (ex.Tuinzing) (566) | 98.30 | 40.20 | 26.74 | 3.82 | 27.30 |
| 1013 | Leaf | *S.phylicifolia* L. | Malham | n.d. | n.d. | n.d. | 0.15 | n.d. |
| 1059 | Leaf | *S.repens* L. |  | 0.70 | 1.08 | 0.54 | 24.78 | 3.79 |
| 1155 | Leaf | *S. acutifolia* Willd. | 174 | n.d. | n.d. | n.d. | 6.90 | 165.40 |
| 1165 | Leaf | *S. arbusculoides* Anderss. | 20397 | n.d. | n.d. | n.d. | n.d. | n.d. |
| 1215 | Leaf | *S. myrsinifolia* Salisb. | E-4-1403 | n.d. | n.d. | n.d. | 8.55 | 105.85 |
| 2 | Stem | *S. nigra* Marsh. | SN3 Primrose Hill | n.d. | n.d. | n.d. | 81.11 | 42.52 |
| 15 | Stem | *S. pentandra* L. | patent Lumley | n.d. | n.d. | n.d. | 11.46 | 4.60 |
| 207 | Stem | *S. alba* L. | "Portogruaro (Ve)" | n.d. | n.d. | n.d. | 0.86 | 0.51 |
| 390 | Stem | *S. fragilis* | R838 | n.d. | n.d. | n.d. | 1.05 | n.d. |
| 415 | Stem | *S. magnifica* Hemsl. | WB 50 0 578 | n.d. | n.d. | n.d. | 9.90 | 30.36 |
| 432 | Stem | *S. daphnoides* Vill. | fastigiate | n.d. | n.d. | n.d. | 94.58 | 4.08 |
| 506 | Stem | *S. dasyclados* Wimm. | Grandis | 0.82 | 1.58 | 0.79 | 0.95 | n.d. |
| 575 | Stem | *S. dasyclados* Wimm. | (aquatica) Jyvaskyla V768 | 1.39 | n.d. | n.d. | 1.02 | n.d. |
| 576 | Stem | *S. dasyclados* Wimm. | (aquatica) Yesipaju Lieto V769 | 1.52 | n.d. | 0.69 | 2.07 | 1.06 |
| 577 | Stem | *S. dasyclados* Wimm. | 77056 IEA Trial | 14.86 | 3.28 | 3.73 | 6.60 | 5.09 |
| 592 | Stem | *S. dasyclados* Wimm. | CE78-2 as x dasyclados Siren | 13.14 | 5.82 | 4.88 | 2.81 | 5.67 |
| 607 | Stem | *S. rehderiana* Schneid. |  | n.d. | n.d. | n.d. | 9.84 | 18.37 |
| 608 | Stem | *S. rehderiana* Schneid. |  | n.d. | n.d. | n.d. | 23.38 | 17.31 |
| 608 | Stem | *S. rehderiana* Schneid. |  | n.d. | n.d. | n.d. | 7.44 | 23.27 |
| 615 | Stem | *S. schwerinii* Wolf | K3 Hilliers (WB 50 0 354 | n.d. | n.d. | n.d. | n.d. | n.d. |
| 628 | Stem | *S.viminalis* × *S.schwerinii* | Tora | n.d. | n.d. | n.d. | n.d. | n.d. |
| 663 | Stem | S. viminalis L. | Pulchra Ruberrima | n.d. | n.d. | n.d. | 1.41 | n.d. |
| 837 | Stem | *S.miyabeana* Seemen | III | 72.22 | n.d. | 15.56 | 53.79 | 22.28 |
| 838 | Stem | *S. purpurea* L. | Richartii | n.d. | n.d. | n.d. | 69.39 | 13.61 |
| 844 | Stem | *S. purpurea* L. | Uralensis | n.d. | n.d. | n.d. | 45.70 | 28.29 |
| 885 | Stem | *S.miyabeana* Seemen | Shrubby | 49.54 | n.d. | 8.39 | 55.45 | 45.98 |
| 901 | Stem | *S.* × *alberti* L. (*S. integra* Thunb. × *S. suchowensis* W.C. Cheng ex G.Zhu) | 42/17 | n.d. | n.d. | n.d. | 80.01 | 2.63 |
| 941 | Stem | *S.miyabeana* Seemen | Purpurescens (ex.Tuinzing) (566) | 81.13 | 1.95 | 18.79 | 5.42 | 26.94 |
| 1013 | Stem | *S.phylicifolia* L. | Malham | n.d. | n.d. | n.d. | 0.47 | 1.03 |
| 1059 | Stem | *S.repens* L. |  | n.d. | n.d. | n.d. | 36.68 | 2.26 |
| 1155 | Stem | *S. acutifolia* Willd. | 174 | n.d. | n.d. | n.d. | 276.02 | 19.46 |
| 1165 | Stem | *S. arbusculoides* Anderss. | 20397 | n.d. | n.d. | n.d. | 1.58 | n.d. |
| 1215 | Stem | *S. myrsinifolia* Salisb. | E-4-1403 | n.d. | n.d. | n.d. | 60.23 | 22.19 |

**Supplementary Table 6.** Varietal provenance of Terra Nova and Endurance biomass willows.

|  |  |  |
| --- | --- | --- |
| Variety | Terra Nova | Endurance |
| Female parent | ((*S. viminalis* 'Bowles Hybrid' × *S. triandra* 'Dark Newkind') 'LA940140') | *S. rehderiana* |
| Male parent | *S. miyabeana* 'Shrubby' | *S. dasyclados* '77056' |
| Breeder | European Willow Breeding Partnership | European Willow Breeding Partnership |
| Sex | Female | Female |
| Ploidy level (*x*) ^a^ | 3 | 5 |
| CPVO registration date ^b^ | 2005 | 2013 |
| Breeders code | LA9801132 | LA980442 |
|  |  |  |
|  |  |  |
|  |  |  |
|  |  |  |
|  |  |  |

^a^ Estimated ploidy level given in: Macalpine WJ, Shield IF, Trybush SO, Hayes C, Karp A (2008) Overcoming barriers to crossing in willow (*Salix* spp.) breeding. Aspects Appl Biol 90:173-180

^b^ Date granted plant breeders rights by the Community Plant Variety Office (CVPO)

**Supplementary Table 7.** 1 & 2D-NMR data for acetylmiyabeacin **9a/9b** (D2O:CD_3_OD, 80:20 containing d_4_-TSP (0.01% w/v)) δ in ppm relative to d_4_-TSP at 0.00.

| Position | 2′-*O*-Acetyl miyabeacin (**9a**) | | | 2′′-*O*-Acetyl miyabeacin (**9b**) | | | |  |
| --- | --- | --- | --- | --- | --- | --- | --- | --- |
|  | ^δ^ | *J*_H-H_ (Hz) | ^13^C | ^δ^ | *J*_H-H_ (Hz) | | ^13^C |  |
| 1 | - | - | 157.96 | - | - | 157.96 | | |
| 2 | 7.19 (d) | 9.3 | 117.86 | 7.19 (d) | 9.3 | 117.86 | | |
| 3, 26 | 7.41 (ddd) | 8.0, 7.7, 2.0 | 133.63 | 7.41 (ddd) | 8.0, 7.7, 2.0 | 133.63 | | |
| 4, 25 | 7.12 (t)/7.10 (t) | 7.5 | 125.89 | 7.12 (t)/7.10 (t) | 7.5 | 125.89 | | |
| 5,24 | 7.36-7.29 (m) | - | 133.45 | 7.36-7.29 (m) | - | 133.45 | | |
| 6 | - | - | 126.67 | - | - | 126.67 | | |
| 7α | 5.09 (d) | 12.1 | 66.90 | 5.39 (d) | 12.1 | 66.90 | | |
| 7β | 5.03 (d) | 12.1 | 66.90 | 5.17 (d) | 12.1 | 66.90 | | |
| 8 | - |  | 173.79 | - |  | 173.79 | | |
| 9 | - |  | 82.57 | - |  | 82.57 | | |
| 10 | 3.65 (m) | - | 40.57 | 3.62 (m) | - | 40.57 | | |
| 11 | 3.62 (m) | - | 43.89 | 3.55 (m) | - | 43.89 | | |
| 12 | 6.59 (dd) | 10.2, 4.1 | 152.57 | 6.59 (dd) | 10.2, 4.1 | 152.57 | | |
| 13 | 6.02 (dd) | 10.2, 1.5 | 130.97 | 6.02 (dd) | 10.2, 1.5 | 130.97 | | |
| 14 |  |  | 198.87 |  |  | 198.87 | | |
| 15 | 3.53 (m) |  | 45.27 | 3.53 (m) |  | 45.27 | | |
| 16 | 6.19 (t) | 7.9, 6.9, 1.0 | 135.70 | 6.19 (t) | 7.9, 6.9, 1.0 | 135.70 | | |
| 17 | 5.91 (ddd) | 7.9, 6.5, 1.4 | 132.86 | 5.91 (ddd) | 7.9, 6.5, 1.4 | 132.86 | | |
| 18 | 3.43 (m) |  | 54.45 | 3.43 (m) |  | 54.45 | | |
| 19 | - | - | 210.26 | - | - | 210.26 | | |
| 20 | - | - | 80.37 | - | - | 80.37 | | |
| 21 | - | - | 173.64 | - | - | 173.64 | | |
| 22β | 5.36 (d) | 12.2 | 67.02 | 5.10 (1H,d) | 12.2 | 67.02 | | |
| 22α | 5.15 (d) | 12.2 | 67.02 | 5.03 (d) | 12.2 | 67.02 | | |
| 23 | - |  | 126.25 | - |  | 126.25 | | |
| 27 | 7.19 (d) | 9.3 | 117.86 | 7.19 (d) | 9.3 | 117.86 | | |
| 28 | - |  | 157.88 | - |  | 157.88 | | |
| 1’ | 5.23 (d) | 8.0 | 101.37 | 5.06 (d) | 8.0 | 101.37 | | |
| 1’’ | 5.08 (d) | 7.5 | 103.02 | 5.22 (d) | 7.5 | 103.02 | | |
| 2’ | 5.00 / 4.97 (dd) | 9.6, 8.0 | 76.67 / 76.69 | 3.55-3.63 (m) | 9.6, 8.0 | 76.67 / 76.69 | | |
| 2’’ | 3.55-3.63 (m) | - | 76.08 | 5.00 / 4.97 (dd) | - | 76.08 | | |
| 3’ | 3.78 (m) | - | 76.73 | 3.58 (m) | - | 76.73 | | |
| 3’’ | 3.58 (m) | - | 76.16 | 3.78 (m) | - | 76.16 | | |
| 4’ | 3.58-3.64 (m) | - | 72.52 | 3.49(m) | - | 72.52 | | |
| 4’’ | 3.49(m) | - | 72.53 | 3.58-3.64 (m) | - | 72.53 | | |
| 5’ | 3.66 (m) | - | 79.20 | 3.58 (m) | - | 79.20 | | |
| 5’’ | 3.58 (m) | - | 78.99 | 3.66 (m) | - | 78.99 | | |
| 6’β | 3.77 (dd) | 12.4, 6.0 | 63.69 | 3.73 (dd) | 12.4, 6.0 | 63.69 | | |
| 6’’β | 3.73 (dd) | 12.4, 6.0 | 63.69 | 3.77 (dd) | 12.4, 6.0 | 63.69 | | |
| 6’α | 3.94 (dd) | 12.4, 2.1 | 63.69 | 3.92 (dd) | 12.4, 2.1 | 63.69 | | |
| 6’’α | 3.92 (dd) | 12.4, 2.1 | 63.69 | 3.94 (dd) | 12.4, 2.1 | 63.69 | | |
| 7’’ | - | - | 175.76/ 176.34 | - | - | 175.76/ 176.34 | | |
| 8’’ | 2.143 (s) / 2.137 (s) | - | 23.44 | 2.143 (s) / 2.137 (s) | - | 23.44 | | |

**Supplementary Table 8.** ^1^H-NMR data for diacetylmiyabeacin **10** (D2O:CD_3_OD, 80:20 containing d_4_-TSP (0.01% w/v)) δ in ppm relative to d_4_-TSP at 0.00.

| Position | 2′, 2′′-*O*-Diacetyl miyabeacin (**10**) | |
| --- | --- | --- |
|  | ^δ^ | *J*_H-H_ (Hz) |
| 1 | - | - |
| 2 | 7.19 (d) / 7.20 (d) | 8.4 |
| 3, 26 | 7.40 (ddd) /7.41 (ddd) | 8.0, 7.7, 2.0 |
| 4, 25 | 7.11 (t)/7.13 (t) | 7.6 |
| 5,24 | 7.36-7.29 (m) | - |
| 6 | - | - |
| 7α, 22α | 5.12 (d) / 5.11 (d) | 11.7 / 12.1 |
| 7β, 22β | 5.05 (d) | 12.0 |
| 8 | - |  |
| 9 | - |  |
| 10 | 3.51 – 3.70 (m) | - |
| 11 | 3.51 – 3.70 (m) | - |
| 12 | 6.62 (dd) | 10, 4.4 |
| 13 | 6.03 (dd) | 10.1, 1.4 |
| 14 |  |  |
| 15 | 3.51 – 3.70 (m) |  |
| 16 | 6.22 (t) | 6.7 |
| 17 | 5.93 (ddd) | 7.9, 6.5, 1.4 |
| 18 | 3.51 – 3.70 (m) |  |
| 19 | - | - |
| 20 | - | - |
| 21 | - | - |
| 23 | - |  |
| 27 | 7.19 (d) / 7.20 (d) | 8.4 |
| 28 | - |  |
| 1’, 1’’ | 2 x 5.24 (d) | 7.9 |
| 2’, 2’’ | 5.01 (dd) / 4.98 (dd) | 9.6, 8.0 |
| 3’, 3’’ | 3.79 (m) | - |
| 4’, 4’’ | 3.51 – 3.70 (m) | - |
| 5’, 5’’ | 3.51 – 3.70 (m) | - |
| 6’β, 6’’β | 3.77 (dd), 3.82 (dd) | 12.4, 6.0 |
| 6’α | 3.98 (dd) | 12.4, 2.1 |
| 6’’α | 3.96 (dd) | 12.4, 2.1 |
| 7’, 7’’ | - | - |
| 8’, 8’’ | 2 x 2.16 (s) | - |

**Supplementary Table 9.** Extraction and HPLC gradient conditions for the isolation of dimeric metabolites.

| **Compound Number** | **Compound Name** | **Amount Extracted** | **Tissue** | **Extraction Volume (solvent: H_2_O:MeOH** | **Number of 100 µL Injections made into HPLC** | **HPLC Gradient [mobile phases** **water (A) and acetonitrile (B), both containing 0.1% formic acid.]** | **HPLC Retention time of Peak** | **Amount Isolated** |
| --- | --- | --- | --- | --- | --- | --- | --- | --- |
| **3** | Miyabeacin | 50 mg | *Salix miyabeana* Seemen. III leaf tissue. Line: NWC837 | 1 mL | 6 | 5% B (0-10 min), 22% B (10-50 min) to 37 % B (60-70 min). | 57.93 min | 1.68 mg |
| **6** | Miyabeacin B | 200 mg | *Salix miyabeana* Seemen. “Purpurescens” stem tissue. Line: NWC941 | 2.5 mL | > 10 | 5% B (0-10 min), 29% B (10-60 min) to 29 % B (60-70 min) | 52.11 min | 0.67 mg |
| **7** | Miyabeanol | 150 mg | *Salix miyabeana* Seemen. III leaf tissue. Line: NWC837 | 2 mL | 8 | 5% B (0-10 min), 22% B (10-50 min) to 37 % B (60-70 min) | 44.87 min | 1.05 mg |
| **8** | Miyaquinol | 450 mg | *Salix miyabeana* Seemen. “Purpurescens” leaf tissue. Line: NWC941 | 4.5 mL | 44 | 20% B (0-20 min), 40% B (20-25 min) to 50 % B (25-35 min) | 20.9 min | 0.9 mg |
| **9a/9b** | 2′/2′′Acetyl miyabeacin | 150 mg (2 x 75 mg) | RRes 710-27, RR09102 hybrid [NWC607 S. rehderiana × RR05337 (Aud × S. rossica)] leaf tissue | 2.4 mL (2 1.2 mL) | 10 | 20% B (0 min), 40% B (0 – 45 min) to 100 % B (45.0-50 min) | 41.4 | 0.75 mg |
| **10** | 2′, 2′′ Diacetyl miyabeacin | 150 mg (2 x 75 mg) | RRes 710-27, RR09102 hybrid [NWC607 S. rehderiana × RR05337 (Aud × S. rossica)] leaf tissue | 2.4 mL (2 1.2 mL) | 10 | 20% B (0 min), 40% B (0 – 45 min) to 100 % B (45.0-50 min) | 45.5 | 0.25 mg |

**Supplementary Table 10.** General Conditions and typical parameters for NMR and Mass Spectral data collection.

| Measurement Conditions | |
| --- | --- |
| **High resolution LC-MS** |  |
| *LC apparatus* | Ultimate 3000 RS uHPLC (Thermo) |
| Chromatography Column | C_18_ Hypersil gold column (1.9 µm, 30 x 2.1 mm i.d.) |
| Column Temperature | 35°C |
| Solvents | Water/0.1% formic acid (A) and acetonitrile/0.1% formic acid (B) |
| Solvent Gradient | 0 min, 0 % B; 27 min, 70 % B; 28 min, 100% B. |
| Flow rate | 0.3 mL/min |
| Run time | 30 min |
| Injection volume | 10µL |
|  |  |
| *MS Apparatus* | LTQ-Orbitrap Elite (Thermo) |
| Source | Heated ESI source |
| Ionisation mode | Negative |
| Resolution | 120,000 |
| Capillary temperature | 350°C |
| Source heater temperature | 350°C |
| Source voltage | 2500 V |
| Source current | 100 uA |
| Sheath gas flow | 35 |
| Auxillary gas | 10 |
| R.F. Lens | 50% |
| Scan range | m/z 50-1500 |
| MS-MS fragmentation | Automatic on top 3 ions |
| Ion isolation width for MSMS | m/z 2 |
| Fragmentation mode | HCD |
| Normalised collision energy | 65 |
| Activation time | 0.1 ms |
|  |  |
| **NMR** |  |
| Apparatus | Avance 600 (Bruker) |
| Observation Frequency | ^1^H: 600.05, ^13^C: 150.9 |
| Solvent | D_2_O:CD_3_OD (80:20) |
| Concentration | 0.6 mg/mL |
| Internal Standard | d_4_-TSP |
| Temperature  Probe | 300K  5mm Selective Inverse |
|  |  |
| **^1^H NMR Measurement** |  |
| Pulse sequence  Sweep width | zgpr  7183 Hz |
| Spectrum offset  Data points | 2879.40 Hz  32,768 |
| Pulse angle | 90° |
| Delay | 5 s |
| Number of scans | 64 |
|  |  |
|  |  |
| **2D COSY 45 Measurement** |  |
| Pulse program  Observation width | cosyqf45  2973, 2973 Hz |
| Data points | 1024, 1024 |
| Temperature | 300K |
| Number of transients | 32 |
|  |  |
| **2D HSQC Measurement** |  |
| Pulse program  Observation width | hsqcetgpsi2  7180, 30150 Hz |
| Data points | 2048, 1024 |
| Temperature | 300K |
| Number of transients | 128 |
|  |  |
| **2D HMBC Measurement** |  |
| Pulse program  Observation width | hmbcgpndqf  7182, 33165 Hz |
| Data points | 4096, 256 |
| Temperature | 300K |
| Number of transients | 256 |
|  |  |
| **^13^C NMR** |  |
| Apparatus | Avance 400 (Bruker) |
| Observation Frequency | ^13^C: 100.61 |
| Solvent | D_2_O:CD_3_OD (80:20) |
| Concentration | 0.6 mg/mL |
| Internal Standard | d_4_-TSP |
| Temperature  Probe | 300K  5mm Broadband BBO |
|  |  |
|  |  |
| **^13^C NMR Measurement** |  |
| Pulse sequence  Sweep width | dept135  23,980 Hz |
| Spectrum offset  Data points | 10363 Hz  32768 |
| Pulse angle | 30° |
| Delay | 0.7 s |
| Number of scans | 46,191 |
|  |  |
| **DEPT Measurement** |  |
| Observation width | 23980 Hz |
| Data points | 65536 |
| Pulse repetition time | 2 |
| Number of scans | 4096 |
|  |  |

Abbreviations

DEPT: Distortionless Enhancement by Polarization Transfer (A method for determining a carbon type (distinguishing among CH3, CH2, CH, and C))

COSY: COrrelation SpectroscopY (A method of ^1^H-^1^H COSY)

HSQC: Heteronuclear Single Quantum Coherence (A method of ^1^H-^13^C COSY)

HMBC: Heteronuclear Multiple Bond Correlation (A method of long-ran


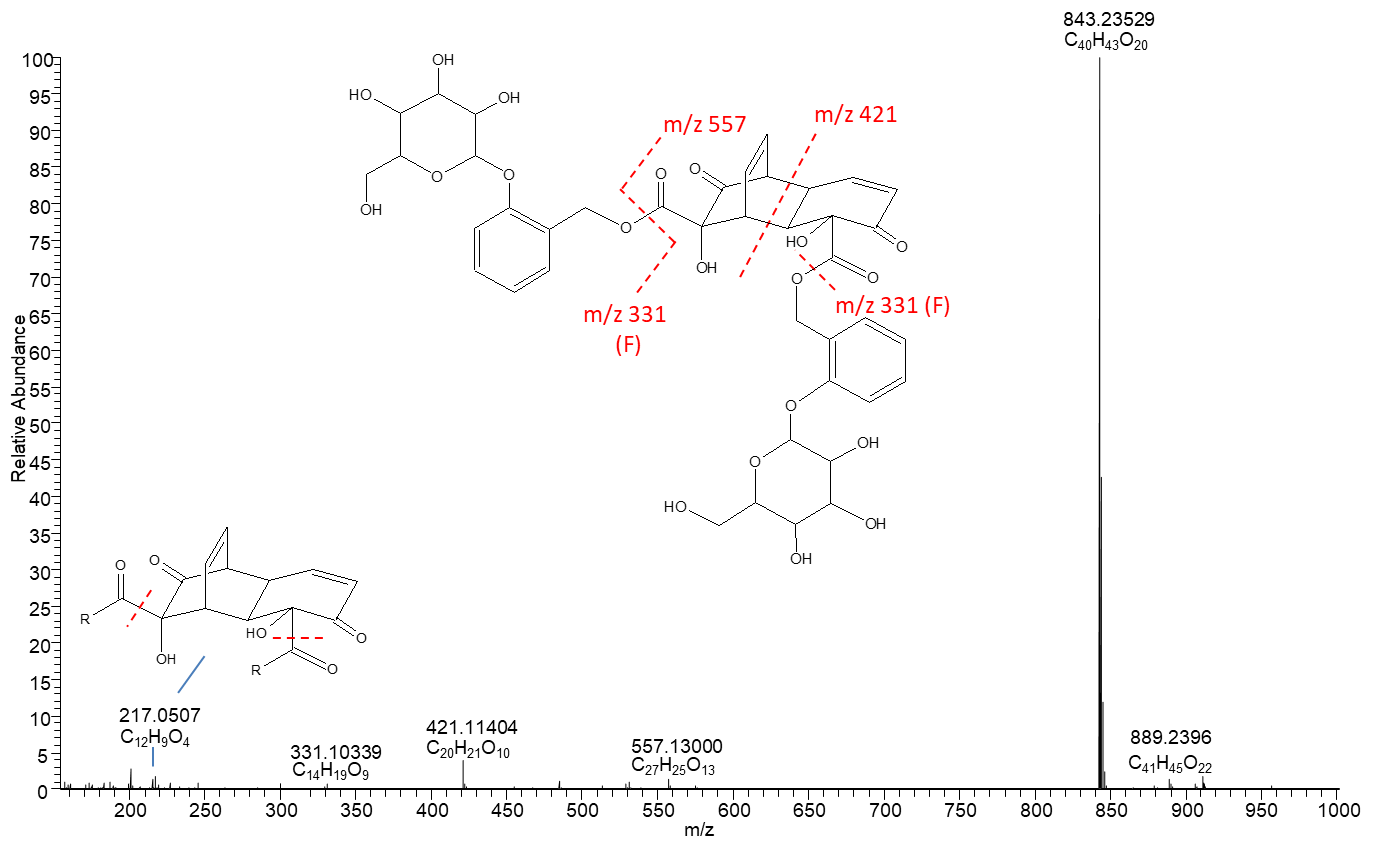


**Supplementary Figure 1.** Mass spectrum of miyabeacin **3** at m/z 843.2353 with retention time 25.26 min. Dashed lines show in-source fragmentation of the molecule. (F) indicates formate adduct


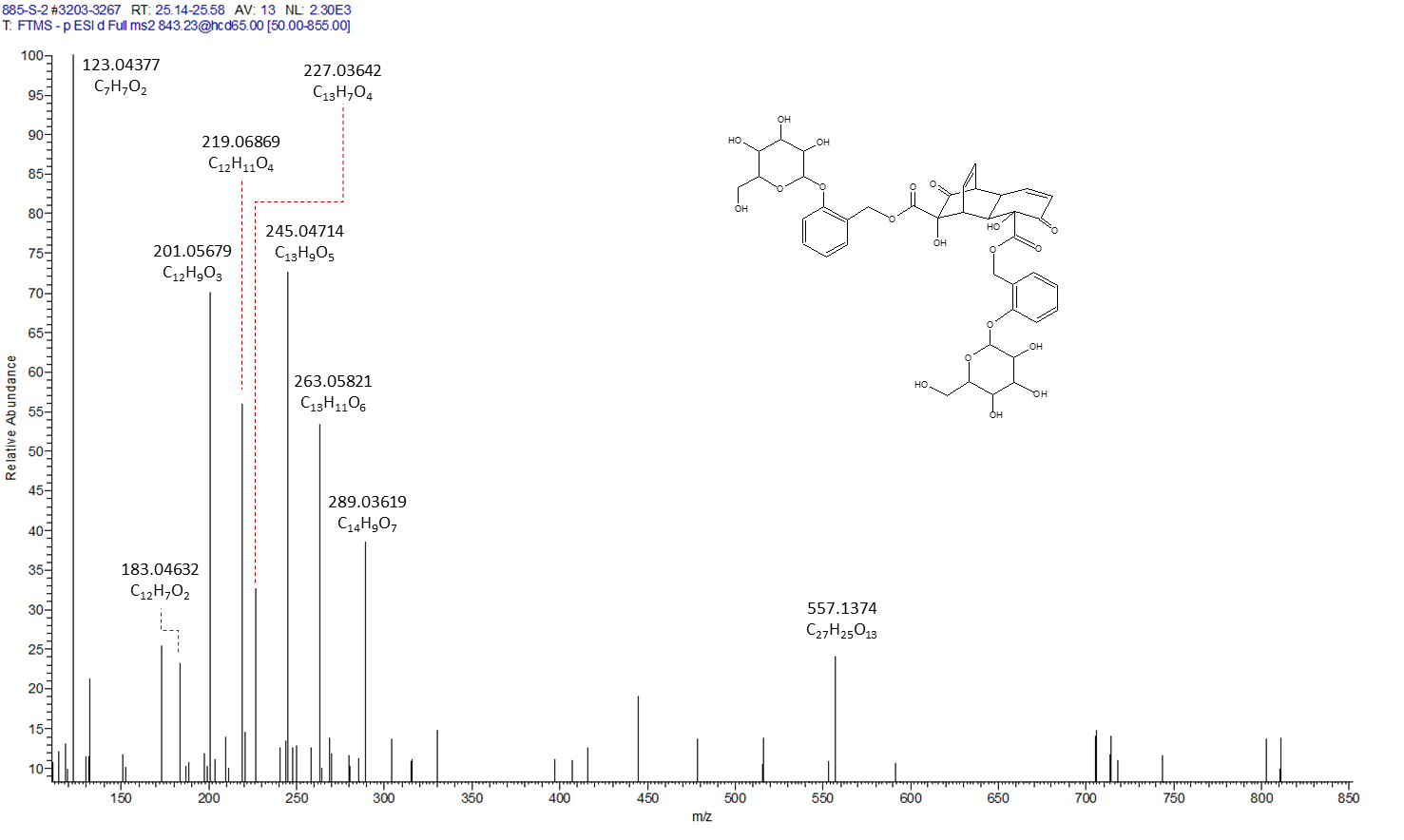


**Supplementary Figure 2.** MSMS data (negative ion mode) of m/z 843 ion of miyabeacin **3**


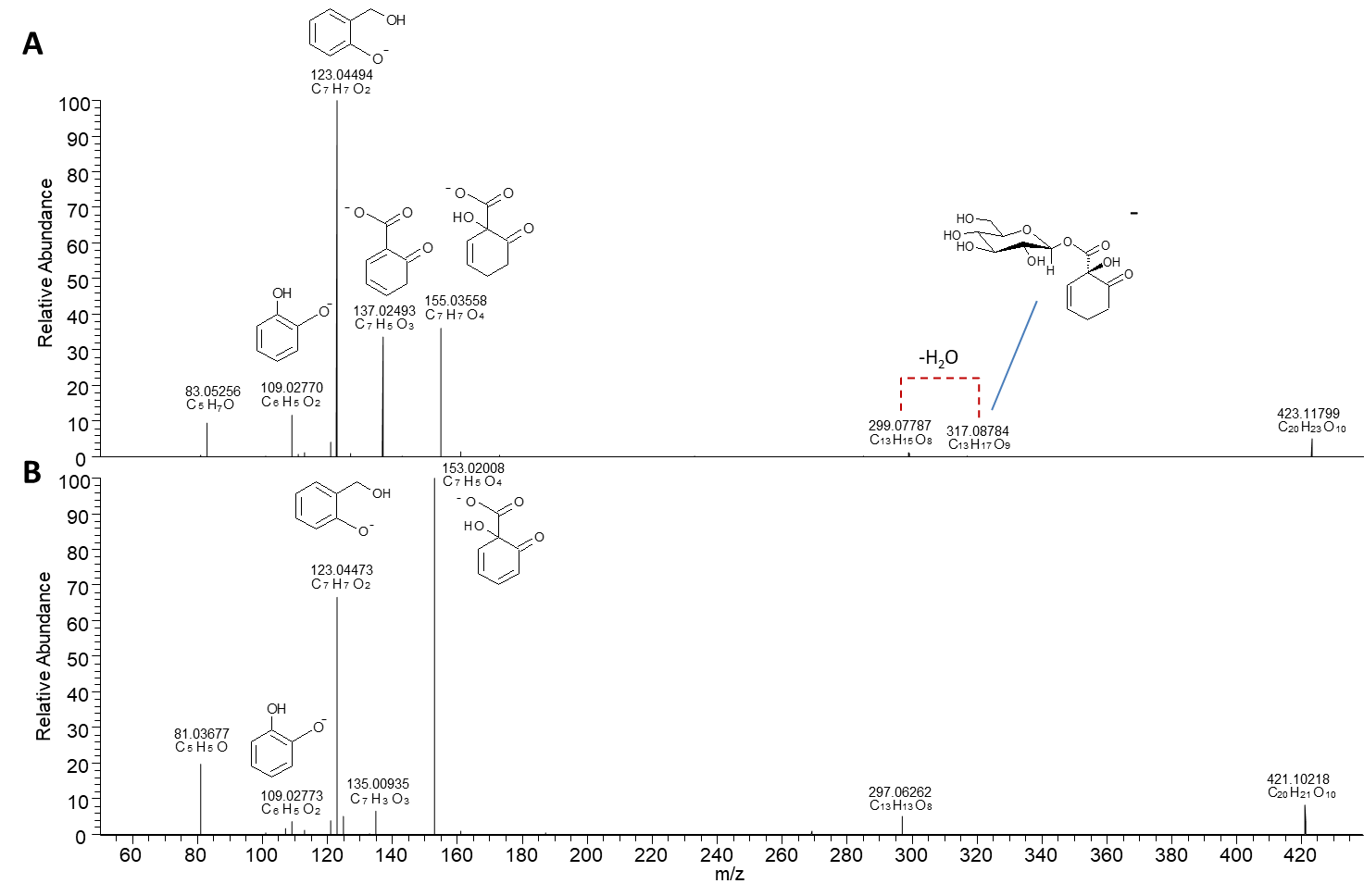


**Supplementary Figure 3.** MSMS comparison of A: salicortin **2** m/z 423 and B: miyabeacin **3** fragment (m/z 421). The structures for the ion at m/z 317 is believed to have arisen from a rearrangement following a neutral loss of orthoquinone methide from salicinoid structures.


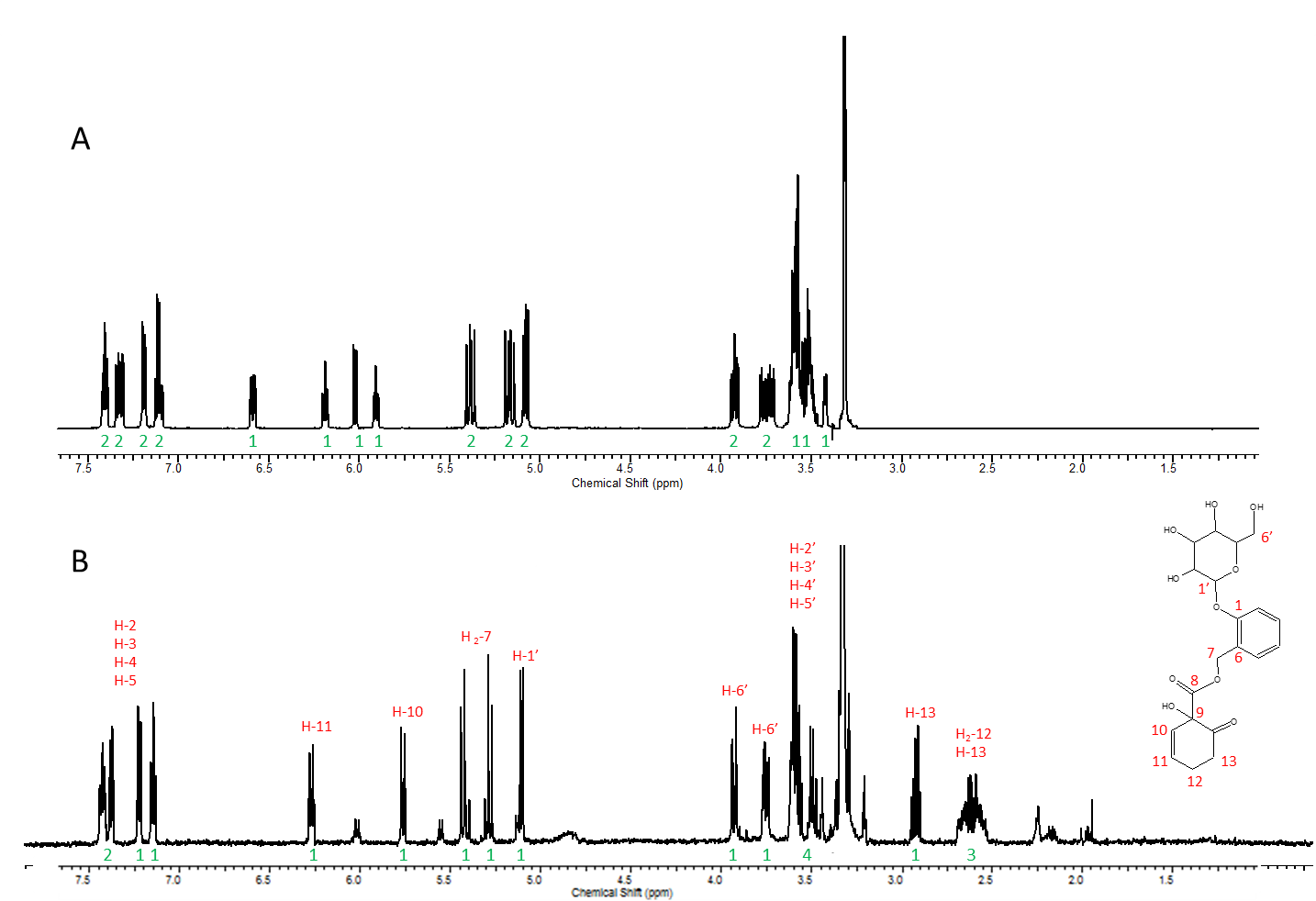


**Supplementary Figure 4.** Comparison of 600 MHz ^1^H-NMR spectra of A: miyabeacin **3** and B: salicortin **2** collected in 80:20 D_2_O:CD_3_OD containing 0.01 % w/v d_4_-TSP as reference standard. Numbers in red relate to structural assignment of salicortin. Numbers in green relate to peak integral values.

**Supplementary Figure 5.** COSY45 spectrum of miyabeacin **3**, collected at 600MHz in D_2_O:CD_3_OD (80:20)

**Supplementary Figure 6.** ^13^C spectrum of miyabeacin **3** collected at 400 MHz in D_2_O:CD_3_OD (80:20) containing 0.01% w/v d_4_TSP. Spectrum referenced to d_4_-TSP at δ0.00

**Supplementary Figure 7.** DEPT135 spectrum of miyabeacin **3**, collected at 100.6128 MHz in D_2_O:CD_3_OD (80:20)

**Supplementary Figure 8.** HSQC spectrum of miyabeacin **3** collected in D_2_O:CD_3_OD (80:20)

**Supplementary Figure 9.** HMBC spectrum of miyabeacin **3** collected in D_2_O:CD_3_OD (80:20)


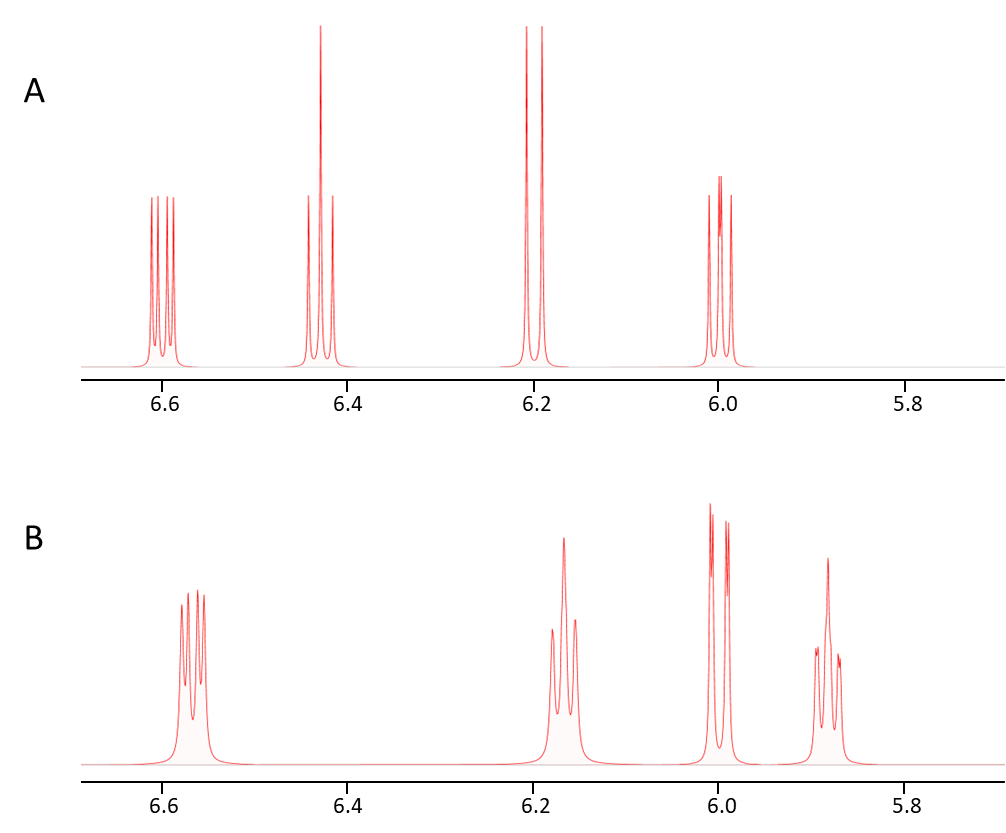


**Supplementary Figure 10.** Chenomx simulated ^1^H NMR spectrum (500 MHz) of grandifloracin in CDCl3 (δ 6.66 – 5.70) from data provided in Palframan *et al.,* 2011. B: ^1^H NMR spectrum (δ 6.66 – 5.70) of miyabeacin **3** in D_2_O:CD_3_OD (8:2) collected at 600 MHz.


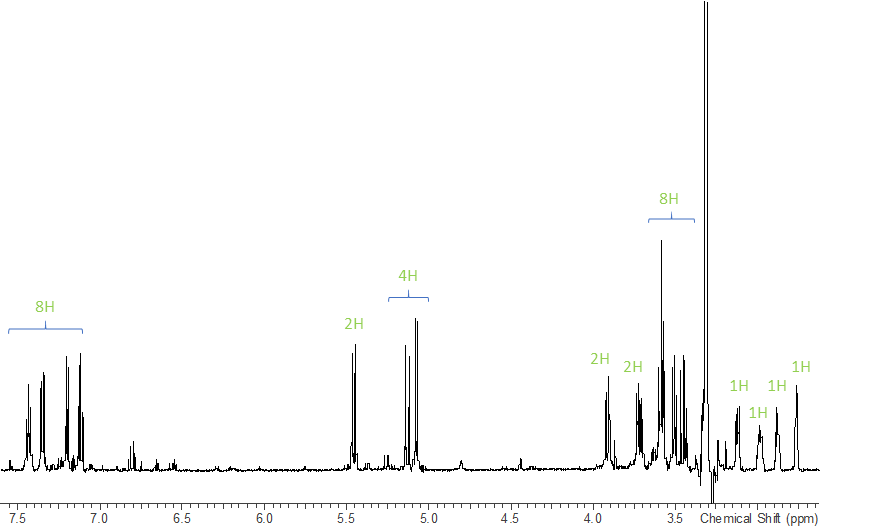


**Supplementary Figure 11.** 600 MHz ^1^H-NMR spectra of A: miyabeacin B **6** collected in D_2_O:CD_3_OD containing 0.01 % w/v d_4_-TSP as reference standard. Numbers in green relate to peak integral values.


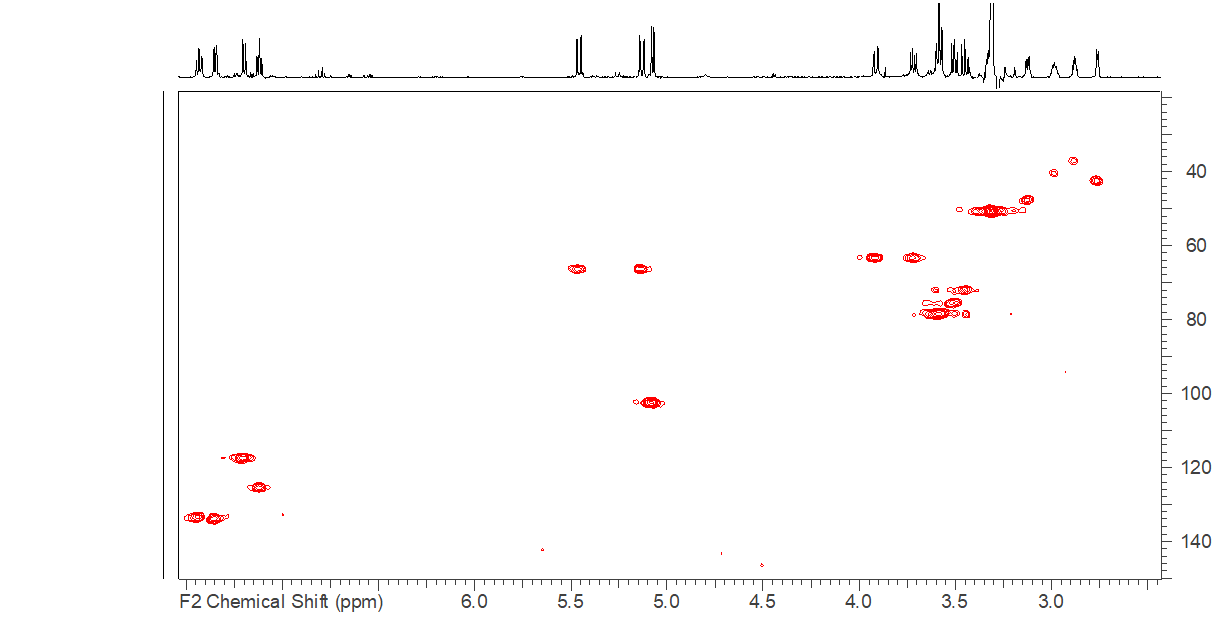


**Supplementary Figure 12.** HSQC spectrum of miyabeacin B **6** collected in D_2_O:CD_3_OD containing 0.01 % w/v d_4_-TSP as reference standard.


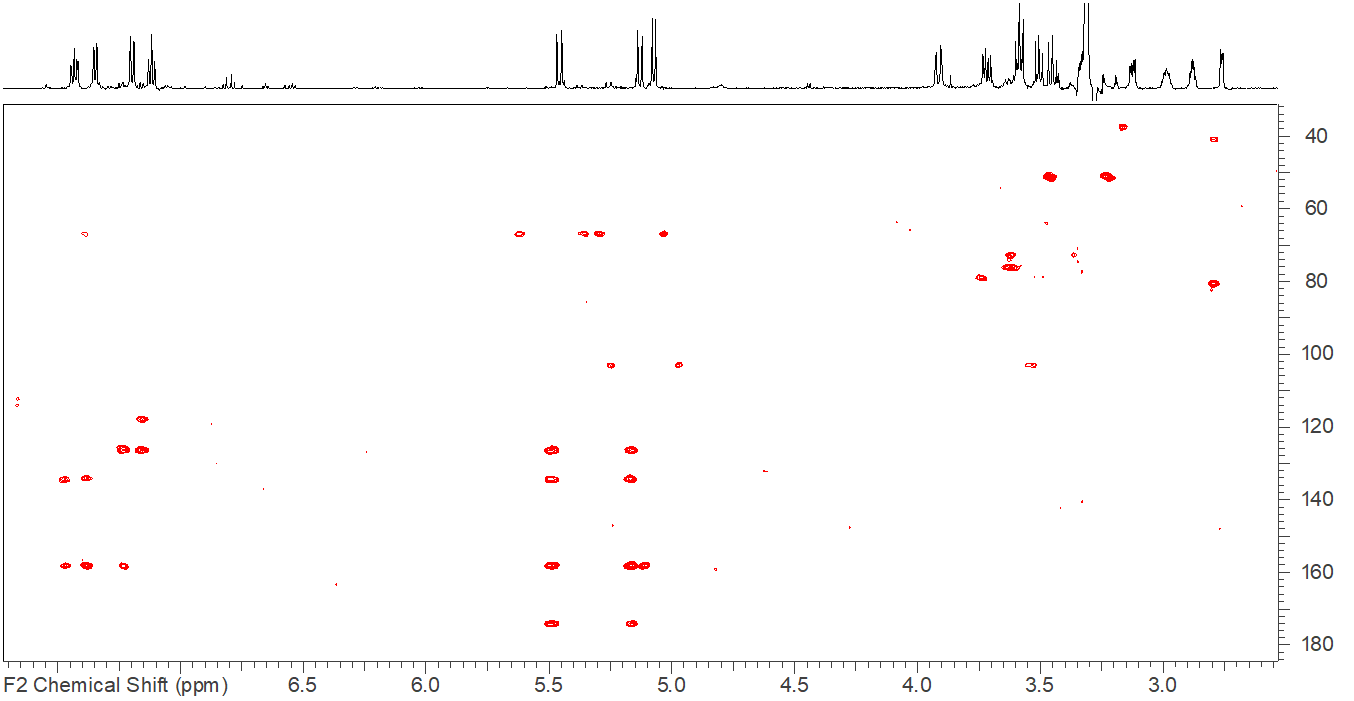


**Supplementary Figure 13.** HMBC spectrum of miyabeacin B **6** collected in D_2_O:CD_3_OD containing 0.01 % w/v d_4_-TSP as reference standard.


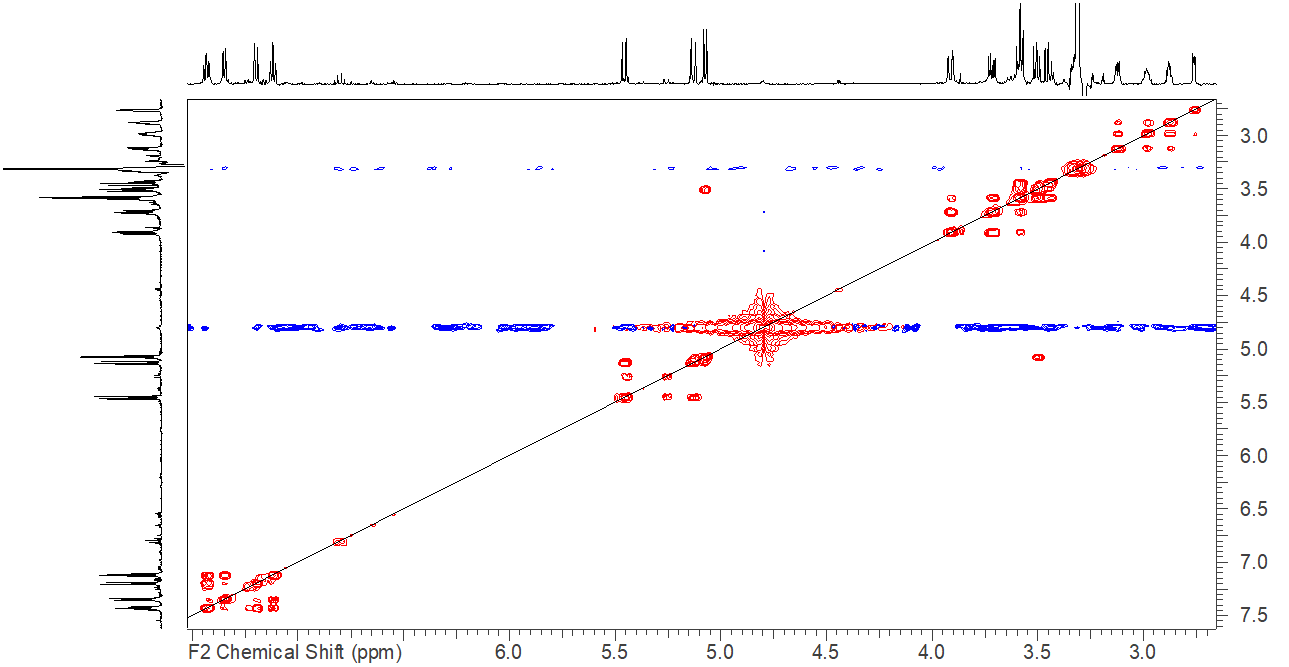


**Supplementary Figure 14.** COSY spectrum of miyabeacin B **6** collected in D_2_O:CD_3_OD containing 0.01 % w/v d_4_-TSP as reference standard.


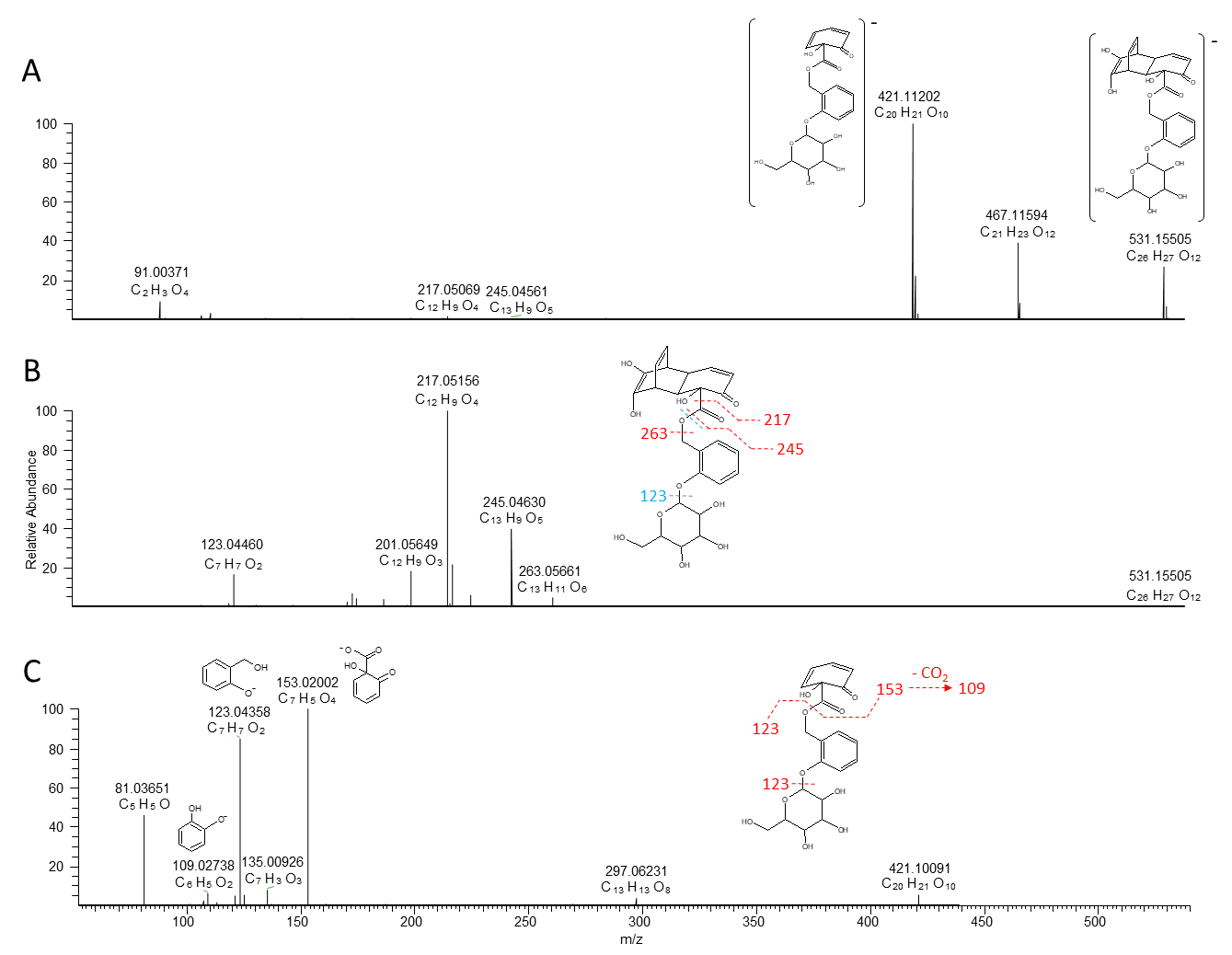


**Supplementary Figure 15.** MS and MSMS data of miyabeanol **7**. A: MS spectrum of peak at 20.13 min; B: MSMS of m/z 531 [M-H]^-^ ; C: MSMS of m/z 421 ([M-H]^-^ of retro Diels-Alder product)


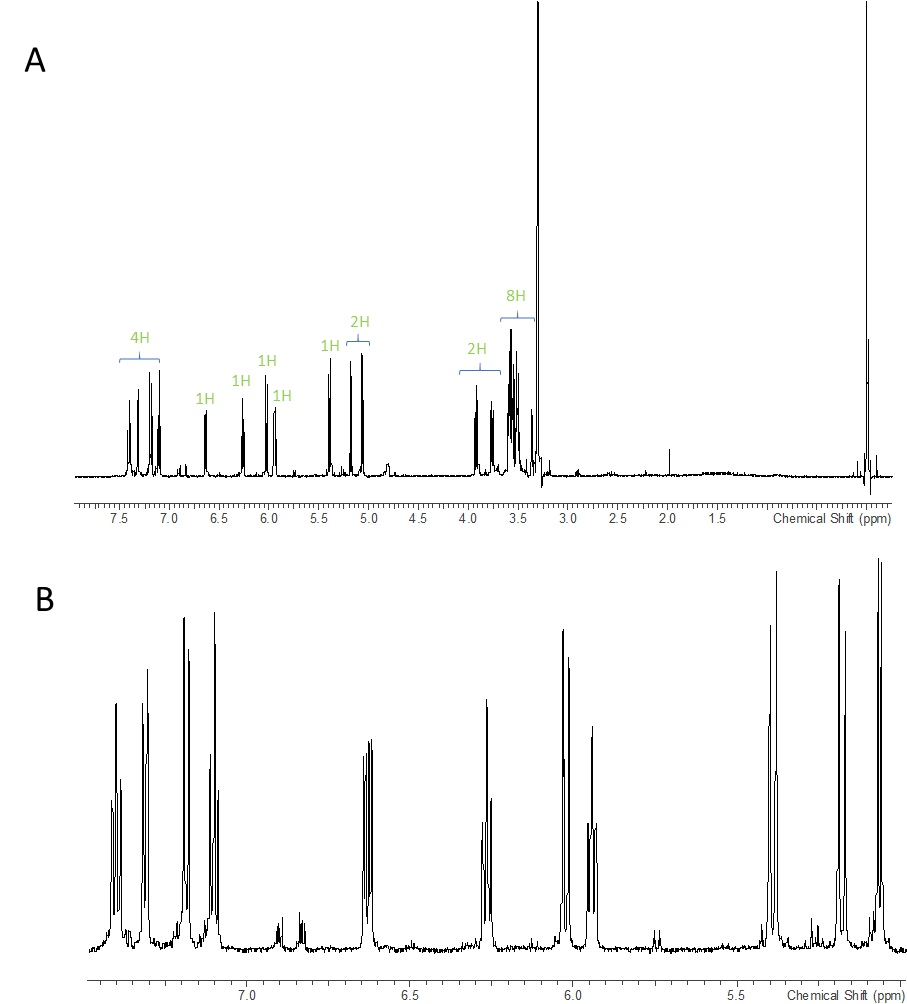


**Supplementary Figure 16.**  600 MHz ^1^H-NMR spectra of A: miyabeanol **7** collected in D_2_O:CD_3_OD containing 0.01 % w/v d_4_-TSP as reference standard. Numbers in green relate to peak integral values. and B: expansion of the region between δ7.50 – 5.0.

**Supplementary Figure 17.**  COSY45 spectrum of A: miyabeanol **7** collected in D_2_O:CD_3_OD containing 0.01 % w/v d_4_-TSP as reference standard.


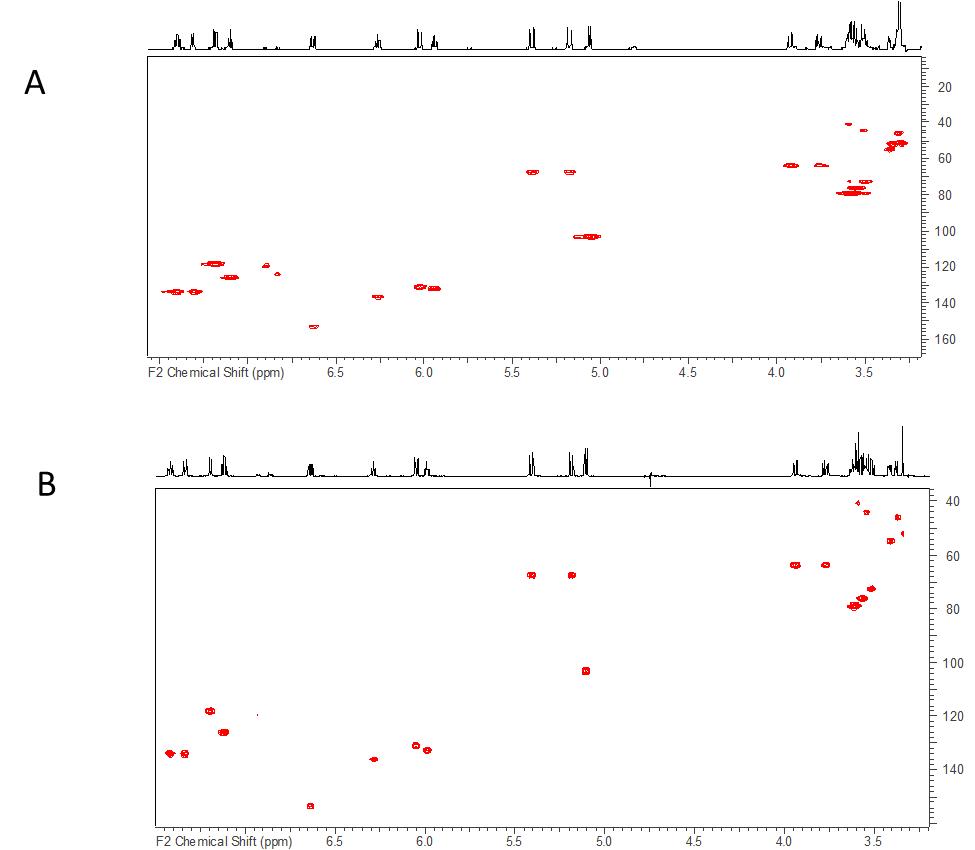


**Supplementary Figure 18.**  HSQC spectra of miyabeanol **7.** A: D_2_O:CD_3_OD and B: D_2_O


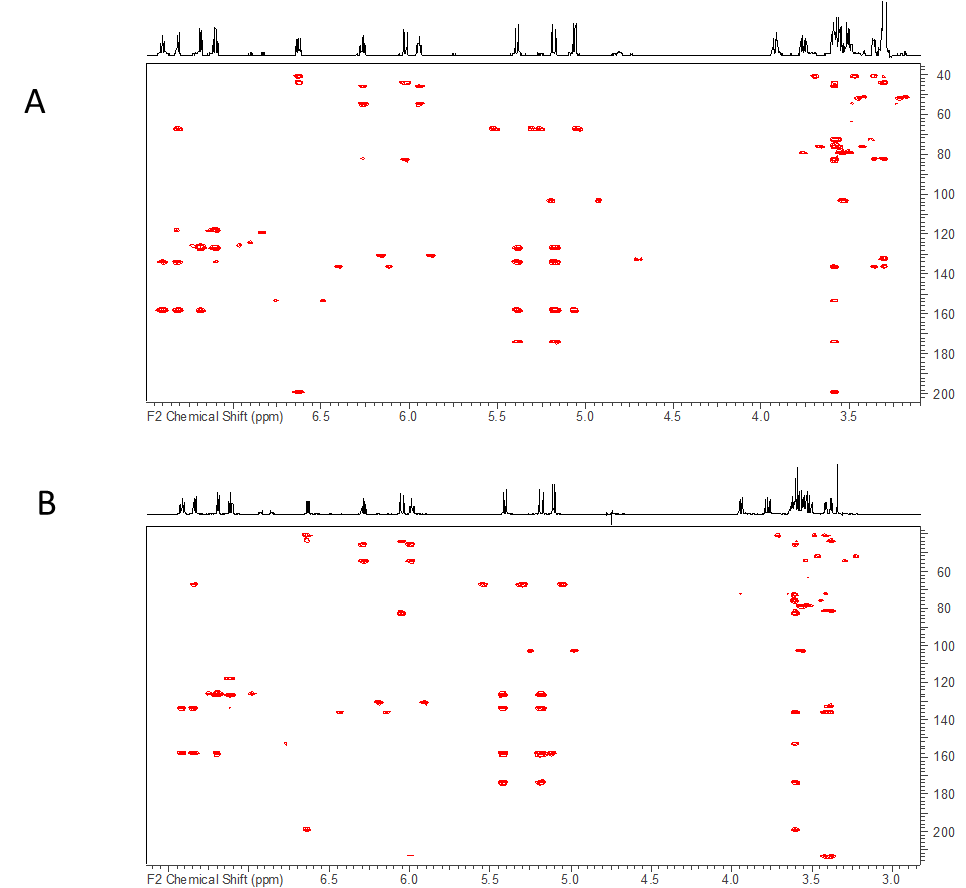


**Supplementary Figure 19.**  HMBC spectra of miyabeanol **7.** A: D_2_O:CD_3_OD and B: D_2_O


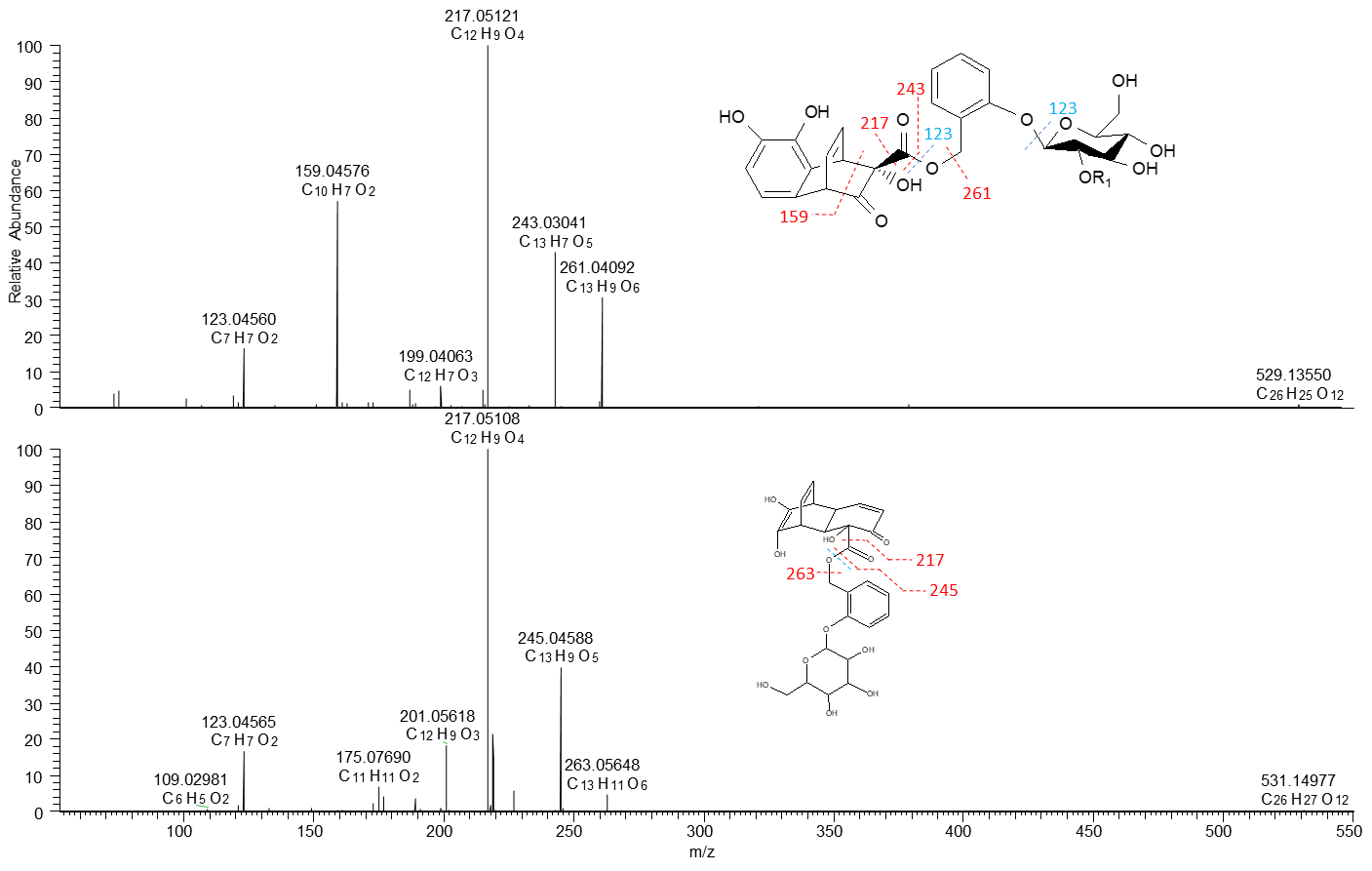


**Supplementary Figure 20.** MSMS data of miyaquinol **8**. A: MSMS spectrum of m/z 529 peak at 23.67 min; B: MSMS of m/z 531 [M-H]^-^ for comparison.

**Supplementary Figure 21.**  600 MHz ^1^H-NMR spectra of miyaquinol **8** collected in D_2_O:CD_3_OD containing 0.01 % w/v d_4_-TSP as reference standard.

**Supplementary Figure 22.**  HSQC spectra of miyaquinol **8** collected in D_2_O:CD_3_OD (80:20)

**Supplementary Figure 23.**  HMBC spectra of miyaquinol **8** collected in D_2_O:CD_3_OD (80:20)

**Supplementary Figure 24.**  Pearson correlations of compound concentrations **3**, **6** and **7.** A-C: Leaf concentrations; D: Stem concentrations.

A:

|  |  |  | uHPLC-MS Peak areas | | |
| --- | --- | --- | --- | --- | --- |
| NWC Code | Species | Variety | **7** | **8** |  |
| 941 | *S.miyabeana* Seemen | Purpurescens (ex.Tuinzing) (566) | 106,736,382 | 13,802,140 |  |
| 885 | *S.miyabeana* Seemen | Shrubby | 103,197,646 | 10,715,048 |  |
| 837 | *S.miyabeana* Seemen | III | 121,088,485 | 14,165,161 |  |
| 592 | *S. dasyclados* Wimm. | CE78-2 as x dasyclados Siren | 63,407,656 | 9,061,956 |  |
| 576 | *S. dasyclados* Wimm. | (aquatica) Yesipaju Lieto V769 | 6,688,741 | 790,941 |  |
| 575 | *S. dasyclados* Wimm. | (aquatica) Jyvaskyla V768 | 10,851,853 | 1,498,029 |  |
| 577 | *S. dasyclados* Wimm. | 77056 IEA Trial | 52,624,720 | 7,162,216 |  |

B:

**Supplementary Figure 25.**  Correlation of uHPLC-MS peak areas for **7** and **8** in *S.* *miyabeana* and *S.* *dasyclados* accessions.


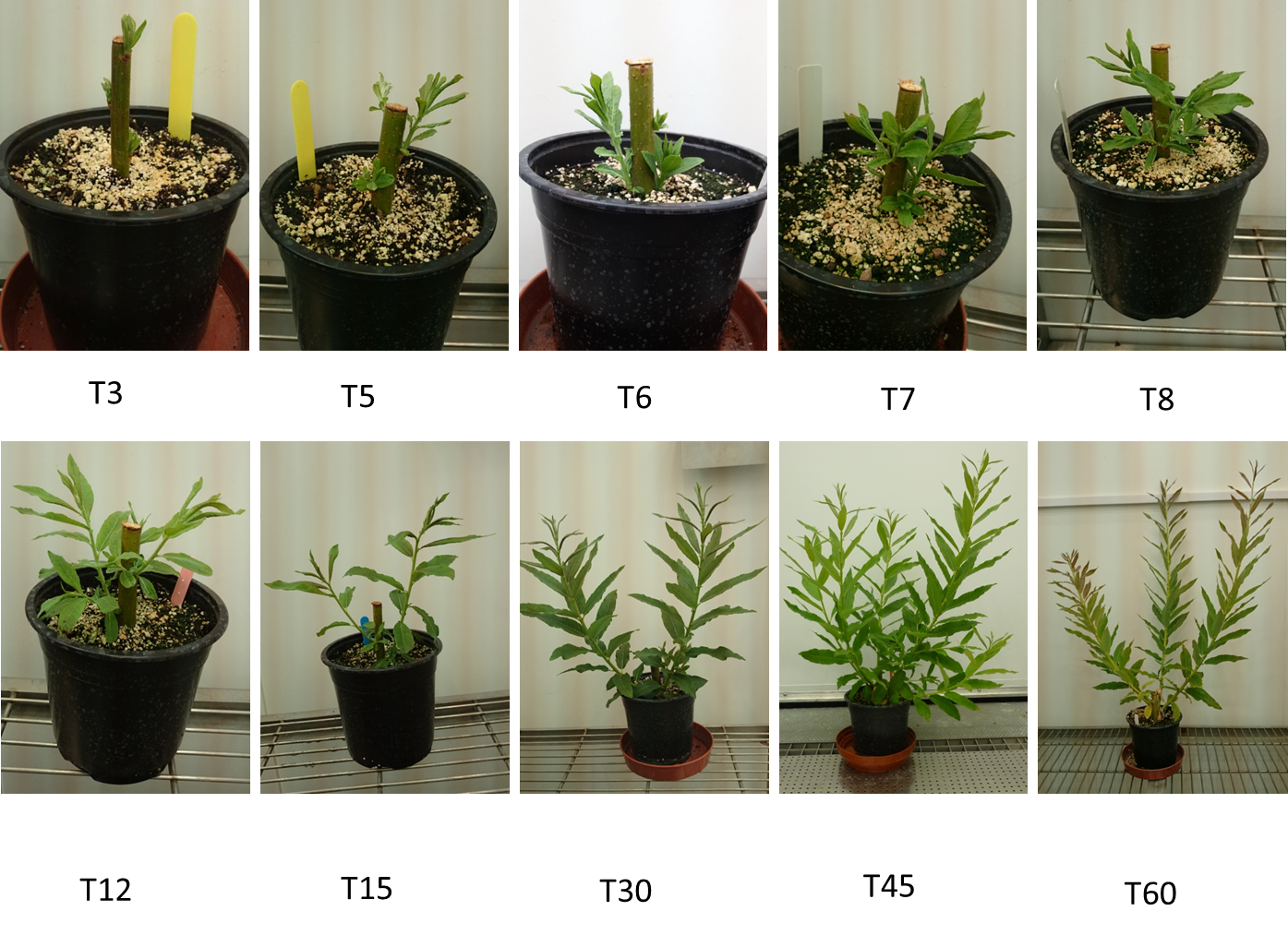


**Supplementary Figure 26.**  Photographs of *S.* *dasyclados* (NWC577) grown in controlled environment conditions. Photographs show typical sampled plants from 3 days after budburst (T3) to 60 days after bud burst (T60).


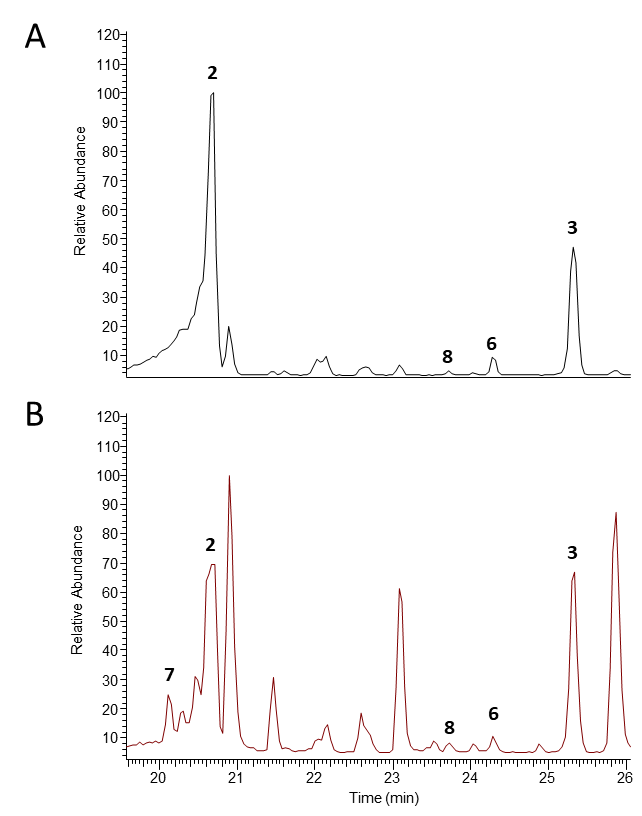


**Supplementary Figure 27.**  Total ion chromatograms (RT 19.6 – 26.0 min) from uHPLC-MS analyses of polar solvent extracts of Terra Nova (NWC1110). A: Juvenile stem; B: Juvenile leaf.


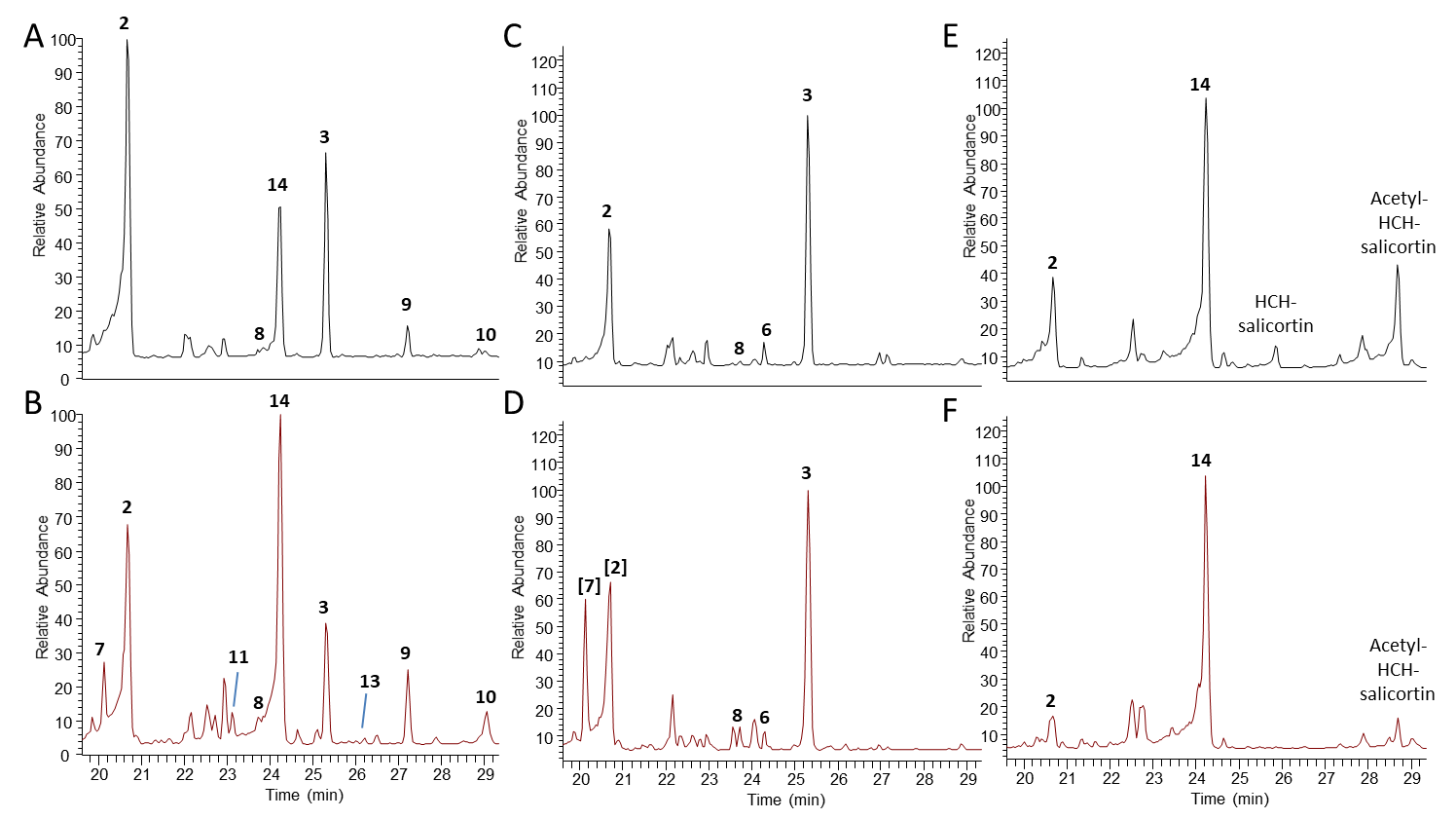


**Supplementary Figure 28.** Total ion chromatograms (RT 19.6 – 26.0 min) from uHPLC-MS analyses of polar solvent extracts of juvenile willow tissues. A: Endurance stem; B: Endurance leaf; C: *S. dasyclados* (NWC577) stem; D: *S. dasyclados* (NWC 577) leaf; E: *S. rehderiana* (NWC607) stem; F: *S. rehderiana* (NWC607) leaf. Peak labelling reflects labelling in the main text: **2**:salicortin, **3**:miyabeacin, **6**:miyabeacin-B, **7**:miyabeanol, **8**:miyaquinol, **9**:acetylmiyabeacin, **10**:diacetylmiyabeacin, **11**:2′-acetylmiyabinol, **13**:2′-acetylmiyaquinol, **14**: 2′-acetylsalicortin.


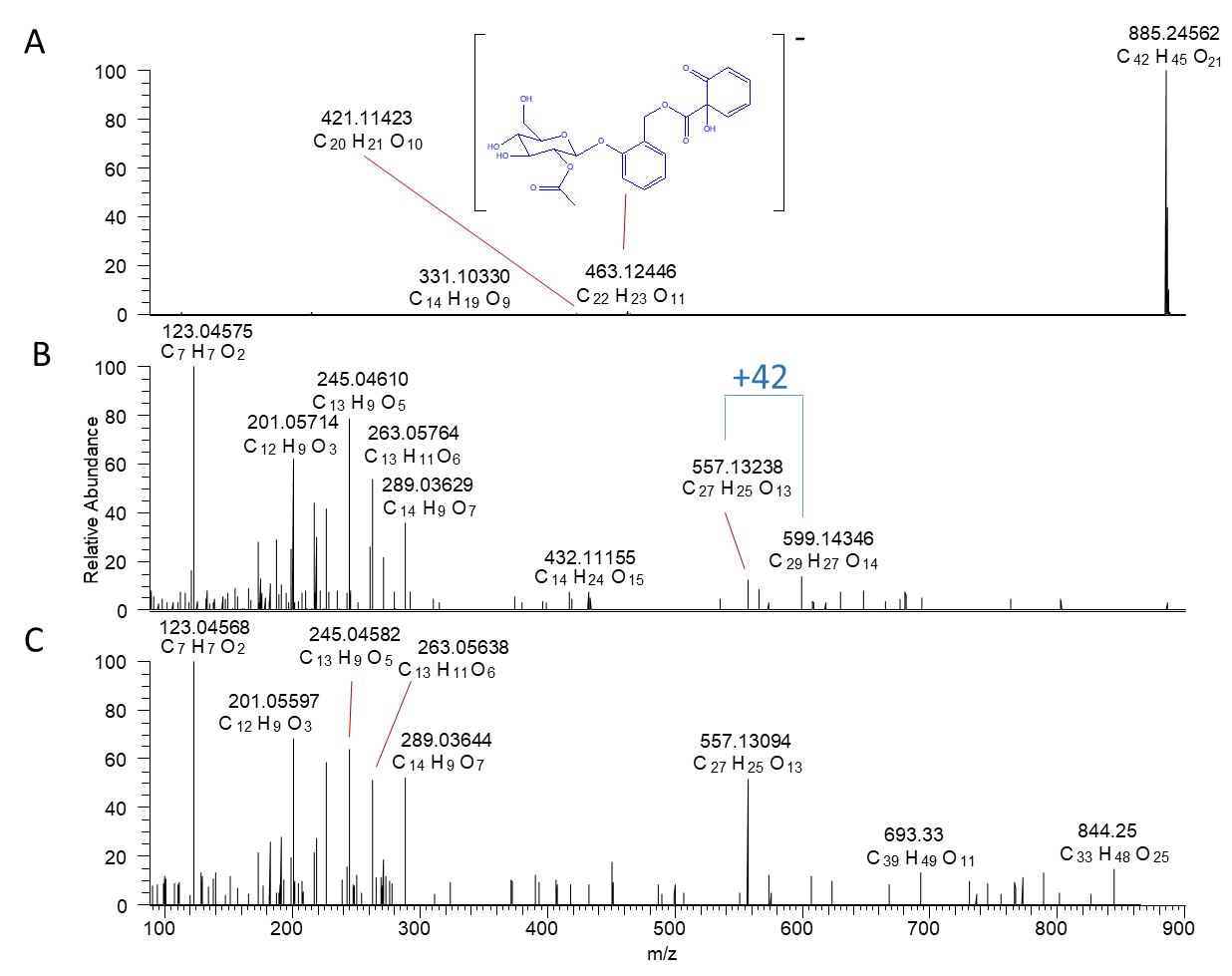


**Supplementary Figure 29.** Mass spectra of acetyl miyabeacin, **9a/9b**, at m/z 885 with retention time 27.21 min. A: MS spectrum of m/z 885 (27.21 min); B: MS-MS fragmentation of m/z 885; C: MS-MS fragmentation of miyabeacin, **3** for comparison.


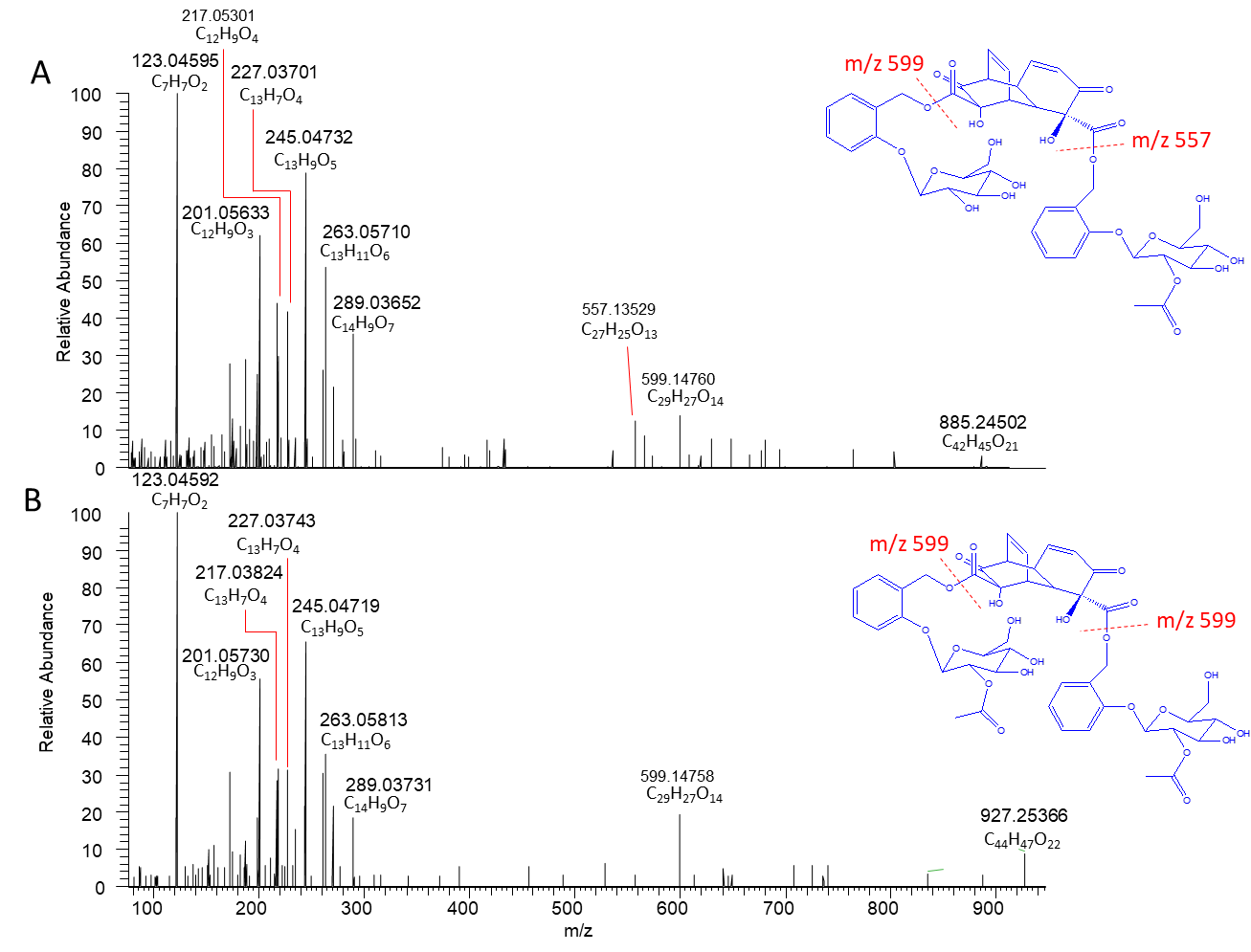


**Supplementary Figure 30.** MSMS comparison of A: acetyl miyabeacin (m/z 885) **9** and B: diacetyl miyabeacin (m/z 927) **10**. Data is generated from LC-MS (negative mode) of juvenile leaf tissue from Endurance (NWC1116) and extracted using aqueous methanol.


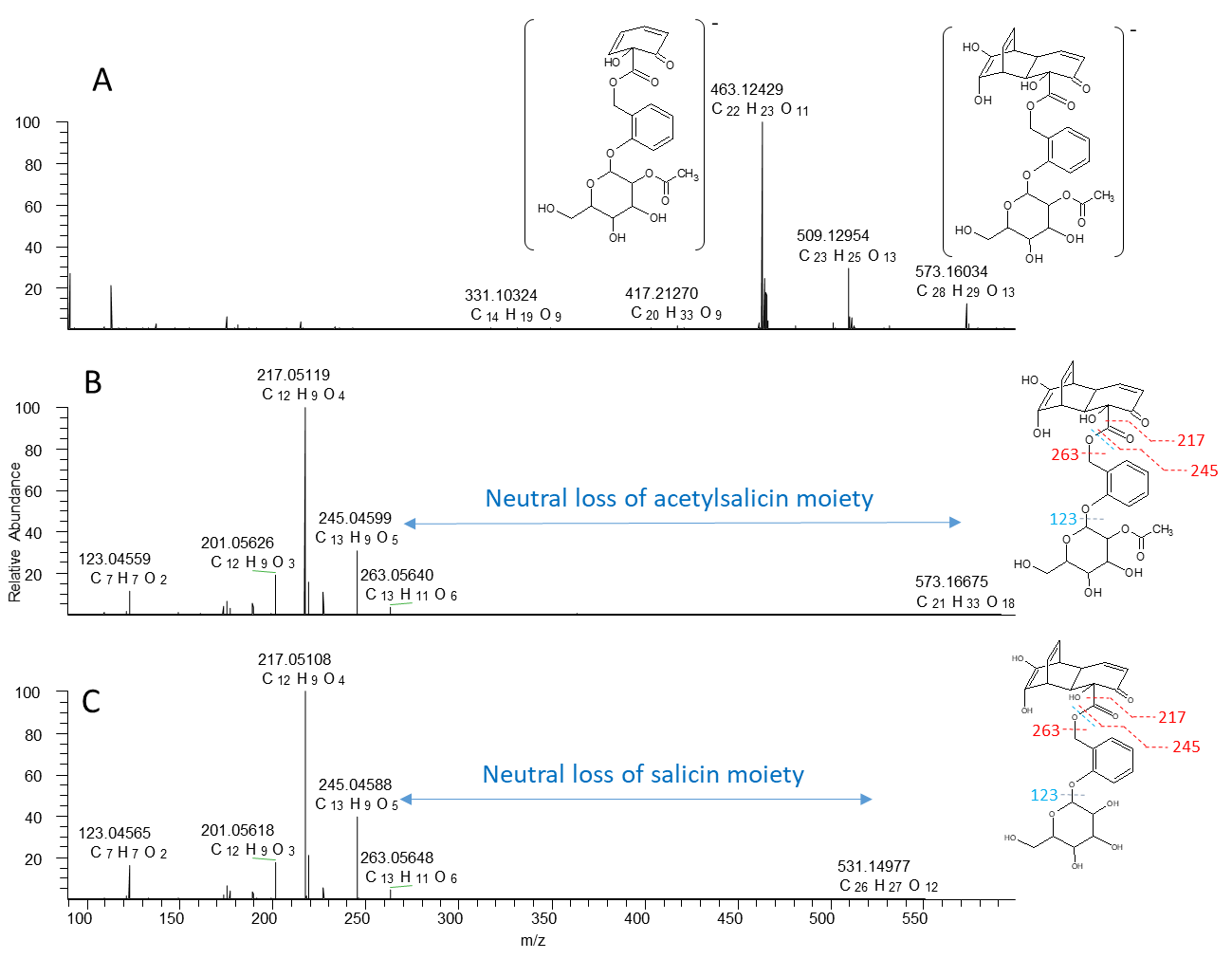


**Supplementary Figure 31.** MS and MSMS data (negative ion mode) for 2′acetyl miyabeanol **11**. A: MS spectrum of peak at 23.08 min; B: MSMS of m/z 573 [M-H]^-^ ; C: MSMS of m/z 531 (miyabeanol **7**, [M-H]^-^ ) for comparison. Data is generated from LC-MS (neg mode) of juvenile leaf tissue from Endurance and extracted using aqueous methanol.


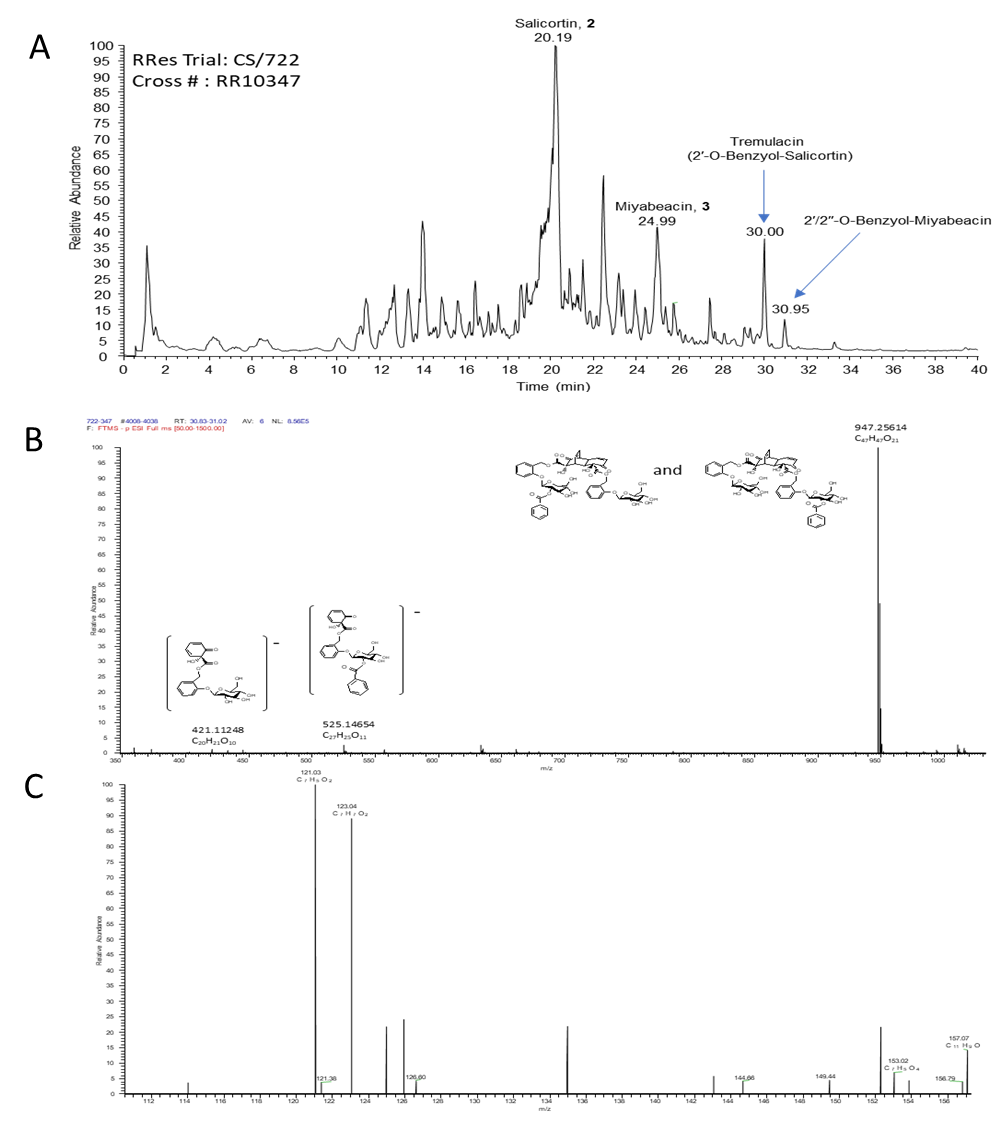


**Supplementary Figure 32**. LC-MS data (negative ion mode) of RR10347 (RR05326 (Resolution × *S. rossica*) × NWC941 (*S. miyabeana* Purpurescens)). A: Total ion chromatogram indicating benzoylated miyabeacin **16a/16b** (m/z 947); B: Mass spectrum of peak at 30.95 min. C: MSMS of m/z 947.2561.


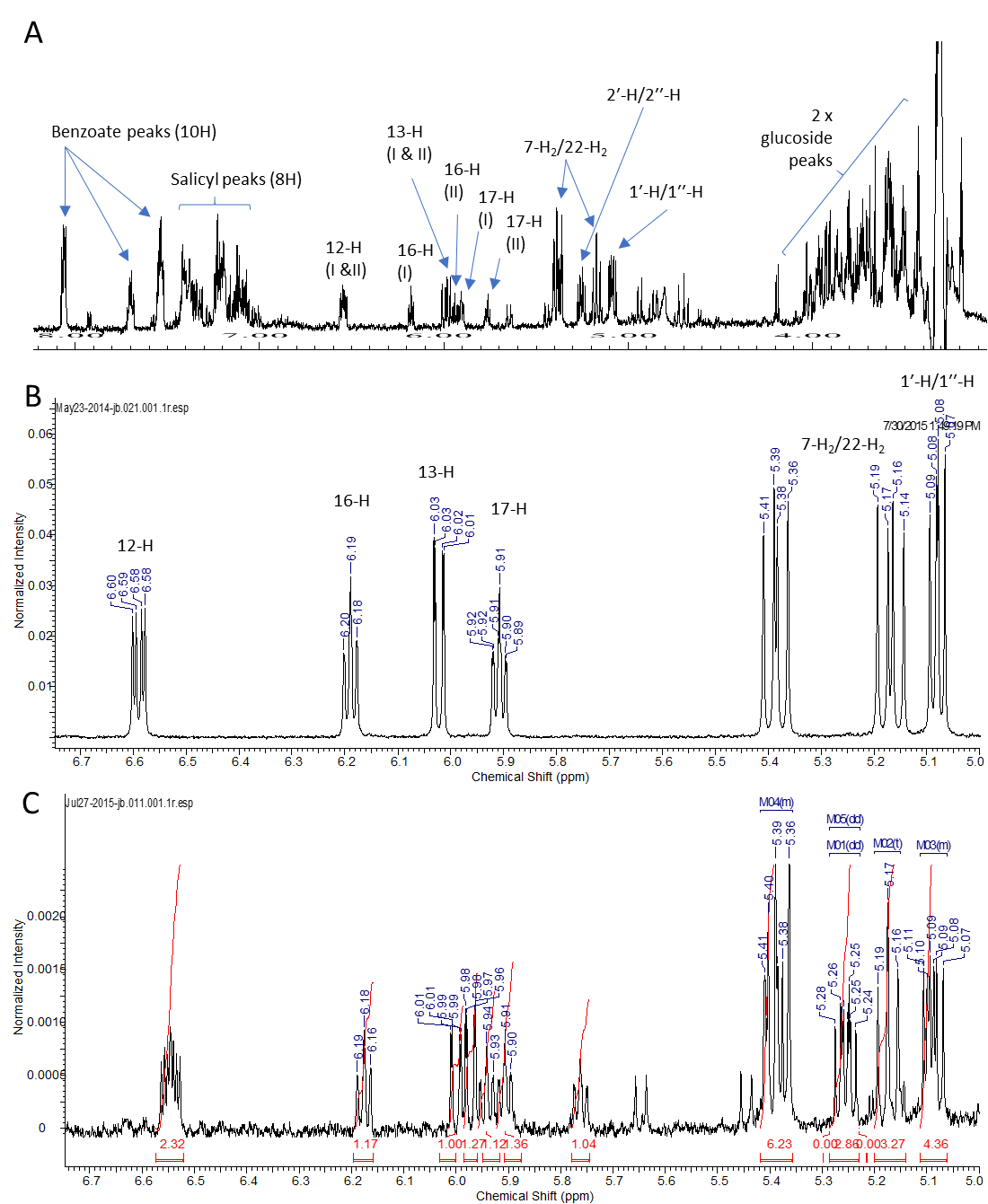


**Supplementary Figure 33**. ^1^H-NMR data of 2′-Benzoylmiyabeacin/2′′-O-Benzoylmiyabeacin **16a/16b** collected at 600 MHz in D_2_O:CD_3_OD (4:1). A: ^1^H-NMR spectrum. B: ^1^H-NMR spectrum of miyabeacin **3** for comparison (expanded region between 6.7 – 5.0 ppm). C: Expanded region of ^1^H-NMR spectrum of 2′-Benzoylmiyabeacin/2′′-O-Benzoylmiyabeacin **16a/16b** (6.7 – 5.0 ppm) for comparison with **3**. Integral values are given below peaks in red type.


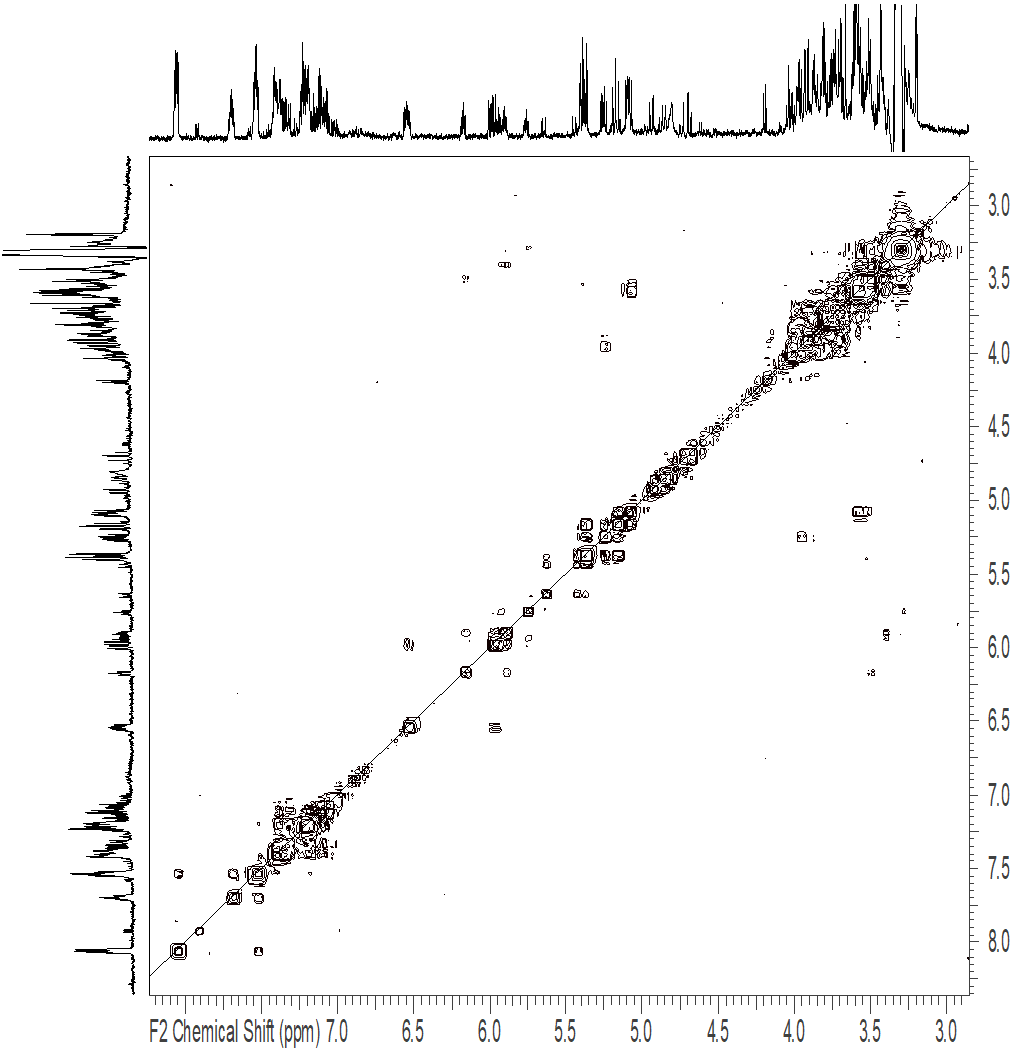


**Supplementary Figure 34.** ^1^H-^1^H COSY NMR data of 2′-Benzoylmiyabeacin/2′′-O-Benzoylmiyabeacin **16a/16b** collected at 600 MHz in D_2_O:CD_3_OD (4:1).


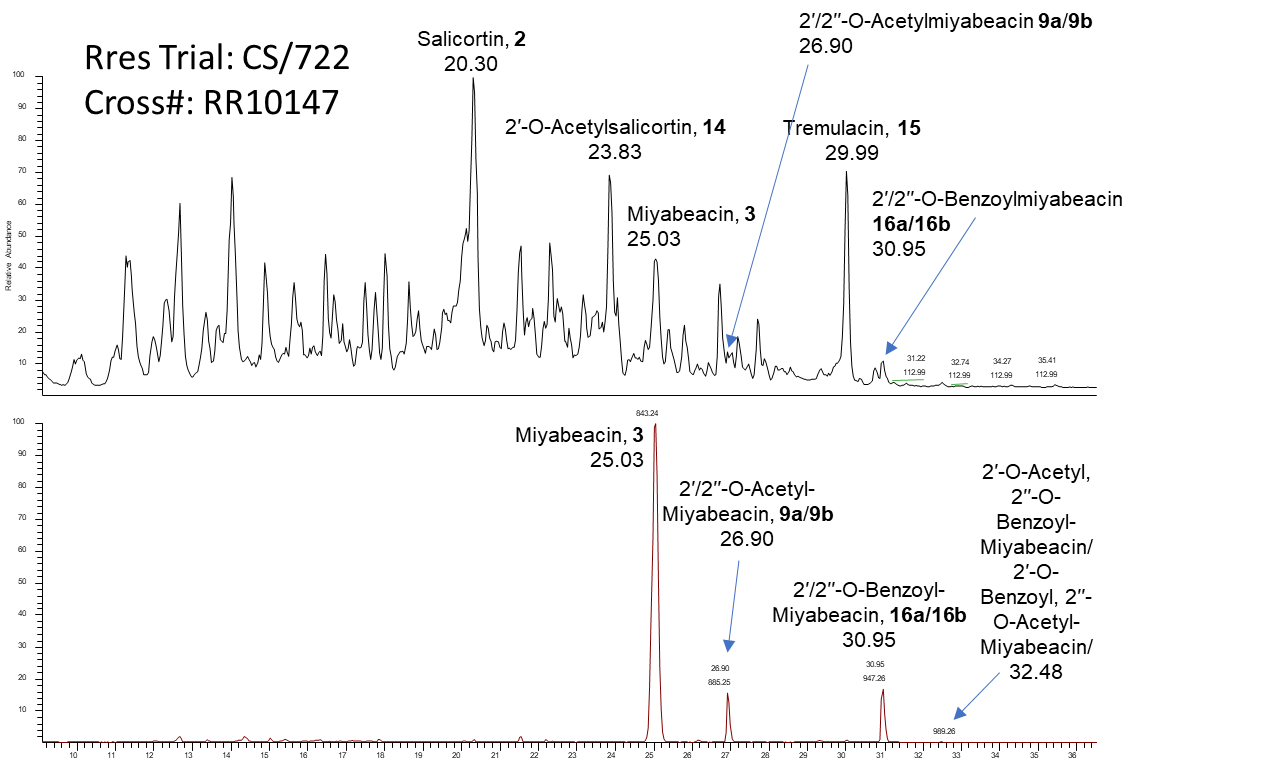


**Supplementary Figure 35**. LC-MS data (negative ion mode) of RR10147 (RR07187 (944 *S. glaucophyloides* × 577 “77056”) × RR07188 (944 *S. glaucophyloides* × 577 “77056”)). A: Total ion chromatogram; B: Extracted ion chromatogram – Mass Range m/z 800 – 1000 with identified compounds indicated
